# Efficient Computation of Functional Brain Networks: toward Real-Time Functional Connectivity

**DOI:** 10.3389/fninf.2017.00008

**Published:** 2017-02-06

**Authors:** Juan García-Prieto, Ricardo Bajo, Ernesto Pereda

**Affiliations:** ^1^Department of Industrial Engineering, Laboratory of Electrical Engineering and Bioengineering, Universidad de La LagunaTenerife, Spain; ^2^Laboratory of Computational and Cognitive Neuroscience, Centre of Biomedical Technology, UPMMadrid, Spain; ^3^Department of Statistics and Operative Research, Faculty of Medicine, Universidad Complutense de MadridMadrid, Spain; ^4^Institute of Biomedical Technology (CIBICAN), Universidad de La LagunaTenerife, Spain

**Keywords:** Functional Connectivity, complex networks, graph-theory, brain-computer interface (BCI), electroencephalogram (EEG), magnetoencephalography (MEG), neuro-feedback

## Abstract

Functional Connectivity has demonstrated to be a key concept for unraveling how the brain balances functional segregation and integration properties while processing information. This work presents a set of open-source tools that significantly increase computational efficiency of some well-known connectivity indices and Graph-Theory measures. PLV, PLI, ImC, and wPLI as Phase Synchronization measures, Mutual Information as an information theory based measure, and Generalized Synchronization indices are computed much more efficiently than prior open-source available implementations. Furthermore, network theory related measures like Strength, Shortest Path Length, Clustering Coefficient, and Betweenness Centrality are also implemented showing computational times up to thousands of times faster than most well-known implementations. Altogether, this work significantly expands what can be computed in feasible times, even enabling whole-head real-time network analysis of brain function.

## Introduction

Experimentalists have always tried to measure *better* and faster. By *better* measuring, we can think of capturing properties of the experimental data with improved sensibility. This is a very difficult task, which usually involves sophisticated paradigms and computationally intensive formulations, such as the ones described later in this work. By faster measuring, we mean reducing computational times given a fixed context. This work concentrates on the latter, increasing computational efficiency, both by reducing unnecessary calculations (i.e., adopting lower level programming languages) and by taking advantage of parallel hardware architectures. Computational efficiency allows experimental setups otherwise unreachable in common circumstances, empowering new applications and reducing developing times for new advances. One such new applications is brain-computer interface (BCI), which forges a direct real-time connection between brain and machine (van Gerven et al., 2009), with clinical applications which are becoming very relevant–see for instance an updated review (Moxon and Foffani, 2015). But also, importantly, faster measures enable a real-time feedback loop which allows interaction with brain function beneath the cognitive level and facilitates experimentally testing hypotheses, especially in combination with other techniques such as transcranial stimulation—in any of its variants (Filmer et al., 2014).

Brain Functional Connectivity (FC) and Graph-Theory related measures enable the study of long- and short-range non-linear interactions between cortical brain regions, characterizing the structural properties of networks defined by these interactions. The assessment of FC and effective brain connectivity (EC; Friston, 2011) has become one of the most active fields of research in systems neuroscience. Indeed, it is already well-established that some brain functions are not localized in specialized areas or modules but rather they reside within the interactions between brain areas (see, e.g., Horwitz, 2003; Medaglia et al., 2015 and references therein). Thus, functional specialization and integration are complementary concepts, and a thorough study of brain function through neurophysiological time-series necessarily involves the estimation of the statistical dependence between signals from different brain areas.

Some time-domain traditional measures (such as correlation coefficient) capture only linear associations between neural signals, are prone to outliers in the data and their interpretation is not always straightforward (Quian Quiroga et al., 2002). Thus in this work we focus on some measures of non-linear interaction between neurophysiological time-series. Currently, there exists a plethora of bivariate indices for this purpose (Pereda et al., 2005; Bonita et al., 2014; Wang et al., 2014). Those based on phase synchronization (PS) are among the most commonly used. Information theory-based indices such as mutual information (MI; Shannon and Weaver, 1949) are theoretically more suitable for this purpose, as they assess a more general form of statistical association (both linear and non-linear) between two time series (Kinney and Atwal, 2014), and detect, in principle, both amplitude and cross-frequency synchronization (Pompe et al., 1998). Finally, there is a third set of FC measures that are worth assessing when analysing brain activity from neurophysiological data, namely those based on the concept of *generalized synchronization* (GS; Pereda et al., 2005; Stam, 2005; Sugihara et al., 2012). Such indices require the previous reconstruction of the state spaces of the systems under study from their time series, normally using the well-known Takens' theorem (Takens, 1980), and the estimation of distances between delayed vectors in n-dimensional reconstructed state spaces. Nonetheless, in return for this added complexity, when carefully tailored (Chicharro and Andrzejak, 2009), they are able to provide information not only on the extent of dependence, but also on its directionality, making them an excellent complement to both PS-based indices and MI.

Although the brain has long been understood as a network, it was not until the end of the twentieth century that the interest in non-linear dynamical systems rolled into coupled dynamical systems, giving birth to the notion of *complex networks*. As in many other parts of nature, brain features absent at the single unit level *emerge* at the group level. It is precisely at this juncture where graph theoretical analysis, when applied to systems neuroscience, allows a richer understanding of brain function. Thus, we believe that any set of algorithms aimed at the real-time characterization of brain function should include complex networks measures.

From the stance of graph theory, brain networks can be characterized under different approaches. Many important works have contributed to settle a common terminology. Among them, Boccaletti et al. (2006) can be considered as a general and comprehensive outline of the field, whereas others such as Bullmore and Sporns (2009) provide a detailed account of the application of this methodology to neuroscience.

Given a network is a defined collection of vertices (nodes) and links (edges) between pairs of nodes, we will consider here the most typical approach, by which each node in a network represents a sensor or a brain region and links represent FC between sensors or regions, defining a *connectivity matrix* (also termed as *adjacency matrix* or *graph*; Rubinov and Sporns, 2010; Friston, 2011).

**Figure 1:**
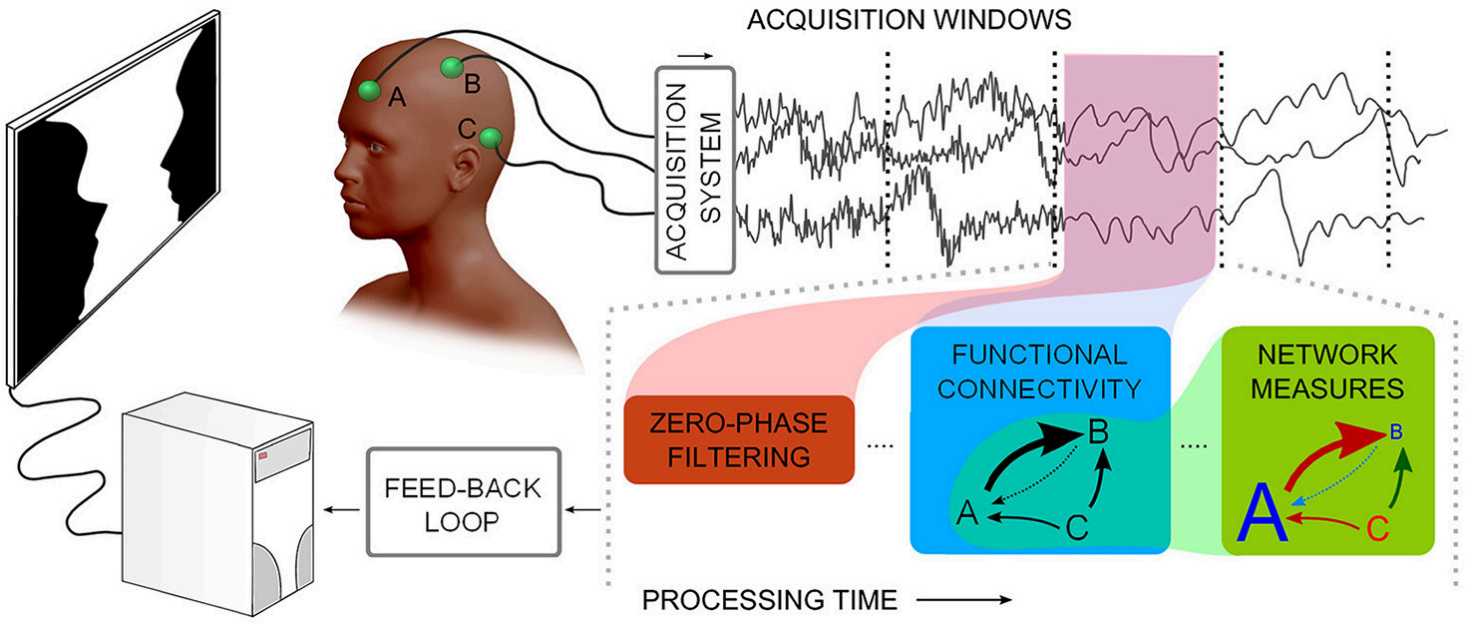
**Describes a notion of neurophysiological time-series functional network real-time analysis**. Considering multiple channels, time-series are acquired and subsequently processed in windows: noise filtering, Functional Connectivity, and graph theoretical measures need to be processed in less time than the window acquisition time.

Recently, researchers in the field have recognized the importance of developing computational platforms and toolboxes that integrate many of these FC and graph-theory indices, in a way that can make them accessible to a wider scientific community (see, e.g., Seth, 2010; Niso et al., 2013; Wang et al., 2014 for outstanding examples; see Figure 1 for a description of neurophysiological time-series functional network real-time analysis). The computational cost associated with some of these measures has also revealed the need for using high-performance computing facilities such as multicore multi-CPU and Graphics Processing Units (GPUs; Wollstadt et al., 2014) to estimate these indices in a reasonable time.

Indeed, in the framework of BCIs and neuro-feedback, if we are to estimate FC and network patterns of a subject from his/her whole head electroencephalography (EEG) (or magnetoencephalography, MEG) to decide whether to proceed with certain feedback or identify a certain outline, it is necessary to develop a computational platform that can optimally carry out the calculation. Matlab^1^, as a true standard in science, is a good example of how scripting languages empower agile development while sometimes losing computational efficiency. In this context, it seems valuable to investigate how specific functions can improve total efficiency of an application by adopting lower level programming strategies capable of running either on normal computers, embedded devices, or heterogeneous supercomputing deployments at the same time. This is probably the reason why Matlab has a wide-ranging set of tools to help interface with lower level code. Among all, external interface API^2^ which allows calling C/C++ applications as if they were built-in functions, combined with the portability of C/C++ between operating systems and computing architectures is the reason that justifies the effort we describe in this work.

Here we describe a set of tools, written in portable C/C++, which are capable of estimating the most commonly used FC indices from the families described above, as well as various complex network measures routinely used in systems neuroscience. We first provide a self-sustained methodological review of previously mentioned FC and graph-theory network indices. Secondly, we propose an implementation which is compiled using OpenMP^3^ C/C++ application program interface (Dagum and Menon, 1998) and is accessible directly through Matlab's command line interface. Finally, we compare computation efficiency—in terms of execution time- between this implementation and previous well-known open-source examples which are developed in pure Matlab code (parallelized or not with the use of Mathworks' Parallel Computing Toolbox). As we will see, by fully exploiting multicore architecture of modern processors, our implementation is, to the best of our knowledge, the most efficient one currently available for typical registration times. It allows for real-time estimation of brain FC patterns even with typical hardware architectures, without the need for expensive workstations or access to a supercomputing infrastructure. The complete source code and a Windows 64 bit precompiled implementation are freely accessible at http://juangpc.github.io/FastFC/.

## Materials and methods

As indicated above, we have included three different families of FC indices in this work: PS and GS indices and MI. Henceforth, we briefly describe each of these types of synchronization and the indices selected within them.

### PS indices

PS indices are arguably amongst the most popular methods to estimate functional connectivity between two neurophysiological signals (and more specifically, EEGs—see, e.g., Vinck et al., 2011; Porz et al., 2014; Cohen, 2015 for recent examples). The concept of PS and its estimation from time series date back to 1996, when Rosenblum et al. (1996), in a seminal paper, demonstrated that, in regimes of weak coupling, the phases of two (possibly chaotic) oscillators may synchronize even if their amplitudes remain uncorrelated. Shortly afterwards, Tass et al. (1998) demonstrated the applicability of this concept in human neuroscience. Since then, there have been many applications in different contexts, where PS indices have proven successful to estimate the degree of FC between two EEGs signals (see, e.g., Pereda et al., 2005; Vinck et al., 2011 and references therein).

Estimating PLV and PLI between two signals relies on the definition of the instantaneous phase for each signal, while ImC and wPLI rely on the computation of Fourier spectra and cross-spectrum between them:

#### Define the phase of each individual signal

Given a broad band, real valued signal *x*_*k*_(*t*) (recorded from an EEG electrode or alternatively, representing the activity of a reconstructed neural source as estimated from the scalp EEG using any source reconstruction method), we have to transform it into a complex-valued, narrow band signal using a suitable mathematical transformation—such as the Hilbert (HT), the Fourier (FT), or the Wavelet Transform. Regarding PLV and PLI, we implemented a HT-based phase definition which involves two differentiated stages. We first filtered the signals in the frequency band of interest by means of a Finite Impulse Response (FIR) filter, which was applied twice to the data, once forwards and once backwards, and after performing circular padding, to ensure, thanks to the linear response of the filter, producing exactly zero phase distortion and minimum border effects and therefore not introducing any spurious phase coupling between the signals. Then, we applied the HT to produce the analytic representation of *x*_*k*_(*t*). Thus, we first obtain the HT of this signal:

(1) $$\begin{array}{l} {\tilde{x}}_{k}\left(t\right)=\frac{1}{\pi }p.v.\int_{-\infty }^{\infty }\frac{x_{k}\left(\tau \right)}{t-\tau }d\tau \end{array}$$

where p.v. denotes the Cauchy principal value. Note that the HT is actually the convolution of *x*_*k*_(*t*) with the tempered distribution p.v. 1/π*t*, and can be obtained easily in the frequency domain as the product of both Fourier transforms. Then, the analytic representation of *x*_*k*_(*t*) is the complex-valued signal defined as:

(2) $$\begin{array}{l} x_{k}^{a}\left(t\right)=x_{k}\left(t\right)+i{\tilde{x}}_{k}\left(t\right) \end{array}$$

From (2), it is straightforward to define the phase as:

(3) $$\begin{array}{l} \phi_{k}\left(t\right)=\mathrm{atan}\frac{{\tilde{x}}_{k}\left(t\right)}{x_{k}\left(t\right)} \end{array}$$

#### Estimation of the PS indices

Given two signals, the condition of PS is formally established as

(4) $$\begin{array}{l} |n\phi_{k}\left(t\right)-m\phi_{l}\left(t\right)|<const \end{array}$$

Where || stands for absolute value. Namely, the difference between the two unwrapped phases remains bounded by a combination of positive integers m and n (the case m = n = 1 is the most usually found in practice). In practice, the estimation of PS consists in studying the distribution of the cyclic relative phase:

(5) $$\begin{array}{l} \varphi_{kl}\left(t\right)=\left(\phi_{k}\left(t\right)-\phi_{l}\left(t\right)\right)mod2\pi \end{array}$$

The most commonly used method to study this distribution is by calculating the mean phase coherence (Lachaux et al., 1999; Mormann et al., 2000; also termed *phase locking value*, PLV):

(6) $$\begin{array}{l} PLV=\left|\langle e^{i\varphi_{kl}\left(t\right)}\rangle \right| \end{array}$$

Where 〈〉 will stand for average value here on. Defined in this way (6), ranges between 0 (no PS) and 1 (perfect PS), although in real life data, its value is always in between these two extreme ones. In the analysis of neurophysiological data, especially EEG, a known issue is the influence on PLV of volume conduction effects and the choice of the reference (see, e.g., Marzetti et al., 2007; Porz et al., 2014; Cohen, 2015; Chella et al., 2016 for recent studies dealing with this issues). Consequently, Nolte et al. (2004) proposed the *imaginary part of the coherency* (ImC) as an index of PS that showed no influence of either common references or volume conduction (see specifically Marzetti et al., 2007; Chella et al., 2016 for in depth analysis of the reference influence in the ImC and interesting ways to prevent problems), due to the fact that these problems would affect separate sensors with negligible time delay, which is true given the quasi-stationary description of Maxwell equations holds for typical MEG and EEG signals. Thus, the ImC index is defined as the imaginary part of the Coherency, which is in turn, defined as:

(7) $$\begin{array}{l} C=\frac{\langle X\rangle }{\sqrt{\langle {\left|Z_{1}\right|}^{2}\rangle \langle {\left|Z_{2}\right|}^{2}\rangle }} \end{array}$$

where Z is the Fourier Spectra of each signal and X is the cross-spectrum between each pair.

Stam et al. (2007) suggested an alternative index to overcome the strong influence of the phase of the coherency present in ImC. This index termed *phase lag index* (PLI), is also insensitive to zero lag coupling and is therefore not affected by volume conduction. PLI is defined as

(8) $$\begin{array}{l} PLI=|\langle sign\left(\varphi_{kl}\left(t\right)\right)\rangle | \end{array}$$

where *sign*(*x*) is the sign function {+1∀*x* > 0;−1∀*x* < 0}.

Vinck et al. (2011) further improved this definition by introducing the *weighted PLI* (wPLI), giving a different weight to each of the values of the relative phase (the closer they are to zero, the most likely they are to be affected by measurement noise, and the lower the weight they receive):

(9) $$\begin{array}{l} wPLI=\frac{\left|\langle img\left(X\right)\rangle \right|}{\langle \left|img\left(X\right)\right|\rangle }=\frac{\left|\langle |img\left(X\right)|sign\left(img\left(X\right)\right)\rangle \right|}{\langle \left|img\left(X\right)\right|\rangle } \end{array}$$

where *img*(*X*) denotes imaginary part of the cross-spectrum.

Although, in principle, PLI or wPLI should be the optimal choice given their properties, there exists neurologically meaningful couplings between brain areas that take place at zero lag (Vicente et al., 2008), and these would be missed by both PLI and wPLI. Not surprisingly, recent results (Christodoulakis et al., 2014; Porz et al., 2014) show that there is no such thing as a perfect PS index, and indeed PLV can sometimes be better than either PLI or wPLI at uncovering differences between groups or conditions. As described later, we included all four indices in the present implementation.

#### Estimation of the significance of the indices

Finally, as an additional step, it is always convenient to estimate whether a given value of any of the PS indices described above (or any other FC index, for that matter) is significantly different from zero, i.e., it is the result of true FC between the data or just a spurious value due to, for example, shortness of the data. For this purpose, different types of surrogate data can be used (Thiel et al., 2006; Romano et al., 2009), yet the construction of a good set of such surrogates and the subsequent estimation of any of the PS indices from this set is unfeasible if we are to keep the computational time within a certain bound. There is, however, an alternative that can be applied in the specific case of the PLV, and which we also include in this work. It is based on the fact that the PLV is also the mean resultant length of the circular distribution of the relative phase (Mardia and Jupp, 2000). For this measure, there exist well-established tests of uniformity of the distribution, which allow the estimation of the probability—*p-value*—for a given length of the relative phase series, given that the corresponding PLV value has been obtained by chance. In the corresponding function, we use the approximation due to Wilkie (1983) of the Rayleigh test, whereby the probability is of PLV being greater of a certain value *K* for *N*_*samples*_ is estimated as:

(10) $$\begin{array}{l} \mathrm{Pr}(N_{samples}PIV^{2}>K)=\exp\{[1+4N_{samples}+4(N_{samples}^{2}\\ \;{-N_{samples}K)]}^{1/2}-(1+2N_{samples})\} \end{array}$$

Although this approximation is based on the assumption that consecutive values of the phases are approximately independent, which is not completely fulfilled, it has the advantage of providing a continuous, parametric estimation of the *p-value* associated to each PLV, without the need to construct the surrogates. These *p-values* can be later corrected for multiple comparisons using, e.g., False Discovery Rate (Benjamini and Hochberg, 1995), thereby providing a good estimation of the significance of each PLV.

### Mutual information

The MI is, from a theoretical point of view, the best option to determine the degree of FC between two signals under the most general interpretation (Kinney and Atwal, 2014). In fact, whereas the calculation of the PS and GS indices require previous transformation of the signal as pre-processing steps, in the case of the MI it is only necessary to estimate the individual (marginal) and the joint entropies of the data. As we will see, however, this apparently very simple concept is not without its complications.

Formally, the MI for a discrete pair of random variables (signals) X and Y is defined as:

(11) $$\begin{array}{l} MI\left(X,Y\right)=\underset{y_{i}\in Y}{\sum }\underset{x_{i}\in X}{\sum }p\left(x,y\right)\log\left(\frac{p\left(x,y\right)}{p\left(x\right)p\left(y\right)}\right) \end{array}$$

where *p*(*x*), *p*(*y*) and *p*(*x, y*) are the marginal probability distribution functions and the joint marginal one, respectively. For independent processes, the joint probability factorizes as the product of both marginal probabilities, the logarithm and therefore the MI also equals zero. If, on the other hand, the processes are not independent, but there is any kind of statistical dependence between them, the joint probability is higher than the product of the marginal ones and the MI is greater than zero. Alternatively, the MI can be also defined as a combination of entropies:

(12) $$\begin{array}{l} MI\left(X,Y\right)=H\left(X\right)+H\left(Y\right)-H\left(X,Y\right) \end{array}$$

Either way, the practical estimation of the MI and its application in the context of FC and complex networks presents two problems: estimation of probabilities from finite time-series and normalization.

#### Estimation of the probability from data

This is arguably the most complicated part of the calculation of the MI. The naïve estimation of the probabilities, which is based on the binning of the ranges of X and Y, implicitly assumes that the relative frequency of a given value estimated from this binning equals its probability. Although it is the fastest implementation (Wang et al., 2014), as it is based on the individual and the joint histogram, it is also known to produce biased estimations of the MI (Kraskov et al., 2004; Hlavackova-Schindler et al., 2007), because such equality is only valid in the limit of infinite data samples (the well-known Law of Large Numbers). Even sophisticated binning strategies, such as using bins of unequal size to maximize the individual entropies; do not completely eliminate this bias in practical applications. Therefore, more elaborated estimations of the probabilities are called for, in order to eliminate such bias. After extensive search in previous literature and testing of different options, we decided to follow the implementation of Kraskov et al. (2004), which is based on an estimation of the MI that makes use of the k-nearest neighbors' statistics for the estimation of the entropies. According to the latest theoretical as well as practical results, Kraskov's implementation has proven to be the most adequate algorithm for practical applications, showing statistical properties that excel those of more modern and sophisticated estimations of correlation such as the *maximal information coefficient* (MIC).

The practical calculation of the MI is complicated. Firstly, the calculation depends on the reliable estimation of both the marginal and the joint probability density functions, which is known to be a very complicated task for short, noisy time series (Kraskov et al., 2004; Hlavackova-Schindler et al., 2007; see also Kugiumtzis and Kimiskidis, 2015). Secondly, the MI is non-normalized, which means that it is (theoretically) zero for completely independent signals but, unlike most other FC measures, it is not 1 for completely dependent signals. Should these complications be overcome, however, the MI (and specifically its estimation based on k-nearest neighbor's strategies) is deemed as one of the most powerful (both in general terms and statistically speaking; Kinney and Atwal, 2014) FC measures at hand.

#### Normalization of the MI

As commented above, one problem with the MI is that, whereas it is (theoretically at least) zero for completely independent data, its value is not bounded in the case such independence does not hold. Instead, its upper limit for completely dependent signals depends on the individual entropies of each data signal (which is exactly the problem that the MIC was supposed to solve). This implies that a value of the MI of, say, 0.3 for two signals may in fact result from a higher *connection* than a value of 0.25 from another two (possibly more complex) ones. This acute issue may become serious if we are to compare the degree of FC in two different situations or populations based solely on MI.

Different normalization procedures are possible. Among them, two possibilities are common, both making use of the individual entropies as normalizing factors. They are the symmetric uncertainty (Witten et al., 2011), defined as:

(13) $$\begin{array}{l} U\left(X,Y\right)=2\frac{MI\left(X,Y\right)}{H\left(X\right)+H\left(Y\right)} \end{array}$$

and the total correlation

(14) $$\begin{array}{l} \frac{MI\left(X,Y\right)}{\min\left[H\left(X\right),H\left(Y\right)\right]} \end{array}$$

which are both normalized to 1.

### GS indices

As remarked in the Introduction, at the beginning of the 1980s, the Dutch mathematician F. Takens proved a theorem (Takens, 1980) whereby, under general conditions, it is possible to reconstruct the state space of a complex dynamical system (even non-linear systems in chaotic regime) using the consecutive values of one of its time series. Indeed, he demonstrated that, given the time series *x*_*k*_(*t*), the delayed vectors defined as:

(15) $$\begin{array}{l} X_{i}=\left(x_{k}\left(i\right),x_{k}\left(i+\tau \right),\ldots ,x_{k}\left(i+\left(m-1\right)\tau \right)\right) \end{array}$$

are equivalent to the original state vectors. In (8), *m* is the so-called embedding dimension, which has to be at least equal to the dimension of the original system, and τ is the delay time, which has to ensure that two consecutive components of the vector are (almost) independent. Usually, *m* is estimated using the heuristic approach termed false nearest neighbors, whereas τ can be estimated using the autocorrelation or the auto-MI function of the data (Kantz and Schreiber, 2004).

In the case of FC studies, this idea allows for a sophisticated assessment of the degree of statistical dependence between two signals, x and y. For this purpose, delayed state vectors *X*_*i*_ and *Y*_*i*_ are first reconstructed from *x*_*k*_(*t*) and *y*_*k*_(*t*), as in. Then, let *a*_*i, j*_ (respectively, *b*_*i, j*_) be the time indices of the *k* nearest neighbors of *X*_*i*_ (resp. *Y*_*i*_). The existence of FC between both time-series entails that there exists a functional transformation between the state space of *X*_*i*_ and *Y*_*i*_, and therefore if two states are close in the state space of *X*_*i*_, they are also close in *Y*_*i*_ 's. The amount of connectivity can be measured using different bivariate indices (Pereda et al., 2005; Niso et al., 2013). We have included several of them, which we describe henceforth.

#### Similarity index S

It is the earliest developed index of GS from two time series (Arnhold et al., 1999). It is defined as:

(16) $$\begin{array}{l} S^{\left(k\right)}\left(X|Y\right)=\frac{1}{N}\overset{N}{\underset{i=1}{\sum }}\frac{R_{i}^{\left(k\right)}\left(X\right)}{R_{i}^{\left(k\right)}\left(X|Y\right)} \end{array}$$

where $R_{i}^{\left(k\right)}\left(X\right)$ is the average Euclidean distance between the *X*_*i*_ and its *k* nearest neighbors, with time indices *a*_*i, j*_, and $R_{i}^{\left(k\right)}\left(X|Y\right)$ is the same but calculated considering the indices of the nearest neighbors of *Y*_*i*_, this is, *b*_*i, j*_. The existence of GS between *X*_*i*_ and *Y*_*i*_ produces that these *k* so-called *Y*_*i*_ *-conditioned neighbors* of the reconstructed vectors of *X*_*i*_ are closer to them than should be expected by chance, thus the ratio in and the index itself are close to 1 (and equal to 1 for identical signals). On the other hand, if there is no GS, the *Y*_*i*_ *-conditioned neighbors* are equivalent to vectors randomly chosen in the attractor, and the index is close (but not equal) to 0. The corresponding version in the reconstructed state space of *Y*_*i*_, *S*^(*k*)^(*Y*|*X*) can be calculated analogously.

#### H index

The similarity index above is the simplest implementation of a bivariate GS index relying on the comparison between nearest and conditioned neighbors. However, it has been shown (Schmitz, 2000; Pereda et al., 2001; Quian Quiroga et al., 2002) that it is not the best choice when there is special interest on the directionality of the interaction. Besides, its value for completely independent signals is not zero, but depends on the average size of the reconstructed attractor, which in turn changes with the complexity of each signal as well as with the number of available data points. Instead, a variation of this index, termed *H*, has been proposed, which is defined as:

(17) $$\begin{array}{l} H^{\left(k\right)}\left(X|Y\right)=\frac{1}{N}\overset{N}{\underset{i=1}{\sum }}\log\left(\frac{R_{i}\left(X\right)}{R_{i}^{\left(k\right)}\left(X|Y\right)}\right) \end{array}$$

where *R*_*i*_(*X*) is the average distance between *X*_*i*_ and any other reconstructed vector in *X*_*i*_ (the so-called radius of the attractor). Clearly, *H* equals 0 for independent signals, no matter the size of the attractor, yet it is not normalized (i.e., its upper bound does depend on the individual signals).

#### M index

Addressing some of the problems inherent to S and H, a more appropriate way of normalizing the distances was defined (Andrzejak et al., 2003):

(18) $$\begin{array}{l} M^{\left(k\right)}\left(X|Y\right)=\frac{1}{N}\overset{N}{\underset{i=1}{\sum }}\frac{R_{i}\left(X\right)-R_{i}^{\left(k\right)}\left(X|Y\right)}{R_{i}\left(X\right)-R_{i}^{\left(k\right)}\left(X\right)} \end{array}$$

In this case, for independent signals, the numerator (and therefore the index) equals zero, whereas for identical signals, the ratio equals 1. Still, the difference between and the analogous expression in the state space of *Y*_*i*_ is sometimes misleading when drawing conclusions on the directionality of the interaction.

#### L index

Recently, Chicharro et al. (Chicharro and Andrzejak, 2009) proposed an improved version of the M index above, which uses ranked statistics of distances, instead of directly using space state distances, thus granting the L index with greater robustness against outlier situations, a lower noise susceptibility and an improved sensitivity specially interesting when characterizing low coupling.

(19) $$\begin{array}{l} L^{\left(k\right)}\left(X|Y\right)=\frac{1}{N}\overset{N}{\underset{i=1}{\sum }}\frac{G_{i}\left(X\right)-G_{i}^{\left(k\right)}\left(X|Y\right)}{G_{i}\left(X\right)-G_{i}^{\left(k\right)}\left(X\right)} \end{array}$$

Here, $G_{i}^{\left(k\right)}\left(X\right)=\left(k+1\right)/2$ are the average rank distances between any *X*_*i*_ and its *k* nearest neighbors and *G*_*i*_(*X*) = *N*/2 is the average ranked distance metric for the remaining states. The ranking procedure ensures that these two distances are constant for all *i* and for every signal, as they only depend on *N* and *k*. The only remaining distance, which is the one affected by the degree of FC between the signals, is $G_{i}^{\left(k\right)}\left(X|Y\right)$. Hence L, being a ranked version of M, has shown better robustness against noise.

### Complex network analysis

Considering a *graph* is a structured collection of *N* nodes and *L* links between them, a functional brain network can be defined based on neuro-dynamical interactions between brain regions. These interactions are here defined based on FC values, and as FC indices—normalized or not—are real-valued, the network is defined as weighted. Thus, a functional brain network is represented by its connectivity matrices, where rows and columns denote nodes and matrix entries denote links.

While discussing optimal methodological procedures for extracting brain networks from neurophysiological signals, it is clear that histology studies of brain networks come to the conclusion that the nervous system does not appear as a completely random nor a completely regular reality (DeFelipe, 2010). It is rather a set of *random patterns* that probably drive the brain through its development and therefore express themselves through their function. Although this expression allows for much controversy on the actual procedure (Bialonski, 2012; Zanin et al., 2012; Hutchison et al., 2013). Weak and non-significant links are believed to represent spurious connections; these links tend to obscure the backbone topology of the network, as defined by strong and significant connections, and as a result are often discarded. For simplicity, sometimes FC indices are thresholded, making the network binary as the adjacency matrix is populated with {0, 1} values.

In this work we will focus on a reduced set of commonly used weighted network indices. These are: *strength* (S), *Clustering coefficient* (C), *shortest path length* (L), and *betweenness centrality* (B). They enable the study of network properties such as node importance (S), functional segregation (C), functional integration (L), *small-worldness* (C and L), network motifs (L), network centrality (B), and network resilience (L and B). Concepts thoroughly described in Rubinov and Sporns (2010), a study where one of the most well-known open-source toolbox for brain network analysis Brain Connectivity Toolbox^4^ (BCT) is described; our implementation of complex network measures derives directly from it.

#### Strength

Considering a link (*i, j*) as a *connection* between nodes *i* and *j* (*i, j* ∈ *N*), defined by a weight *w*_*i, j*_ ∈ [0, ∞) or [0, 1] for normalized FC indices (should *binarization* be required, *a*_*i, j*_ ∈ {0, 1} is a binary weight related to the existence of a link between nodes *i* and *j*). As described in Rubinov and Sporns (2010), the number of links in the network is $l^{b}=\sum_{i,j\in N}a_{i,j}$, or $l^{w}=\sum_{i,j\in N}\delta \left(w_{i,j}\right)$, where here and henceforth the superscripts *b* and *w* stand for binary and weighted normalized networks, respectively and δ = 1 *if* (*w*_*i, j*_ ≠ 0). The strength S of each node is understood as the sum of its links. This way:

(20) $$\begin{array}{l} S_{i}=\underset{j\in N}{\sum }a_{i,j} \end{array}$$

for binary networks, and

(21) $$\begin{array}{l} S_{i}^{w}=\underset{j\in N}{\sum }w_{i,j} \end{array}$$

for weighted networks.

The global S of a network is the average S of all its nodes. Interestingly, the mean network *strength* can be used as a measure of the *density* of the network, and along with network *degree*, many network features can be measured through their influence on the degree and strength distribution (Boccaletti et al., 2006).

#### Clustering coefficient

Focusing on how the network segregates information (usually interpreted as a measure of subnetwork specialization) the *C* of the network is defined as

(22) $$\begin{array}{l} C^{w}=\frac{1}{n}\underset{i\in N}{\sum }C_{i}^{w}=\frac{1}{n}\underset{i\in N}{\sum }\frac{2t_{i}^{w}}{k_{i}\left(k_{i}-1\right)} \end{array}$$

*C*_*i*_ represents each node's C, with (*C*_*i*_ = 0 for *k*_*i*_ < 2), *k*_*i*_ is the degree of the node *i* and the number of triangles $t_{i}^{\text{w}}$ around a node *i*, is defined by the geometric mean of triangles around it:

(23) $$\begin{array}{l} t_{i}^{w}=\frac{1}{2}{\underset{j,h\in N}{\sum }\left(w_{ij}w_{ih}w_{jh}\right)}^{1/3} \end{array}$$

C describes the likelihood that the neighbors of a vertex are also connected among them. It is the fraction of triangles around a node, and is equivalent to the fraction of nodes' neighbors that are neighbors of each other, thus quantifying the inclination of network elements to form local clusters. The notion of triangles is important, since it is directly related to the robustness and error tolerance of the network (Boccaletti et al., 2006), and help understand how well-grained subnetworks of neighbors are.

#### Shortest path length

Following Watts' definitions (Watts and Strogatz, 1998) of network integration measures, we can study how easily information can spread through the network. For which the most characteristic index is the *shortest path length*, which is directly related to the robustness and error tolerance of the network (Boccaletti et al., 2006).

To define this index, it is necessary to first introduce the notion of *path*, as a sequence of linked nodes that never visit a single node more than once^5^. The *characteristic path length* is then defined as the average shortest path length in the network:

(24) $$\begin{array}{l} d_{ij}^{w}=\underset{a_{uv}\in gi\to j}{\sum }f\left(w_{uv}\right) \end{array}$$

where *f* is a map defining link length (inversely related to weights) and *g*_*i*→*j*_ is the shortest weighted path between *i* and *j*. Considering a global network average of this metric, we measure how well the network integrates information with the index L:

(25) $$\begin{array}{l} L^{w}=\frac{1}{n}\underset{i\in N}{\sum }L_{i}^{w}=\underset{i\in N}{\sum }\frac{\underset{j\in N}{\sum }d_{ij}^{w}}{n-1} \end{array}$$

with $L_{i}^{w}$ being the average distance between node *i* and all other nodes.

By their indices L and C, the structural properties of a graph can be quantified. L measures a global property, while C measures the locality of a neighborhood (Watts and Strogatz, 1998). Typically, in neuroscience, noise and other methodological problems yield networks with many vertices where every node is connected to every other. By studying the amount of C and L we can characterize the small-worldness (Watts and Strogatz, 1998) and other features like efficiency and randomness of the network.

#### Betweenness centrality

It is the number of all shortest paths in the network that contain a given node. Nodes with high values of betweenness centrality participate in a large number of shortest paths. It can be defined as (e.g., Freeman, 1979)

(26) $$b_{i}=\frac{1}{\left(N-1)(N-2\right)}\sum_{\begin{array}{l} \;h,j\in N\\ h\neq j,h\neq i,j\neq i \end{array}}\frac{\rho_{hj}(i)}{\rho_{hj}}$$

where ρ_*hj*_ is the number of shortest paths between nodes *h* and *j*, and ρ_*hj*_(i) is the number of shortest paths between these two nodes that pass through node i. High centrality emphasizes that a node can reach others with a relatively short route, this is, it is a vertex closely connected to others.

## Results

We pursue contributing to the field not only by committing a set of tools for FC and network analysis, but also by giving the reader an opportunity to predict plausible computational time improvement for the set of FC and network measures we are presenting, under their specific hardware implementation. Given the wide range of possibilities among neurophysiological techniques and specific procedures within them, in terms of numbers of sensors, sampling frequencies and time-series duration, we will express our results in terms of number of samples, and number of sensors when appropriate, thereby avoiding any loss of generality.

We would like to emphasize that improvements correlate with the number of samples or sensors considered. However, we do want to present our results under challenging, yet reasonably common conditions as those of real-time EEG or MEG processing. This way, we feel that 32 or 64 channels each with 1000 samples represent a common situation. Regarding network analysis, challenges rise with the number of nodes in the network; we therefore tested our implementation up to 4000 nodes, but note that for sensor or source-reconstructed level analysis, the number of nodes in the brain FC network is typically around 10^2^.

### Hardware aspects

Computational times certainly do depend on the hardware architecture of the computer doing the work. Therefore, we report here results for two different hardware setups. The first one (setup A) consists of a high-end professional server graded setup with a dual CPU board with two Intel Xeon processors (10 physical cores each). The second one (setup B) is based on a consumer graded computer Intel-i7 CPU (with 4 physical cores). Both setups are thoroughly described in the Supplementary Material. This should help the reader estimate execution time improvements for his/her specific setup. Hyper-threading was enabled in both cases.

### Software aspects

Different versions of each implementation have been developed as Matlab scripts and C/C++ implementations. Matlab MEX-files development application interface has enabled us to write these custom C/C++ programs to be called as regular Matlab functions, adding the convenience of the Matlab environment to a more efficient execution. Regarding this implementation, whenever possible, we only made use of the standard C library, thus making the development portable and platform independent. However, in the case of zero-phase distortion filtering and PS indices we do use the FFTW library for computing the discrete FT and HT of arbitrary input sizes. This library is free software, freely distributed under the terms of the GNU General Public License with versions for all major operating systems, and according to benchmarks publicly available^6^, FFTW's performance is typically superior to that of any publicly available FFT software and is even competitive with vendor-tuned codes.

In terms of precision, we are confident that the typical signal-to-noise ratio in neurophysiological time-series makes the use of double precision floating-point (DPFP) arithmetic excessive, thus we have developed all functions using internally single precision floating-point (SPFP) arithmetic, allowing for some savings in terms of memory, memory bandwidth, and perhaps a few processing cycles.

Regarding the MI, our implementation is based on previously existing software written in C++ for Linux operating system. Therefore, we have maintained C++ as the language in this case.

Shared-memory parallelism in C/C++ has been achieved through the set of compiler directives and library functions specified by the OpenMP C/C++ application program interface in the latest version supported by the compiler^7^, consequently maintaining portability. We decided to use OpenMP over other solutions such as POSIX threads or native windows thread interface, to preserve such compatibility between operating systems and ecosystems. We are aware that FFTW is capable already of multithread execution, however to avoid possible inconsistencies between versions of OpenMP in different platforms we have decided to use a single-thread version of the FFTW library and parallelize on top of that.

Following each implementation, we proceed to experiment with variable data arrangements. As explained before, instead of considering explicit sampling frequencies, we report results in terms of execution times for various sample lengths and channels configuration, leaving the reader to accommodate these to specific acquisition setups. We analyzed random time-series consisting of 8 to 200 channels, and 10^2^ up to 10^4^ samples per channel for each FC index and each implementation (see figures in the Online Resource for additional information).

### PS indices PLV, PLI, and wPLI

PS indices are fairly easy to implement in practice, yet the need for different pre-processing steps (filtering and phase extraction for PLV and PLI and Fourier spectra for ImC and wPLI) might create some hassle. We will briefly review the currently available open-source versions of PS indices and will then show the results in terms of speed-up of this implementation in both setups.

#### Currently available versions

To the best of our knowledge there are three different open-source implementations of the PS indices, thus, we used these implementations to check and validate our hypothesis of efficiency improvement:

##### DAMOCO

Rosenblum and colleagues have developed a Matlab toolbox for Data Analysis with Models of Coupled Oscillators^8^. This toolbox includes the implementation of PLV, filtering, and Hilbert transform. They are all in plain Matlab code not optimized for multichannel data. PLI and wPLI are not included, nor the estimation of the statistical significance of the PLV index.

##### FieldTrip

The popular toolbox for EEG/MEG analysis^9^ includes filtering and phase estimation scripts, as well as estimators of the four PS indices (without considering PLV's significance). These implementations are written in Matlab, and most importantly, the calculation requires input data to be formatted in FieldTrip data files, which adds further constraints limiting its convenience in third-party applications.

##### EEGLab

This globally used toolbox for EEG (and sometimes also MEG) analysis^10^ includes filtering and phase estimations scripts, as well as estimators of PLV and PLI (with no estimation of their significance) all developed in Matlab language. Again, the same issues as in the case of FieldTrip, it is required to work with data in EEGLab format.

#### Our implementation

We have developed two separate functions, one accounts for PLV and PLI and a second one that computes wPLI and ImC. This is due to the fact that both PLV and PLI are most efficiently calculated based on the analytic signal of each sensor, while wPLI and ImC are based on the spectrum estimation for which a non-overlapping set of computations is required.

Regarding PLV and PLI, firstly, a Matlab implementation was programmed. These are fairly direct algorithms which can fit in a few lines of code. This version was subsequently parallelized in different ways, by using Matlab's built-in *bsxfun* function and/or Mathworks' Parallel Computational Toolbox *parfor* structure. Finally, a multithreaded C-mex file implementation was also developed. This last version dynamically selects the optimum number of threads (typically the number of logical processors) in the computer. The implementation of PLV can very well-constitute an illustrative example on how much effort must be devoted into a C-mex implementation against a Matlab implementation, as the dozen lines of code of the latter are equivalent to the 250 lines of code of the former. At the same time, it might constitute a just as illustrative example of the efficiency benefits of using C-mex implementation through Mathworks' MEX-interface, in a worst case scenario. Matlab's PS implementation is quite optimized indeed, but still, C-mex implementation is around an order of magnitude faster than Matlab's. We therefore conclude that a low margin for improvement for an algorithm in C vs. Matlab is around an order of magnitude and much lower than in other cases.

Our implementation accounts for the border effect due to HT, by practically discarding phases within a distance from the beginning or the end of the time-series. This feature is specified when calling the function, defining the number of samples since the beginning and before the end of the time series, to be alienated from further PS analysis.

Our final multithreaded C-mex implementation, allocates at most 6·*N*_*samples*_ · *N*_*sensors*_ SPFP Bytes^11^ in main memory. This can be one of the main advantages for high-channel-density setups, because as our implementation is based on shared memory parallelism, there is very little memory duplication between threads as opposed to PCT's workers.

**Figure 2:**
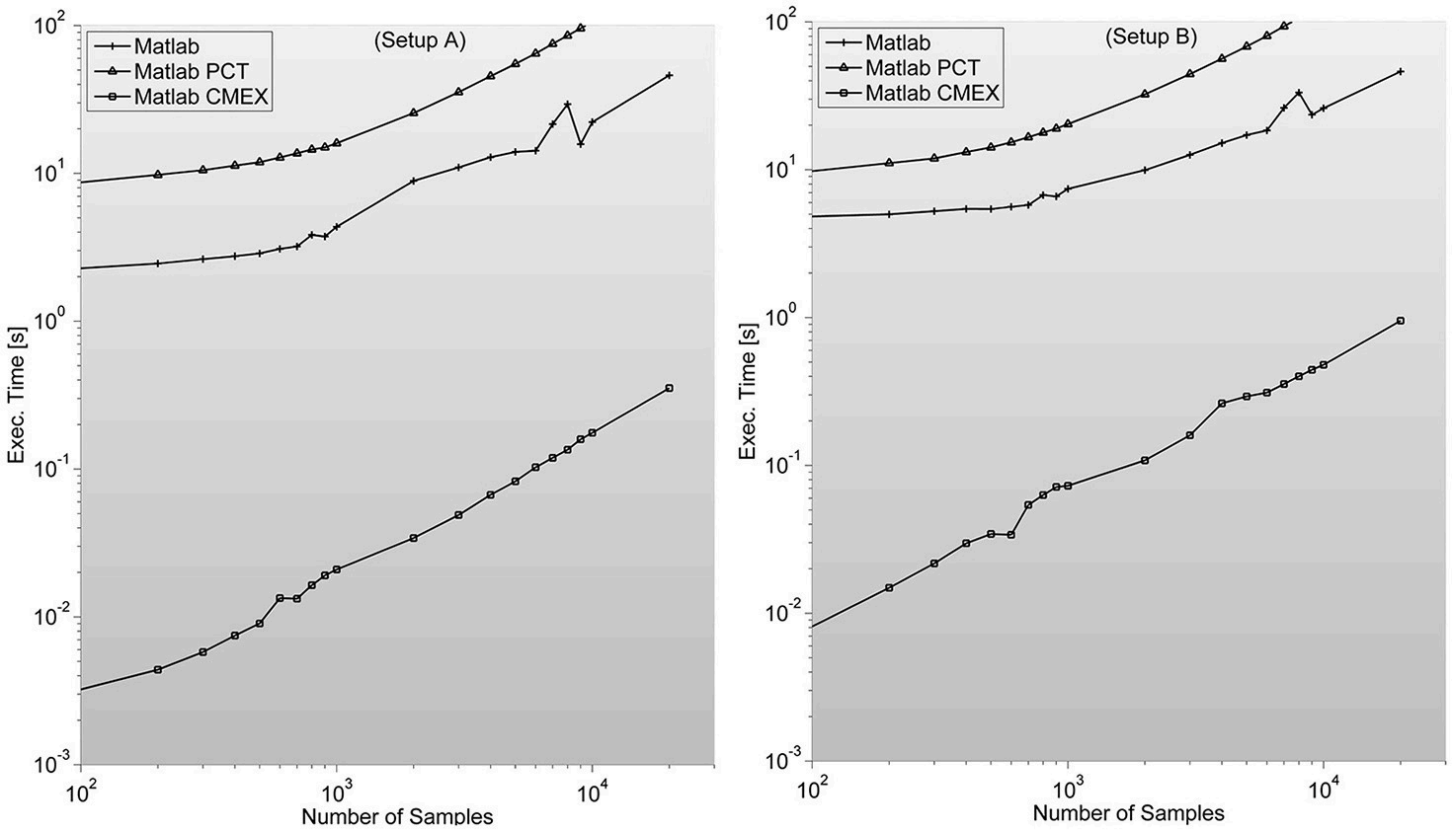
**Illustrates execution times (in seconds and measured with tic;toc; Matlab's built-in functions) of Phase Synchronization indices computation for a 128 sensor setup with variable number of samples**. Measures for different combinations of sensors (between 8 and 200) can be found in the Supplementary Material. It is surprising to find PCT-based parallelization an order of magnitude slower than straight-forward Matlab implementation.

As described in Mathworks' External Interface documentation, since MATLAB 7.3(R2006b) there is support for 64-bit indexing, enabling these routines to deal with variables with up to 2^48^-1 elements on 64-bit platforms. We tested two different C-mex implementations of the PS indices; one avoiding it and compiled with—*compatilbeArrayDims* flag, and another explicitly issuing 64-bit indexing by using *mwSize* and *mwIndex* types and compiling with –*largeArrayDims* flag. We found that the latter version is consistently around 30% slower than the former and therefore did not use it for subsequent developments (see Figure 2).

Regarding wPLI and ImC, the developed function follows Fieldtrip's implementation for wPLI. In it, Fourier spectra is first computed for each signal through a Welch's method with ~5 windows^12^ with 50% overlapping between them. Subsequently, cross-spectrum and Coherence are computed for every pair of input signals in order to define both indices.

### Zero-phase distortion filtering

Although slightly off-topic in relation with the theory of FC and network analysis, in this work we have included an optimized implementation of a filter. Typically, any neurophysiological signal is very susceptible to various sources of noise and as among the different strategies known in literature, we believe the most basic and widely used is to filter the signal. But most importantly, it is mandatory to narrow band-pass filter any signal from which PS is to be studied prior to the definition of the phase itself. We provide an implementation of zero-phase distortion FIR filtering routine, capable of removing unwanted frequency components form the signal and improving the signal-to-noise ratio of the brain signals, thereby improving the accuracy of FC measures (see Rulkov et al., 1995; Quian Quiroga et al., 2002; Chicharro and Andrzejak, 2009 for revisions on the behavior of different FC measures under controlled noise). Therefore, as in most cases it is advisable to filter any neurophysiological time-series, and in some cases it is required, we expect to help increase the speed of FC calculations including this version of a zero-phase distortion filtering function.

**Figure 3:**
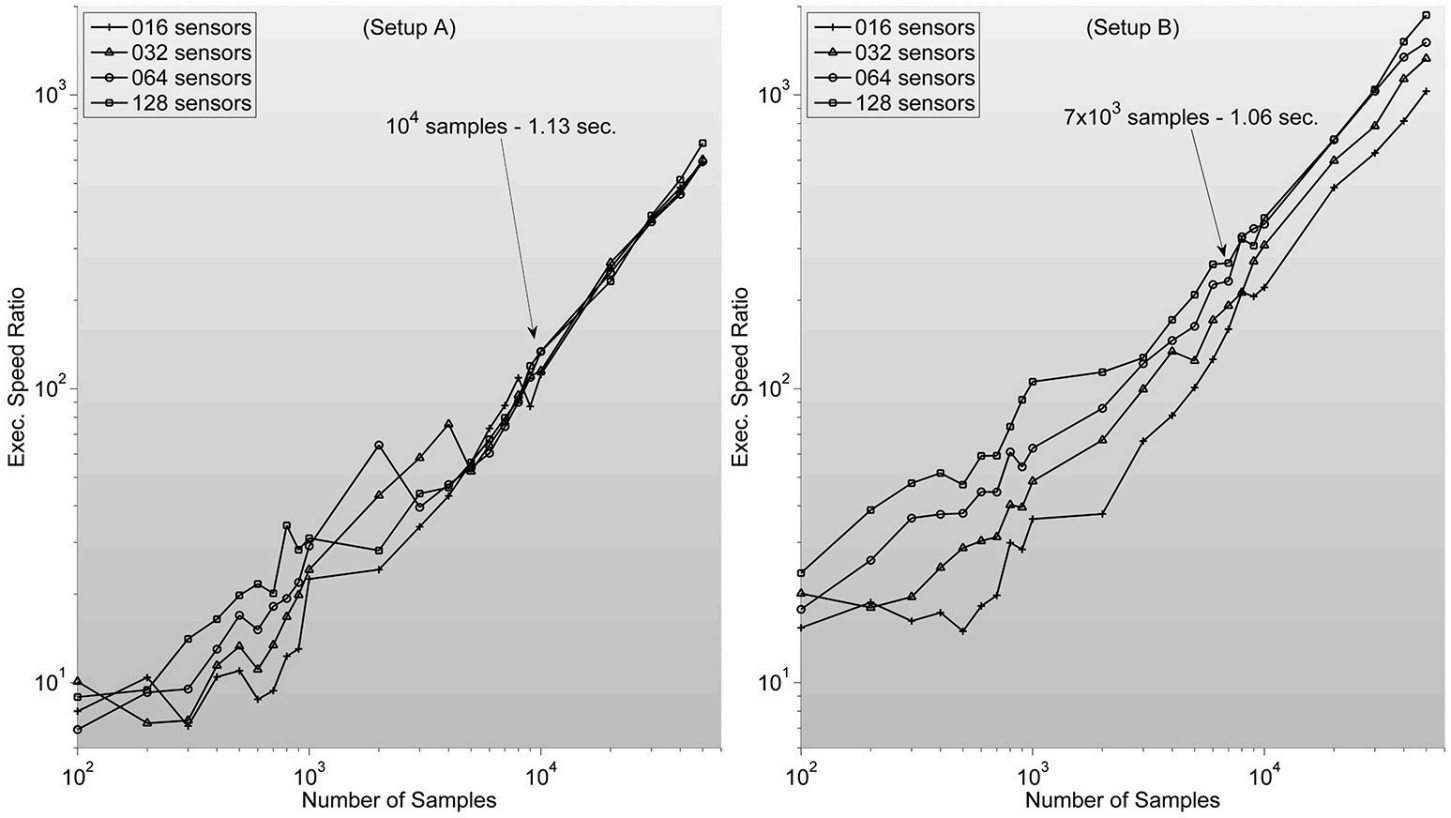
**Shows measures of speed-up ratio improvement of our implementation of zero-phase distortion FIR filter function over Matlab's Digital Signal Processing toolbox filtfilt function for different numbers of channels and samples**. Filter dimension is fixed as 1/5 sample length. Note how, surprisingly, improvement ratio is higher in setup B. Text description in each graph specifies Matlab's filtfilt function execution time for 128 sensors. Showing how, as expected, execution times are lower in setup A. Note also how the speed-up ratios are very similar to the expected outcome of 10^2^–10^3^, considering the stated theoretical analysis.

Let us review some key aspects of time-series filtering which are directly related to our implementation. Filtering can operate in different domains: in time domain, filtering can be understood as a convolution; in frequency domain, filtering can be understood as an array multiplication. This equivalence, generally known as the *circular convolution theorem*, allows avoiding a discrete convolution operation which requires $O\left(N_{samples}^{2}\right)$ operations by instead implementing two FFT *O*(*N*_*samples*_*logN*_*samples*_) and an array multiplication *O*(*N*_*samples*_), allowing a drastic reduction in execution times. As the filter needs to be applied in both forward and inverse directions in order to avoid phase distortion, by filtering in the frequency domain we attain a theoretical increase in computational efficiency by a factor of

(27) $$\begin{array}{l} O\;(\frac{2N_{samples}}{2{\mathrm{logN}}_{samples}+1}) \end{array}$$

which ranges between 10^2^ and 10^4^ for typical sample lengths (see Figure 3).

The development of our implementation has been closely guided by Matlab's Digital Signal Processing toolbox *filtfilt* function, in order to avoid introducing unnecessary confusion in an already well-established processing stage. Thus, the output is the same as the one obtained through that function^13^.

The filter is specified through its numerator array and it is transformed into the frequency domain. Prior to the filtering, the data are mirror padded at both sides by an amount of samples given by the filter dimension and the filter is zero padded to match subsequent length, all in order to reduce border effects. Filtering is then carried out efficiently in the frequency domain after applying the FFT to both the padded data and the filter itself (see Table 1 for different execution times).

**Table 1:** **Shows execution times measured with tic;toc; Matlab built-in functions, for zero-phase distortion FIR filtering while processing random time series of 2 × 10^**3**^ samples for two different configurations of 64 and 128 channels**.

	**No. of channels**	**Times**		**Speed-up**
		**Matlab's filtfilt**	**C-mex Impl**.	
Setup A	64	37 ms	0.15 ms	64 ×
	128	54 ms	0.17 ms	28 ×
Setup B	64	71 ms	0.83 ms	86 ×
	128	148 ms	1.3 ms	114 ×

*Last column illustrates speed-up ratio between Matlab version and this C-mex version*.

### MI

As previously discussed, there are different implementations of MI, most of them based on the naïve approach described above, that involves the estimation of probabilities (marginal and joint ones) using binning strategies. However, the approach based on k-nearest neighbors statistics as described in Kraskov et al. (2004) and recently reviewed in Kinney and Atwal (2014) is arguably one of the most suitable ones for large experimental datasets. Thus, we focused our attention, as starting points, on two efficient open-source implementations of this strategy, already in C++.

#### Currently available versions

##### TIM

This is a cross-platform open-source C++ library for the estimation of information-theory measures from continuous-valued time series^14^.

##### MILCA

This is the implementation of the authors of ref. (Kraskov et al., 2004)^15^ in C++ code. This turned out to be the fastest and most accurate one, and as we describe here, we therefore used it as a starting point for our development. We compare our implementation against it.

#### Our implementation

This MI implementation is based on a free program distributed in relation to former work (Kraskov et al., 2004), which is freely distributed within the MILCA toolbox. A Matlab version of their function for calculating the MI can be found in MILCA, being in fact a wrapper function that calls a C++ function outside Matlab's environment. This implementation will be alluded here as Matlab MILCA MI version.

One problem we identified in the Matlab version of MILCAs MI is that the interface between both environments (C++ program and Matlab) is a text file, thus making communication extremely inefficient. Our two main contributions to MILCA's implementation are to make it compatible with Windows Visual Studio compiler, and most importantly to change how the function receives data, making use of Matlab's MEX interface and being able to directly access Matlab's variables. We finally added multithread compatibility to our implementation, which substantially increased computation efficiency (see Table 2 for execution times for different implementations).

**Table 2:** **Shows execution times for different implementations of each Functional Connectivity index, while processing random time-series of 10^**3**^ samples for two different configurations of 32 and 64 channels**.

	**No. of channels**	**Execution time [S]**			**Speed-up**
		**Matlab**	**Matlab PCT**	**C-mex**	
**PHASE SYNCH**.					
Setup A	32	268 × 10^−3^	1.8	2 × 10^−3^	134 ×
	64	1	5	5 × 10^−3^	208 ×
Setup B	32	455 × 10^−3^	2.2	5 × 10^−3^	87 ×
	64	2	6	12 × 10^−3^	152 ×
**MUTUAL INFORMATION**					
Setup A	32	127	18	858 × 10^−3^	148 ×
	64	505	59	2.4	210 ×
Setup B	32	144	36	2.2	65 ×
	64	515	138	8.2	63 ×
**GENERALIZED SYNCH**					
Setup A	32	642	94	469 × 10^−3^	1368 ×
	64	2.4 × 10^3^	340	1.2	2026 ×
Setup B	32	593	178	991 × 10^−3^	598 ×
	64	2.4 × 10^3^	628	2.5	975 ×

*Times are expressed in seconds and measured with tic;toc; Matlab's built-in functions. Matlab versions are parallelized with Mathworks' Parallel Computing Toolbox (PCT). A wider perspective on the influence of PCT in terms of efficiency is drawn here. When computing Phase Synchronization, the computation is so fast that deployment of parallel executions through PCT appears to be slower than normal implementation. When the task to be parallelized lasts long enough, as in the case of Mutual Information and Generalized Synchronization indices, PCT demonstrates a very efficient tool to improve computational efficiency, as there are very few modifications in the codes that stand beneath first and second columns of results. Last column illustrates speed-up ratio between C-mex implementation and Matlab column*.

In order to allow the calculation of any of the two normalized versions of the MI (by or by (14) some other way), our implementation estimates not only (13) but also the individual entropies of each of the signals considered. This way we give users the possibility of implementing a MI normalization scheme to their liking.

**Figure 4:**
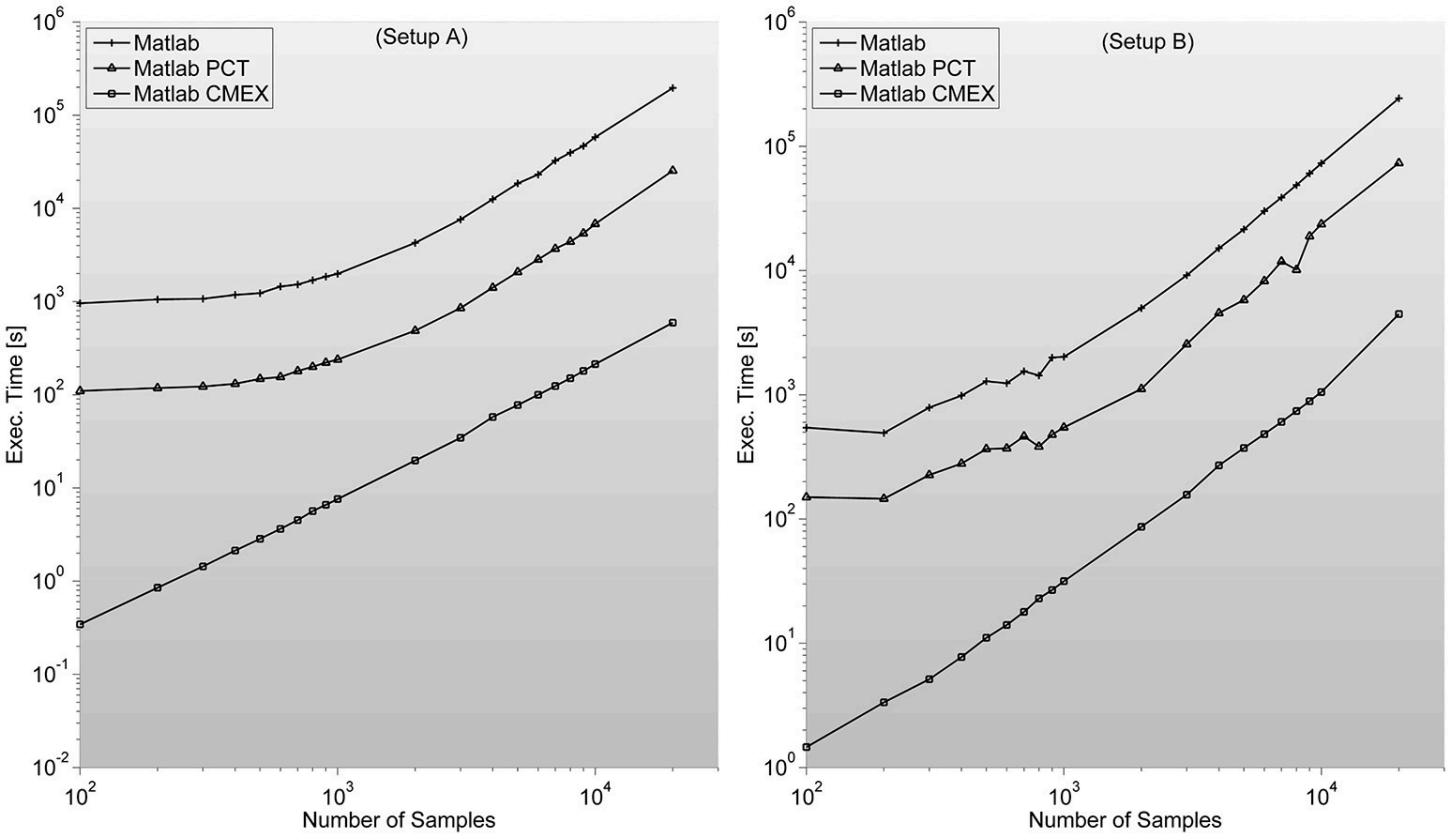
**shows the MI program execution times (in seconds) for a 128 sensor setup with variable number of samples**. As opposed to the case of PS, the MI parallelization through Mathworks Parallel Computing Toolbox is faster than straight Matlab execution, and both around two orders of magnitude slower than C-mex version.

It is worth noting that we also tested the feasibility of including in the platform the abovementioned MIC index (Reshef et al., 2011; Kinney and Atwal, 2014), which is normalized per definition and allegedly presents also very good statistical properties as an associated measure. Yet, on the one hand, the latest results seem to suggest that MIC is not better than Kraskov's MI implementation (Kinney and Atwal, 2014). On the other hand, even the fastest implementations of MIC (Tang et al., 2014) are orders of magnitude slower than the MI implementation we have obtained, which render this former index as unsuitable for real-time estimations of FC patterns (see Figure 4 for MI program execution times).

### GS index S, H, M, and L

#### Currently available versions

As far as we are aware, there are only two open-source implementations of the GS indices considered. Both Matlab based implementations:

One is included in the very elegant work of Chicharro and Andrzejak (2009) and freely distributed as online material for that publication. All S, H, M, and L indices are computed with an additional closely related N index described in Quian Quiroga et al. (2002). This implementation is not optimized for parallel implementation although it can be parallelized through Mathwork's Parallel Computational Toolbox.

##### HERMES^16^

A Matlab toolbox with a very complete set of indices and a convenient user interface freely distributed for the assessment of FC in EEG and MEG data (Niso et al., 2013).

None of them are optimized for performance and neither takes advantage of the existence of multicore multi-CPU.

#### Our implementation

Our implementation does not follow any of the previous Matlab based implementations. Instead, we developed our version in standard C (multithreaded through OpenMP) following the algorithms described in Chicharro and Andrzejak (2009) and references therein, with the only difference of using single-precision floating point representation instead of double precision. We then tested execution times between our version and previous Matlab based implementations.

All S, H, M, and L indices share the need to populate a reconstructed state space which instead of building sequentially, we manage to build implicitly while distances between every pair of reconstructed states are being accounted for. Besides, considering situations where the reconstructed embedded space is very populated, if the user considers it optimal to subsample the embedded reconstructed system, our version is capable of such subsampling, achieving a speed-up directly proportional to this subsampling.

Once distances are computed and stored, the algorithm calculates minimum distances. S, H, and M, could be computed even faster than in the present work because there are constrained by L, which considers ranked statistics and the ranking procedure, entails a first step consisting in the sorting of all the distances. This procedure grants this index a more reliable estimation of the *directionality* of the interaction, which, is also normalized between 0 and 1 for independent (resp. identical) signals but, on the other hand, it is precisely the sorting which makes this index the most computationally demanding of all the GS indices described here. For simplicity we present here a version that computes all four indices at the same time, including L, which is therefore the limiting factor in terms of computational efficiency in our implementation^17^.

**Figure 5:**
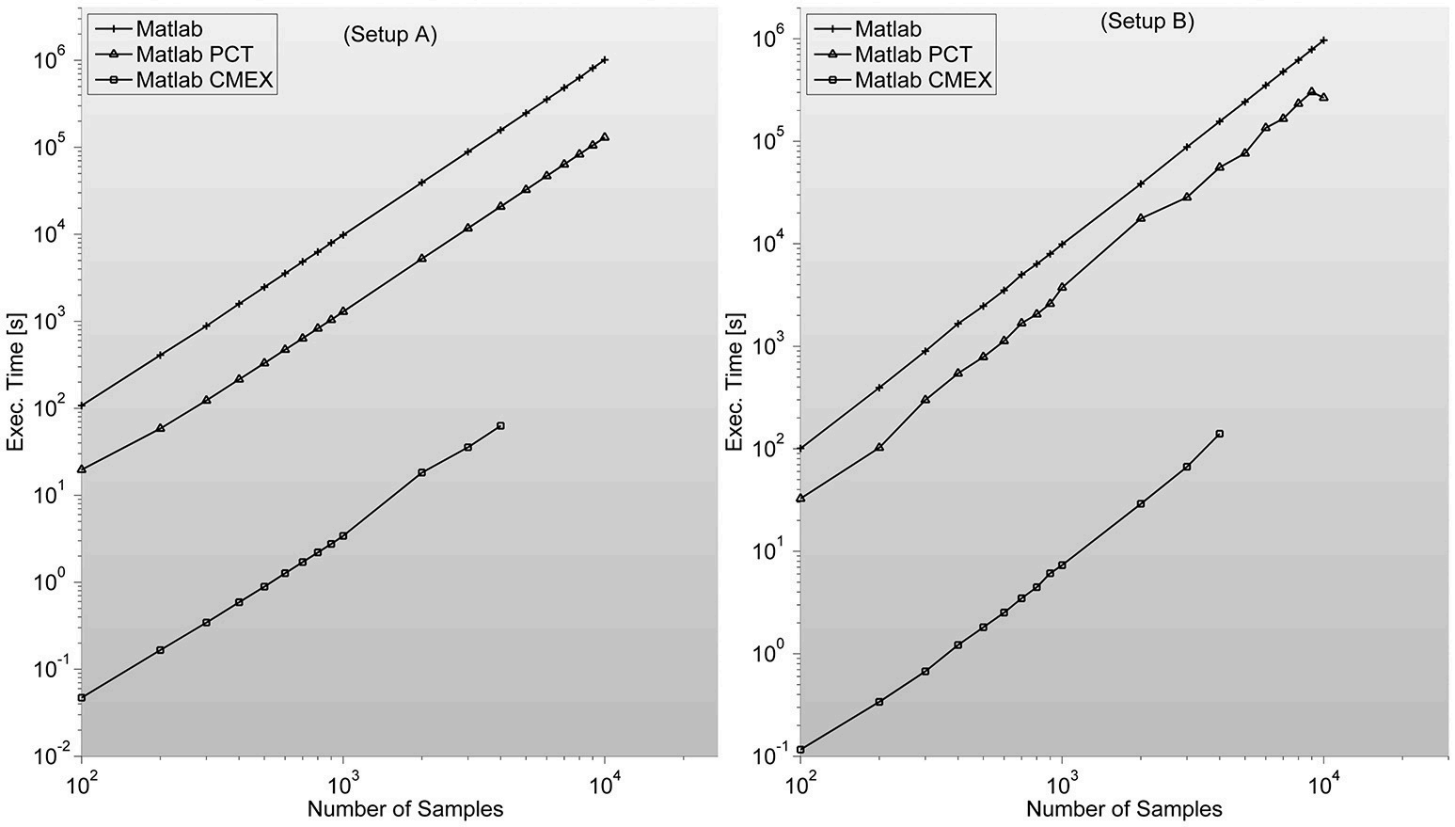
**Shows GS program execution times (in seconds) for a 128 sensor setup with variable number of samples**. It is in this case where C-mex implementation speeds-up over both Matlab based versions (straight Matlab and parallelized through Mathworks' Parallel Computing Toolbox) is most remarkable. This algorithm is by far the most demanding in terms of memory, as distances between reconstructed states have to be characterized pairwise. Lack of memory starts to be a problem in both setups when dealing with thousands of samples per sensor for the C-mex implementation. An easy solution is to segment the computation of the FC matrix in several sub-networks.

In certain situations, as it can clearly be seen in Figure 5, when the input matrix is big enough (thousands of samples in setup A and B) this can lead to out-of-memory problems as is the case of GS. The problem is that by construction, all implementations are prepared to accept an input matrix with the complete set of channels. This problem can be easily solved by calling the function by pairs of sensors, decreasing efficiency but allowing for much bigger sample lengths.

In order to help predict if a specific number of samples and channels might fit into a known setup, during its execution both floating-point and integer arithmetic take place.

The maximum amount of main memory used will be $N_{sensors}\times \left(2N_{samples}^{2}+\left(k+1\right)N_{samples}\right)\times SPFP$ for the floating point computations and $\left(2N_{samples}^{2}+N_{samples}\right)\times N_{sensors}\;\times INT$ for integer operations.^18^

### Complex networks indices

When a network has been characterized through FC, its adjacency matrix is populated with real-valued weights. Thus, we have implemented some complex networks indices for weighted networks, with or without directions depending on the FC index measured. Parallelization of network indices calculation is hard. Therefore, in this case only two versions of each index will be compared.

#### Currently available versions

Multiple network analysis software packages are freely available on the web. Among these packages, some concentrate on calculating measures for large networks, while others are visualization software. We focus here on command-line environments like Matlab.

Brain Connectivity Toolbox (BCT; Rubinov and Sporns, 2010). It is probably the most well-known set of functions to compute network theoretic measures. We are aware that BCT already includes a C++ implementation of most of the routines; however, as stated in their user manual, there is currently no efficient interface between BCT and Matlab's environment. We will try to fill this gap with an original implementation of a subset of those indices included in BCT.

#### Our implementation

In some cases, our implementation of brain connectivity measures follow very closely the implementation described and implemented in BCT. We had doubts while deciding whether to implement weighted or binary and directed or undirected network measures; we decided to concentrate on weighted adjacency matrices (both directed and undirected) because it seems more related to the type of networks usually created when working with FC-based networks.

**Figure 6:**
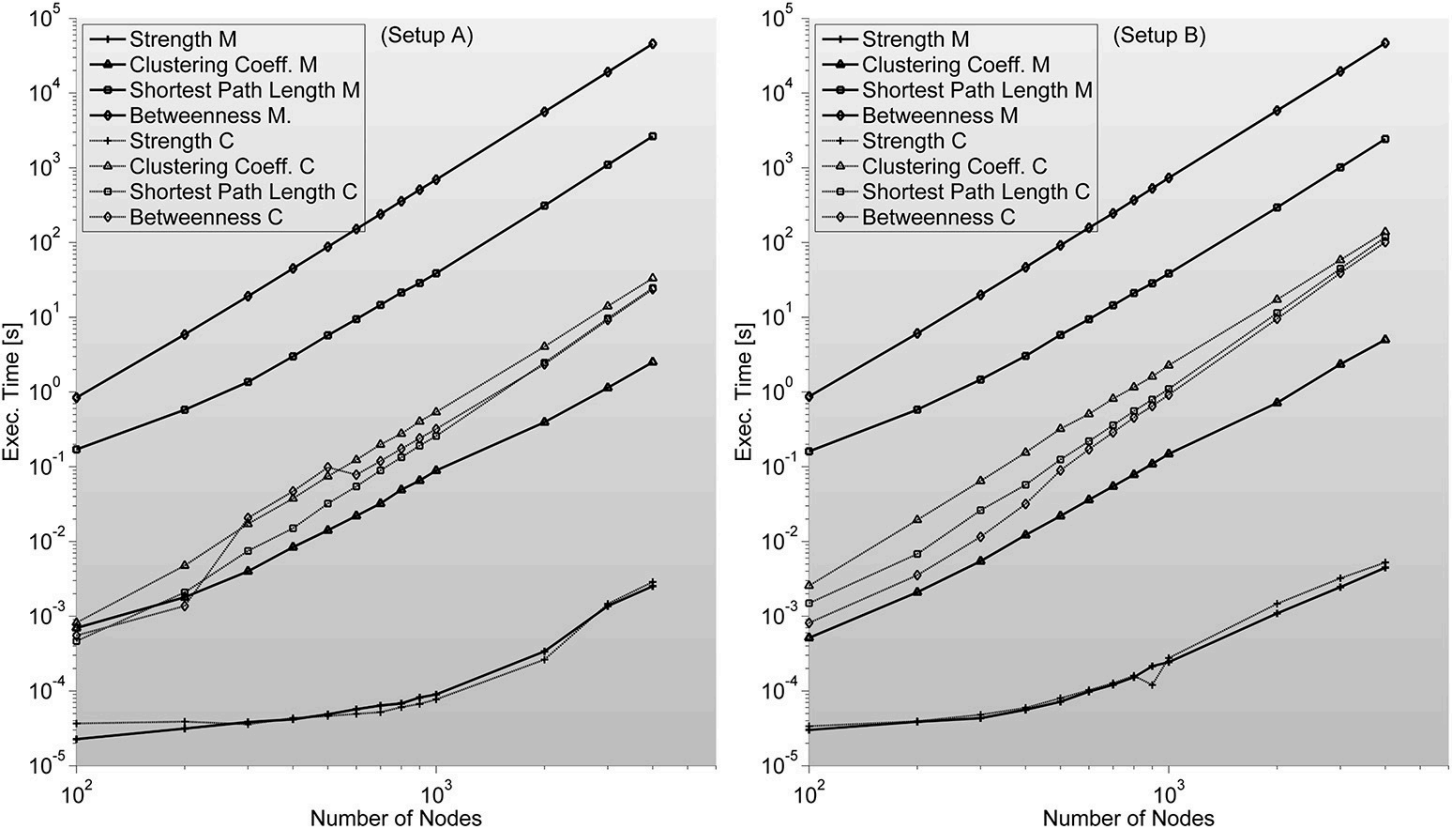
**Shows complex network indices execution times (in seconds) for both the original Matlab implementation (based on Brain Connectivity Toolbox) and C-mex implementations presented in this work**. We consider different number of nodes ranging from 100 up to 4000. An interesting outlook is described here; Strength index takes more or less the same to compute, even though C-mex version is more difficult to develop and maintain, and each compilation is restricted to an operating system. Clustering Coefficient appears to be much slower in the C-mex version; we believe this is related to the extensive use of the pow function (critically optimized for matrix calculations in the Matlab environment). Shortest Path Length and Betweenness are the most complex algorithms developed though the effort seems fruitful as C-mex implementations are between 2 and 3 orders of magnitude faster.

*Strength—*S is trivial to implement. It needs no additional memory to be reserved (apart from inputs and outputs) and can be implemented by a cumulative sum algorithm. We are certain this index should be included in this work as a representative example of situations when probable gain of C-mex implantation of an algorithm is unlikely. As it can be seen in Figure 6, C-mex implementation is dozens of times slower than Matlab's implementation. Through profiling, we are sure that C-mex implementation is dragged by the extensive use of *pow* function and as internally Matlab uses Intel's Math Kernel Library Basic Linear Algebra Subroutines (BLAS) and Linear Algebra Package (LAPACK) when running on Intel processors (which is the case throughout this work), the probable cause for such large efficiency gap is the optimization performed by these packages of the matrix-oriented *pow* function.

*Clustering coefficient*—C implementation closely follows the definitions of Rubinov and Sporns (2010). As a first attempt we implemented the definition of clustering coefficient as of Watts and Strogatz (1998) but that resulted in a very slow version. We then implemented the version proposed in Rubinov and Sporns (2010) with a much more efficient execution. Memory requirement is limited to $\left({N_{nodes}}^{2}+N_{nodes}\right)\times DPFP+N_{nodes}\times INT$ in our implementation.

Regarding *shortest path length*—L, we implement a new approach based on its definition. For each node, we find the shortest path to other nodes in the network applying Dijkstra's algorithm for weighted graphs. Following both (Boccaletti et al., 2006; Rubinov and Sporns, 2010) we set the definition of the length of each link as the inverse value of the weight—FC value, and replicate BCT results.

In terms of memory usage, L uses a set of backup registered arrays, and therefore an amount of memory directly related to the number of processors (logical cores) dedicated for its calculation. Thus, free memory must be kept above $N_{processors}\times \left(N_{nodes}\times \left(2\times Addr+INT\right)+N_{nodes}^{2}SPFP+N_{nodes}^{2}INT\right)$when calling the function.

Lastly, betweenness Centrality—B, which is considered an essential measure in many other areas in science, was implemented here following Brandes implementation (Brandes, 2001). It ranks network nodes highlighting their position in the network by solving the single-source shortest-paths problem, with no recursion in the implementation, assuming the graph as undirected and weighted. We implement Dijkstra's algorithm as it is a very efficient solution for finding shortest paths between nodes in a network prior to finding B. While searching for paths instead of a Fibonacci heap, we implement a linked-list of ordered paths avoiding the need for sorting which usually leads to this algorithms poor scalability. It is also important to note that the network matrix must have zeroed the main diagonal. Auto links are not accepted in this implementation. The maximum memory usage by this function is given by

(28) $$\begin{array}{l} N_{processors}\left(\left(N_{nodes}\times 2\times Addr+INT\right)+\left(N_{nodes}^{2}+N_{nodes}\right)INT\right)\\ +\ldots \;N_{nodes}^{2}\left(SPFP+INT\right)+N_{nodes}INT \end{array}$$

In our final version of this index, we have decided to keep L measures within B results as this adds very little overheads while allowing computing two indices at the same time.

### Information sharing statement

The majority of the source code described in this work is completely original, but not all. The MI routines are modified versions of Mutual Information Least-dependent Component Analysis (MILCA) which is free software distributed under General Public License (GPLv2) license. All Generalized Synchronization related code, Zero-Phase distortion filtering related code and Phase Synchronization related code are original, however the last two make use of FFTW precompiled libraries, which are also free software distributed under GPL. All the source code and executable binaries included in this work, are distributed under GNU GPLv3 at http://juangpc.github.io/FastFC/.

## Discussion

The aim of this work is to share an efficient implementation of a set of tools for estimating some well-known FC and graph-theoretic network measures and to give a self-sustained methodological review of these indices, describing some well-known previous open-source implementations. We have developed four families of measures totalling 10 different indices and a filtering function, all directly available from Matlab's *command line interface*. Which, given the ubiquity of Matlab in science and particularly in neuroscience, we hope renders them beneficial to FC and complex brain networks related studies by significantly decreasing computational times, while controlling the friction inherent in adopting new functions.

By *real-time* estimation we mean that the time necessary to calculate the network patterns is more or less the same as the time necessary to record the data. In fact, for a typical sampling frequency of 500 Hz, the FC network patterns of 2 s of data (10^3^ samples) from a 32-channel EEG requires less than a second of computational time each, enabling the possibility to interact directly with the subject based on its FC properties, while the task is on-going within a neuro-feedback scheme.

We have achieved a speed-up ratio ranging from the hundreds in the case of PS indices (which are the fastest ones), low and high hundreds in case of the MI and fluctuating from hundreds to thousands in the case of GS indices (the slowest ones). But most importantly, the execution times obtained in all cases allow the most efficient estimation of the network patterns, drastically reducing computational times under all circumstances and enabling new setups that were not feasible before. Regarding PS indices, which are calculated extremely fast, it is even possible to obtain a real-time estimation of the network patterns for the whole frequency range of an EEG/MEG.

Few studies dealing with real-time—or close to real-time—FC based measures have been published so far. However, as described before, these studies suffer from the need to lighten computational weight usually by restricting to a series of ROIs or reducing the number of channels. Either solution disables proper network analysis characterization of the whole brain. But these results suggest that both FC and *effective connectivity* of brain networks should be taken into consideration as an effective dimensionality reduction technique for BCI and neuro-feedback setups. In Daly et al. (2012) it has been shown how inter-regional connectivity can positively impact BCI accuracy and speed, which can only improve if taking into consideration whole-head high-density FC estimation. Moreover, an example of its application to MEG data in epilepsy, this index has been recently used to assess the outcome of surgery in a follow up study of lesional epilepsy patients (van Dellen et al., 2014).

In terms of programming, we have tried to avoid repetition of calculations; reuse and storing prevailing over recalculating. We kept all the implementations as single-file-programs, in order to help further implementations and facilitate other toolboxes adding this implementation to theirs. The development has maintained portability as a main priority by only making use of the standard C-mex library or libraries supported under different environments, thus making the development portable and platform independent. Although, one major inconvenience in any C-mex based parallelization is that each compilation is restricted to its operating system. Further developments oriented toward embedded devices or heterogeneous computing devices might use initiatives like OpenCL^19^, CUDA^20^, OpenGL^21^, OpenACC^22^, or OpenMP for GPU based computing, we consider C to be the most valid interaction language between all of them.

We have included some graph-theoretical network measures in this work that actually increase computational times in relation to most well-known Matlab implementation. Clustering Coefficient, for instance can be hundreds of times slower than BCT's Matlab version. We have decided to include these developments in case they help future implementations in specific computing devices. Furthermore, we have developed different versions of each implementation, measuring execution times and increasing knowledge on how much improvement shall be expected from further or analogue implementations. Thus, a significant amount of effort has been devoted to describe the interesting landscape of results obtained. We hope this experience can aid any researcher who is considering the effort of improving computational efficiency of an algorithm within Matlab's environment with PCT or through C-mex implementation and parallelization; our results should allow for reasoned or educated predictions of improvements.

In terms of hardware influences, differences in performance between setups are constant and consistent with the expected outcome of setup A being faster than B. For small sample sizes though, lack of differences can be caused by differences in memory bandwidth due to error-correcting code memory being used in setup A, and CPU frequency. It is difficult to be sure.

In general terms, the lesson is that differences in execution times caused by improving hardware setup up are only significant when the amount of data is big or when using the C-mex implementation. Regarding PCT, we feel that for the purpose of a general speeding up, PCT takes only a very tiny fraction of the development time in contrast to C-mex solution. And its results very much depend on regular Matlab execution times. Apparently, there is a fixed time penalty for launching the workers in the parallel pool and when each worker's execution times are measured in milliseconds, it does not make sense to use PCT parallelization. In terms of task complexity while developing the code, under a worst case scenario C-mex will increase the complexity of the task by an order of magnitude and might decrease computational times by two or three times that factor. But if Matlab's implementation is very condensed and relies on few function calls, C-mex's implementation can be much slower than Matlab's. Matlab has reported to use Intel's Math Kernel library optimized for matrix calculations; even considering inefficiencies of scripting languages, when only called a few times, Matlab's functions prove to be well-optimized. Surprisingly, a counter example is *filtfilt* function. Interestingly Matlab itself uses FFTW library for FFT calculations, even though this function's efficiency can be radically increased with our C-mex frequency domain based parallel implementation. Furthermore, for our examples we have specified mode 2 optimization for FT computation, which corresponds to FFTW_ESTIMATE optimization mode within FFTW's *vocabulary*. Therefore, results could only improve if using more optimized procedures, as described in FFTW's user guide. A matter of much interest and controversy for many years (Moler, 1995), a parallel Matlab implementation has not yet been accomplished. Since Matlab 2008a version, some algebra and numeric functions such as *fft* in Matlab are multithread. But still we consider much of C-mex improvement is due to the use of OpenMP thread-based parallelization throughout the computation.

Part of the future developments that rise from this work consist on investigating whether (and how much) GPU based computations might further increase computational efficiency. Development of GPU processors driven by the game consumer industry has produced outstanding achievements and a change in the way scientists see GPU computation (Nickolls and Dally, 2010). Creating an incessant stream of successfully translated algorithms and applications into GPU makes us believe it is worth studying the advantages and disadvantages of porting FC and network indices to GPU oriented implementations (see Rosales et al., 2015; Wollstadt et al., 2014 for a close related example). Thus, as a community we shall make sure this field takes full advantage of heterogeneous multicore CPU/GPU architectures. In this context, we expect that further implementations based on CUDA or other standards like OpenGL can benefit from this work extracting full capabilities of these devices.

Overall, we hope this work can assist neuroscientific experiments with stimulation protocols dependent on FC and network theory related measures. As EEG is probably the most common neurophysiological technique, we concentrated on it with examples and use cases throughout the text, although this work and its conclusions can easily be adapted to other techniques.

## Author contributors

JG, EP: Substantial contributions to the conception and design of the work. JG, RB, EP: Contribution to the analysis and interpretation of the data. JG, RB, EP: Drafting and revision of the work. JG, RB, EP: Final approval of the version to be published.

### Conflict of interest statement

The authors declare that the research was conducted in the absence of any commercial or financial relationships that could be construed as a potential conflict of interest.

## Footnotes

^1^MATLAB, The MathWorks, Inc., Natick, Massachusetts, United States.

^2^http://www.mathworks.com/help/matlab/programming-interfaces-for-c-c-fortran-com.html

^3^http://openmp.org/wp

^4^Brain Connectivity Toolbox. https://sites.google.com/site/bctnet/

^5^different from *walks*, which are sequences of linked nodes that may visit a single node more than once.

^6^http://www.fftw.org/benchfft

^7^Microsoft Visual Studio supports up to OpenMP v2.0. See https://msdn.microsoft.com/en-us/library/tt15eb9t(v=vs.100).aspx

^8^DAMOCO. http://www.stat.physik.uni-potsdam.de/~mros/damoco2.html

^9^FieldTrip. http://www.fieldtriptoolbox.org

^10^EEGLab. http://sccn.ucsd.edu/eeglab

^11^SPFP is the number of Bytes a single-precision floating point number shatters, typically, 4 (Bytes).

^12^The length of each signal is divided by 4.5 and the result is transformed into an integer number of windows.

^13^With a slight difference derived from implementing single-precision floating-point instead of double precision floating-point arithmetic.

^14^TIM. http://www.cs.tut.fi/~timhome/tim/tim.htm

^15^MILCA.http://www.ucl.ac.uk/ion/departments/sobell/Research/RLemon/MILCA/MILCA

^16^http://hermes.ctb.upm.es/

^17^Should the reader want to compute S, H, or M but not L, despite being less robust in noisy situations, a more efficient implementation of our program which computes only S, H, and M without the need for sorting is available in the repository.

^18^SPFP is the number of Bytes a single-precision floating point number shatters, typically, 4 Bytes. And analogously INT represents the length of an integer value, which is usually 4 Bytes as well.

^19^www.khronos.org/opencl/

^20^www.nvidia.com/object/cuda_home_new.html

^21^www.khronos.org/opencl/

^22^www.openacc-standard.org/

## References

[B1] Andrzejak R. G. Kraskov A. Stögbauer H. Stogbauer H. Mormann F. Kreuz T. (2003). Bivariate surrogate techniques: necessity, strengths, and caveats. Phys. Rev. E. 68, 1–22. 10.1103/PhysRevE.68.066202 14754292

[B2] Arnhold J. Grassberger P. Lehnertz K. Elger C. E. (1999). A robust method for detecting interdependences: application to intracranially recorded EEG. Phys. D Nonlinear. Phenom. 134, 419–430. 10.1016/S0167-2789(99)00140-2

[B3] Benjamini Y. Hochberg Y. (1995). Controlling the false discovery rate: a practical and powerful approach to multiple testing. J. R. Stat. Soc. Ser. B 57, 289–300.

[B4] Bialonski S. (2012). Inferring complex networks from time series of dynamical systems: pitfalls, misinterpretations, and possible solutions. Arxiv Preprint arXiv: 1208.0800.

[B5] Boccaletti S. Latora V. Moreno Y. Chavez M. Hwang D. U. (2006). Complex networks: structure and dynamics. Phys. Rep. 424, 175–308. 10.1016/j.physrep.2005.10.009

[B6] Bonita J. D. Ambolode L. C. C. II Rosenberg B. M. Cellucci C. J. Watanabe T. A. Rapp P. E.. (2014). Time domain measures of inter-channel EEG correlations: a comparison of linear, nonparametric and nonlinear measures. Cogn. Neurodyn. 8, 1–15. 10.1007/s11571-013-9267-8 24465281 PMC3890093

[B7] Brandes U. (2001). A faster algorithm for betweenness centrality. J. Math. Sociol. 25, 163–177. 10.1080/0022250X.2001.9990249

[B8] Bullmore E. Sporns O. (2009). Complex brain networks: graph theoretical analysis of structural and functional systems. Nat. Rev. Neurosci. 10, 186–198. 10.1038/nrn2575 19190637

[B9] Chella F. Pizzella V. Zappasodi F. Marzetti L. (2016). Impact of the reference choice on scalp EEG connectivity estimation. J. Neural Eng. 13:36016. 10.1088/1741-2560/13/3/036016 27138114

[B10] Chicharro D. Andrzejak R. G. (2009). Reliable detection of directional couplings using rank statistics. Phys. Rev. E 80, 26217. 10.1103/PhysRevE.80.026217 19792241

[B11] Christodoulakis M. Hadjipapas A. Papathanasiou E. S. Anastasiadou M. Papacostas S. S. Mitsis G. D. (2014). On the effect of volume conduction on graph theoretic measures of brain networks in Epilepsy. Neuromethods 91, 1–28. 10.1007/7657_2013_65

[B12] Cohen M. X. (2015). Effects of time lag and frequency matching on phase-based connectivity. J. Neurosci. Methods 250, 137–146. 10.1016/j.jneumeth.2014.09.005 25234308

[B13] Dagum L. Menon R. (1998). OpenMP: an industry standard API for shared-memory programming. IEEE Comput. Sci. Eng. 5, 46–55. 10.1109/99.660313

[B14] Daly I. Nasuto S. J. Warwick K. (2012). Brain computer interface control via functional connectivity dynamics. Pattern Recogn. 45, 2123–2136. 10.1016/j.patcog.2011.04.034

[B15] DeFelipe J. (2010). From the connectome to the synaptome: an epic love story. Science 330, 1198–1201. 10.1126/science.1193378 21109663

[B16] Filmer H. L. Dux P. E. Mattingley J. B. (2014). Applications of transcranial direct current stimulation for understanding brain function. Trends Neurosci. 37, 742–753. 10.1016/j.tins.2014.08.003 25189102

[B17] Freeman L. C. (1979). Centrality in social networks conceptual clarification. Soc. Networks 1, 215–239.

[B18] Friston K. J. (2011). Functional and effective connectivity: a review. Brain Connect. 1, 13–36. 10.1089/brain.2011.0008 22432952

[B19] Hlavackova-Schindler K. Palus M. Vejmelka M. Bhattacharya J. (2007). Causality detection based on information-theoretic approaches in time series analysis. Phys. Rep. 441, 1–46. 10.1016/j.physrep.2006.12.004

[B20] Horwitz B. (2003). The elusive concept of brain connectivity. Neuroimage 19, 466–470. 10.1016/S1053-8119(03)00112-5 12814595

[B21] Hutchison R. M. Womelsdorf T. Allen E. A. Bandettini P. A. Calhoun V. D. Corbetta M.. (2013). Dynamic functional connectivity: promise, issues, and interpretations. Neuroimage 80, 360–378. 10.1016/j.neuroimage.2013.05.079 23707587 PMC3807588

[B22] Kantz H. Schreiber T. (2004). Nonlinear Time Series Analysis. Cambridge: Cambridge University Press.

[B23] Kinney J. B. Atwal G. S. (2014). Equitability, mutual information, and the maximal information coefficient. Proc. Natl. Acad. Sci. U.S.A. 111, 3354–3359. 10.1073/pnas.1309933111 24550517 PMC3948249

[B24] Kraskov A. Stögbauer H. Grassberger P. (2004). Estimating mutual information. Phys. Rev. E 69:66138. 10.1103/PhysRevE.69.066138 15244698

[B25] Kugiumtzis D. Kimiskidis V. (2015). Direct causal networks for the study of transcranial magnetic stimulation effects on focal epileptiform discharges. Int. J. Neural Syst. 25:1550006. 10.1142/S0129065715500069 25761527

[B26] Lachaux J. P. Rodriguez E. Martinerie J. Varela F. J. (1999). Measuring phase-synchrony in brain signals. Hum. Brain Mapp. 8, 194–208. 10619414 10.1002/(SICI)1097-0193(1999)8:4<194::AID-HBM4>3.0.CO;2-C PMC6873296

[B27] Mardia K. V. Jupp P. E. (2000). Directional Statistics. West Sussex: John Wiley & Sons.

[B28] Marzetti L. Nolte G. Perrucci M. G. Romani G. L. Del Gratta C. (2007). The use of standardized infinity reference in EEG coherency studies. Neuroimage 36, 48–63. 10.1016/j.neuroimage.2007.02.034 17418592

[B29] Medaglia J. D. Lynall M.-E. Bassett D. S. (2015). Cognitive network neuroscience. J. Cogn. Neurosci. 27, 1471–1491. 10.1162/jocn_a_00810 25803596 PMC4854276

[B30] Moler C. (1995). Why There Isn't a Parallel MATLAB. Cleve's Corner, Mathworks Newsletter.

[B31] Mormann F. Lehnertz K. David P. Elger C. E. (2000). Mean phase coherence as a measure for phase synchronization and its application to the EEG of epilepsy patients. Phys. D Nonlinear Phenom. 144, 358–369. 10.1016/S0167-2789(00)00087-7

[B32] Moxon K. A. Foffani G. (2015). Brain-machine interfaces beyond neuroprosthetics. Neuron 86, 55–67. 10.1016/j.neuron.2015.03.036 25856486

[B33] Nickolls J. Dally W. J. (2010). The GPU computing era. IEEE Micro. 30, 56–69. 10.1109/MM.2010.41

[B34] Niso G. Bruña R. Pereda E. Gutiérrez R. Bajo R. Maestu F.. (2013). HERMES: towards an integrated toolbox to characterize functional and effective brain connectivity. Neuroinformatics 11, 1–58. 10.1007/s12021-013-9186-1 23812847

[B35] Nolte G. Bai O. Wheaton L. Mari Z. Vorbach S. Hallett M. (2004). Identifying true brain interaction from EEG data using the imaginary part of coherency. Clin. Neurophysiol. 115, 2292–2307. 10.1016/j.clinph.2004.04.029 15351371

[B36] Pereda E. Quiroga R. Q. Bhattacharya J. (2005). Nonlinear multivariate analysis of neurophysiological signals. Prog. Neurobiol. 77, 1–37. 10.1016/j.pneurobio.2005.10.003 16289760

[B37] Pereda E. Rial R. Gamundi A. González J. (2001). Assessment of changing interdependencies between human electroencephalograms using nonlinear methods. Phys. D Nonlinear Phenom. 148, 147–58. 10.1016/S0167-2789(00)00190-1

[B38] Pompe B. Blidh P. Hoyer D. Eiselt M. (1998). Using mutual information to measure coupling in the cardiorespiratory system. IEEE Eng. Med. Biol. Mag. 17, 32–9. 10.1109/51.731318 9824759

[B39] Porz S. Kiel M. Lehnertz K. (2014). Can spurious indications for phase synchronization due to superimposed signals be avoided? Chaos 24, 033112. 10.1063/1.4890568 25273192

[B40] Quian Quiroga R. Kraskov A. Kreuz T. Grassberger P. Quiroga R. Q. Kraskov A.. (2002). Performance of different synchronization measures in real data: A case study on electroencephalographic signals. Phys. Rev. E 65:41903. 10.1103/PhysRevE.65.041903 12005869

[B41] Reshef D. N. Reshef Y. A. Finucane H. K. Grossman S. R. McVean G. Turnbaugh P. J.. (2011). Detecting novel associations in large data sets. Science 334, 1518–1524. 10.1126/science.1205438 22174245 PMC3325791

[B42] Romano M. C. Thiel M. Kurths J. Mergenthaler K. Engbert R. (2009). Hypothesis test for synchronization: twin surrogates revisited. Chaos 19, 015108. 10.1063/1.3072784 19335012

[B43] Rosales F. García-Dopico A. Bajo R. Nevado Á. (2015). An efficient implementation of the synchronization likelihood algorithm for functional connectivity. Neuroinformatics 13, 245–258. 10.1007/s12021-014-9251-4 25500965

[B44] Rosenblum M. G. Pikovsky A. S. Kurths J. (1996). Phase synchronization of chaotic oscillators. Phys. Rev. Lett. 76, 1804–1807. 10060525 10.1103/PhysRevLett.76.1804

[B45] Rubinov M. Sporns O. (2010). Complex network measures of brain connectivity: uses and interpretations. Neuroimage 52, 1059–1069. 10.1016/j.neuroimage.2009.10.003 19819337

[B46] Rulkov N. Sushchik M. Tsimring L. Abarbanel H. (1995). Generalized synchronization of chaos in directionally coupled chaotic systems. Phys. Rev. E 51, 980–994. 10.1103/PhysRevE.51.980 9962737

[B47] Schmitz A. (2000). Measuring statistical dependence and coupling of subsystems. Phys. Rev. E 62, 7508–7511. 11102120 10.1103/physreve.62.7508

[B48] Seth A. K. (2010). A MATLAB toolbox for Granger causal connectivity analysis. J. Neurosci. Methods 186, 262–273. 10.1016/j.jneumeth.2009.11.020 19961876

[B49] Shannon C. E. Weaver W. (1949). The Mathematical Theory of Information. Urbana, IL: University Press.

[B50] Stam C. J. (2005). Nonlinear dynamical analysis of EEG and MEG: review of an emerging field. Clin. Neurophysiol. 116, 2266–2301. 10.1016/j.clinph.2005.06.011 16115797

[B51] Stam C. J. Nolte G. Daffertshofer A. (2007). Phase lag index: assessment of functional connectivity from multi channel EEG and MEG with diminished bias from common sources. Hum. Brain Mapp. 28, 1178–1193. 10.1002/hbm.20346 17266107 PMC6871367

[B52] Sugihara G. May R. Ye H. Hsieh C. H. Deyle E. Fogarty M.. (2012). Detecting causality in complex ecosystems. Science 338, 496–500. 10.1126/science.1227079 22997134

[B53] Takens F. (1980). Detecting strange attractors in turbulence. Dyn. Syst. Turbul. 898, 366–381.

[B54] Tang D. Wang M. Zheng W. Wang H. (2014). RapidMic: rapid computation of the maximal information coefficient. Evol. Bioinform. Online 10, 11–16. 10.4137/EBO.S13121 24526831 PMC3921152

[B55] Tass P. Rosenblum M. G. Weule J. Kurths J. Pikovsky A. Volkmann J. (1998). Detection of n:m phase locking from noisy data: application to magnetoencephalography. Phys. Rev. Lett. 81, 3291–3294.

[B56] Thiel M. Romano M. C. Kurths J. Rolfs M. Kliegl R. (2006). Twin surrogates to test for complex synchronisation. Europhys. Lett. 75, 535–541. 10.1209/epl/i2006-10147-0

[B57] van Dellen E. Douw L. Hillebrand A. de Witt Hamer P. C. Baayen J. C. Heimans J. J.. (2014). Epilepsy surgery outcome and functional network alterations in longitudinal MEG: a minimum spanning tree analysis. Neuroimage 86, 354–363. 10.1016/j.neuroimage.2013.10.010 24128736

[B58] van Gerven M. Farquhar J. Schaefer R. Vlek R. Geuze J. Nijholt A.. (2009). The brain-computer interface cycle. J. Neural Eng. 6, 041001. 10.1088/1741-2560/6/4/041001 19622847

[B59] Vicente R. Gollo L. L. Mirasso C. R. Fischer I. Pipa G. (2008). Dynamical relaying can yield zero time lag neuronal synchrony despite long conduction delays. Proc. Natl. Acad. Sci. U.S.A. 105, 17157–17162. 10.1073/pnas.0809353105 18957544 PMC2575223

[B60] Vinck M. Oostenveld R. van Wingerden M. Battaglia F. Pennartz C. M. (2011). An improved index of phase-synchronization for electrophysiological data in the presence of volume-conduction, noise and sample-size bias. Neuroimage 55, 1548–1565. 10.1016/j.neuroimage.2011.01.055 21276857

[B61] Wang H. E. Bénar C. G. Quilichini P. P. Friston K. J. Jirsa V. K. Bernard C.. (2014). A systematic framework for functional connectivity measures. Front Neurosci. 8:405. 10.3389/fnins.2014.00405 25538556 PMC4260483

[B62] Watts D. J. Strogatz S. H. (1998). Collective dynamics of “small-world” networks. Nature 393, 440–442. 10.1038/30918 9623998

[B63] Wilkie D. (1983). Rayleigh test for randomness of circular data. Appl. Stutist. 32, 311–312.

[B64] Witten I. Frank E. Hall M. (2011). Data Mining : Practical Machine Learning Tools and Techniques Second Edition. Available onlie at: http://www.amazon.com/Data-Mining-Practical-Techniques-Management/dp/0120884070/ref=sr_1_2?s=books&ie=UTF8&qid=1366635595&sr=1-2&keywords=data+mining+practical+machine+learning+tools+and+techniques

[B65] Wollstadt P. Martínez-Zarzuela M. Vicente R. Díaz-Pernas F. J. Wibral M. (2014). Efficient transfer entropy analysis of non-stationary neural time series. PLoS ONE 9:e102833. 10.1371/journal.pone.0102833 25068489 PMC4113280

[B66] Zanin M. Sousa P. Papo D. Bajo R. García-Prieto J. del Pozo F.. (2012). Optimizing functional network representation of multivariate time series. Sci. Rep. 2:630. 10.1038/srep00630 22953051 PMC3433690

