# Supplementary material for: Efficient Computation of Functional Brain Networks: toward Real-Time Functional Connectivity
Source: Front Neuroinform. 2017 Feb 6;11:8. doi: 10.3389/fninf.2017.00008 (PMC5292573; doi:10.3389/fninf.2017.00008)
Supplement: Supplementary file 1 [file Presentation1.PDF]

Supplementary Material for

Real-time estimation of functional brain networks

Authors: Juan García-Prieto[1, 2,*], Ricardo Bajo [2,4], Ernesto Pereda[1, 2, 3]

[1] Laboratory of Electrical Engineering and Bioengineering, Dept. of Industrial Engineering, Universidad de La Laguna, Avda. Astrofísico Fco. Sanchez s/n 38206 Tenerife, Spain.

[2] Laboratory of Computational and Cognitive Neuroscience, Centre of Biomedical Technology, UPM, Madrid, Spain.

[3] Institute of Biomedical Technology (ITB-CIBICAN), Universidad de La Laguna, Tenerife, Spain

[4] Portucalense Institute of Neuropsychology (INPP), Universidade de Portucalense, Oporto, Portugal.

* To whom correspondence should be addressed: jgarcipr@ull.edu.es

Index

1. List of Figures 2
2. Software Configuration 4
3. Hardware Setup Description....4
   3.1. Setup A: 4
   3.2. Setup B:....4
4. Phase Synchronization Measures 5
5. Mutual Information 14
6. GS Indices 23
7. Network Measures....32
8. User Manual 35
   8.1. Who can use FastFC 35
   8.2. Installation 35
   8.3. Developers 35
   8.4. List of Functions 35
      8.4.1. Zero Phase Distortion Filter....36
      8.4.2. Phase Synchronization....36
      8.4.3. Mutual Information....37
      8.4.4. Generalized Synchronization....37
      8.4.5. Strength....37
      8.4.6. Clustering Coefficient....37
      8.4.7. Shortest Path Length....38
      8.4.8. Betweenness Centrality 38

1. List of Figures

Figure 1 shows execution times for Matlab code implementation of Phase Synchronization indices for setup A. The implementation is based on bsxfun function. 6

Figure 2 shows execution times for Matlab code implementation of Phase Synchronization indices for setup B. The implementation is based on bsxfun function. 7

Figure 3 shows execution times for Matlab code implementation of Phase Synchronization indices for setup A, parallelized through Parallel Computational Toolbox, using *parfor* structure. The implementation is based on bsxfun function. 8

Figure 4 shows execution times for Matlab code implementation of Phase Synchronization indices for setup B, parallelized through Parallel Computational Toolbox, using *parfor* structure. The implementation is based on bsxfun function. 9

Figure 5 shows execution times for c-mex developed C code of Phase Synchronization indices for setup A, parallelized through OpenMP. 10

Figure 6 shows execution times for c-mex developed C code of Phase Synchronization indices for setup A, parallelized through OpenMP. 11

Figure 7 shows execution times in setup A for different implementations of Phase Synchronization indices for a 64 sensor setup and different sample lengths. 12

Figure 8 shows execution times in setup B for different implementations of Phase Synchronization indices for a 64 sensor setup and different sample lengths. 13

Figure 9 shows execution times in setup A, for Matlab code implementation of MI function. This function was downloaded from MILCA's toolbox website, as a function called *MIhigherdim*. It is in fact a wrapper of a C++ program. 15

Figure 10 shows execution times in setup B, for Matlab code implementation of MI function. This function was downloaded from MILCA's toolbox website, as a function called *MIhigherdim*. It is in fact a wrapper of a C++ program. 16

Figure 11 shows execution times for setup A, for Matlab code implementation of MI function. This function was downloaded from MILCA's toolbox website, as a function called *MIhigherdim*. It is in fact a wrapper of a C++ program. This function was later parallelized through Parallel Computational Toolbox, with a *parfor* structure. 17

Figure 12 shows execution times for setup B, for Matlab code implementation of MI function. This function was downloaded from MILCA's toolbox website, as a function called *MIhigherdim*. It is in fact a wrapper of a C++ program. This function was later parallelized through Parallel Computational Toolbox, with a *parfor* structure. 18

Figure 13 shows execution times for setup A, for C-mex implementation of MI function. 19

Figure 14 shows execution times for setup B, for C-mex implementation of MI function. 20

Figure 15 shows execution times for setup A, comparing all previous MI implementations for a setup of 64 channels and different sample lengths. 21

Figure 16 shows execution times for setup B, comparing all previous MI implementations for a setup of 64 channels and different sample lengths. 22

Figure 17 show execution times for setup A, for Matlab code implementation of GS function. This function is a freely available .m function in Daniel Chicharro's publication Supplementary Material...... 24

Figure 18 show execution times for setup B, for Matlab code implementation of GS function. This function is a freely available .m function in Daniel Chicharro's publication Supplementary Material...... 25

Figure 19 show execution times for setup A, for a Matlab code implementation of GS function. This function is a freely available .m function in Daniel Chicharro's publication Supplementary Material, and then parallelized through Parallel Computational Toolbox, through a *parfor* structure. 26

Figure 20 show execution times for setup B, for a Matlab code implementation of GS function. This function is a freely available .m function in Daniel Chicharro's publication Supplementary Material, and then parallelized through Parallel Computational Toolbox, through a *parfor* structure. 27

Figure 21 shows execution times for setup A, for C-mex implementation of GS indices. 28

Figure 22 shows execution times for setup B, for C-mex implementation of GS indices. 29

Figure 23 show execution times for setup A, comparing all the different implementations of Generalized Synchronization indices implementations for a setup of 64 channels and different number of sensors..... 30

Figure 24 show execution times for setup B, comparing all the different implementations of Generalized Synchronization indices implementations for a setup of 64 channels and different number of sensors..... 31

Figure 25 shows speedup ratio in setup A, between C-mex implementation of each index implemented in this work and previous implementations within Brain Connectivity Toolbox. 33

Figure 26 shows speedup ratio in setup A, between C-mex implementation of each index implemented in this work and previous implementations within Brain Connectivity Toolbox. 34

2. Software Configuration

All results in this work have been programmed, compiled, executed and measured with Mathworks' Matlab 8.1.0.604 (R2013a) development environment with the following toolboxes installed: Digital Signal Processing Toolbox v8.4, Signal Processing Toolbox v6.19, Parallel Computing Toolbox v6.2, Statistics Toolbox v8.2 in a Windows 8.1 Pro 64-bit operating system.

On every trial, each function is called once before execution time is measured, and we report mean averages of 35 execution times obtained with Matlab's built-in functions *tic,toc;* (with negligible error considering times reported). We report time-series length in terms of number of samples instead of times, in order to account for different sampling frequencies at the same time.

When using Parallel Computational Toolbox based parallelization a parallel pool was started and 12 workers where used in setup A and 8 workers in setup B.

Different versions of each implementation have been developed, Matlab scripts and C/C++ implementations. Matlab MEX-files[1] development application interface has enabled us to write these custom C/C++ programs to be called as regular Matlab functions, adding the convenience of Matlab's environment to a more efficient execution. However, while usual Matlab scripts have a platform independent extension *.m*, MEX-files have platform-specific extensions. All MEX implementations have been compiled with Microsoft Visual Studio 2010 Ultimate.

We use latest version (3.3.4, released Nov. 2014) precompiled *.dll* files of the FFTW library[2] for computing the discrete Fourier transform and HT of arbitrary input sizes. This library is free software, distributed under the terms of the GNU General Public License with versions for all mayor operating systems, and to the best of our knowledge according to the benchmarks publicly available at http://www.fftw.org/benchfft/, FFTW's performance is typically superior to that of other publicly available FFT software and is even competitive with vendor-tuned codes.

As underlined previously, we opted for developing all functions using single precision floating point operations. However, for convenience, as it is the most frequently used precision in Matlab, all functions accept double precision point variables and the translation is done internally. Equally, output variables are transformed into double precision floating point in order to increase convenience while using each function within Matlab's environment.

3. Hardware Setup Description

3.1. Setup A:

- Motherboard: ASUSTeK Computer Inc. Z9PE-D8 WS (Socket-R 2011).
- CPU: 2x Intel Xeon E5 2690v2 3.00GHz 25MB Cache. Hyper-threading enabled.
- Memory: 64GB ECC DDR3 802MHz (11-11-11-28).
- Graphics Card: Nvidia Tesla K40c rev A2 Driver version 9.18.13.3288.
- Hard-drive: 128GB Toshiba SSD SATA-III 6.0Gb/s.

3.2. Setup B:

- Motherboard: ASUSTeK Computer Inc. P9X79 (LGA2011).
- CPU: Intel Core i7-4820K IvyBridge-E 22nm 3.7GHz. Hyper-threading enabled.
- Memory: 32GB RAM, DDR3, non ECC, 667 MHz (9-9-9-24).
- Graphics Card: Nvidia GTX 650Ti (ASUSTek Comp. Inc.)
- Hard-drive: 2x 128GB Intel SSD SATA-III (Software based, Raid 0 Volume).

[1] MEX stands for Matlab Executable.
[2] http://www.fftw.org

4. Phase Synchronization Measures

Next figures show improvement in execution times of our four versions of Phase Syncrhonization measures. These versions are:

- BSX based Matlab implementation.
- PCT based parallelization of Matlab implementation.
- C-MEX based implementation.

We have tested all implementations measuring syncrhonization for random vectors of several lengths starting at 100 samples up to 30.000 samples and considering both hardware setups and for different number of sensors. Ooptimization for FFTW was set to 1 (*FFTW_MEASURE*, see FFTW documentation for further details).

Times where measured with *tic;toc* Matlab's built-in functions, as described in the following procedure:

As we use FFTW we specify mode "2" for computation of Fourier Transforms, which in terms of the FFTW library corresponds to "FFTW_ESTIMATE" .

i. Randomnly define matrix with columns = number of sensors and rows = number of samples.
ii. Define phases near borders to discard as $N_{samples}/10$.
iii. Compute PS for all pairs of sensors once.
iv. Call *tic*.
v. Compute PS for all pairs of sensors once.
vi. Call *toc*.
vii. Repeat steps iii to iv 35 times.
viii. Save time mean average of all 35 repetitions.

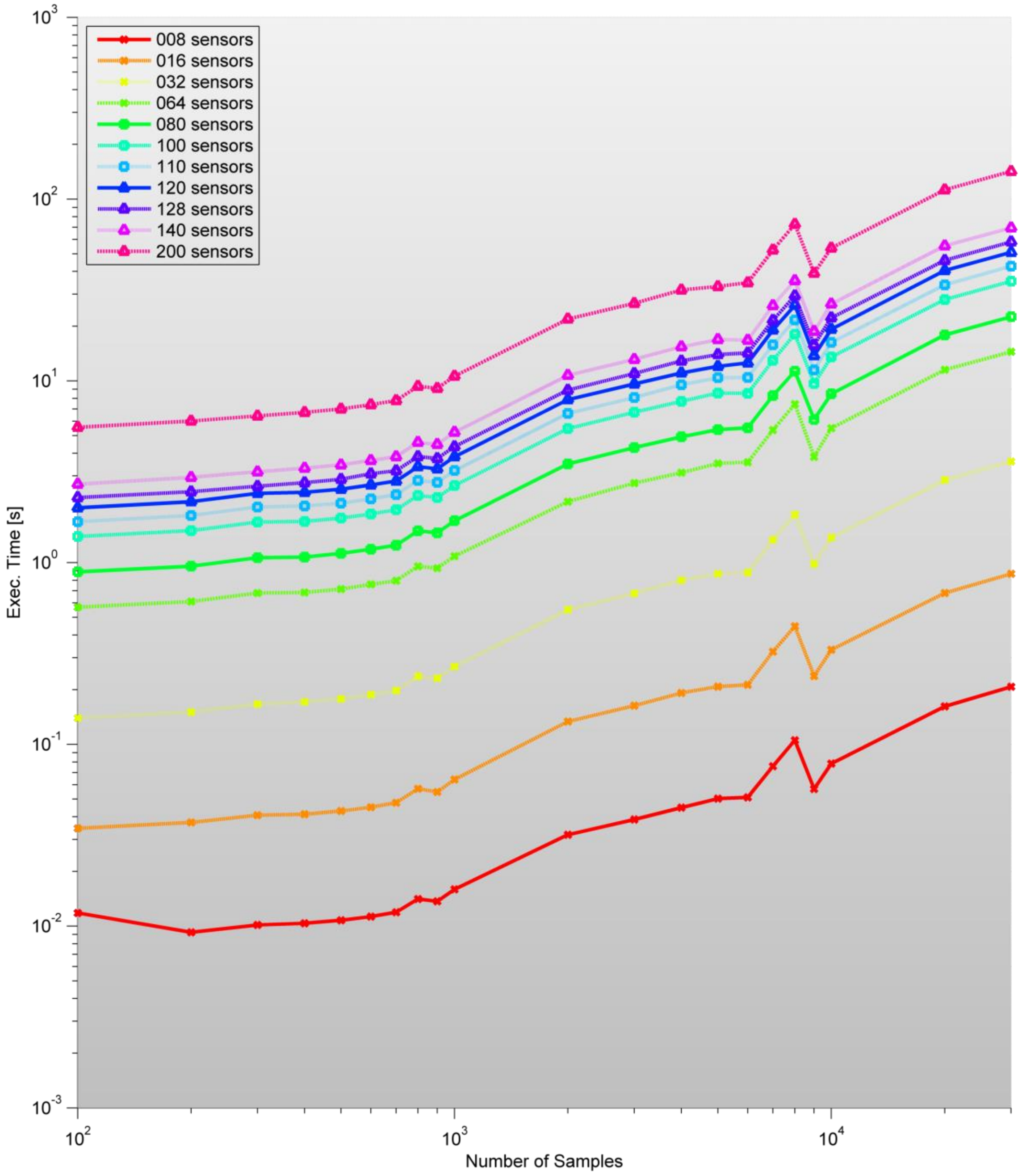

Figure 1 shows execution times for Matlab code implementation of Phase Synchronization indices for setup A. The implementation is based on bsxfun function.

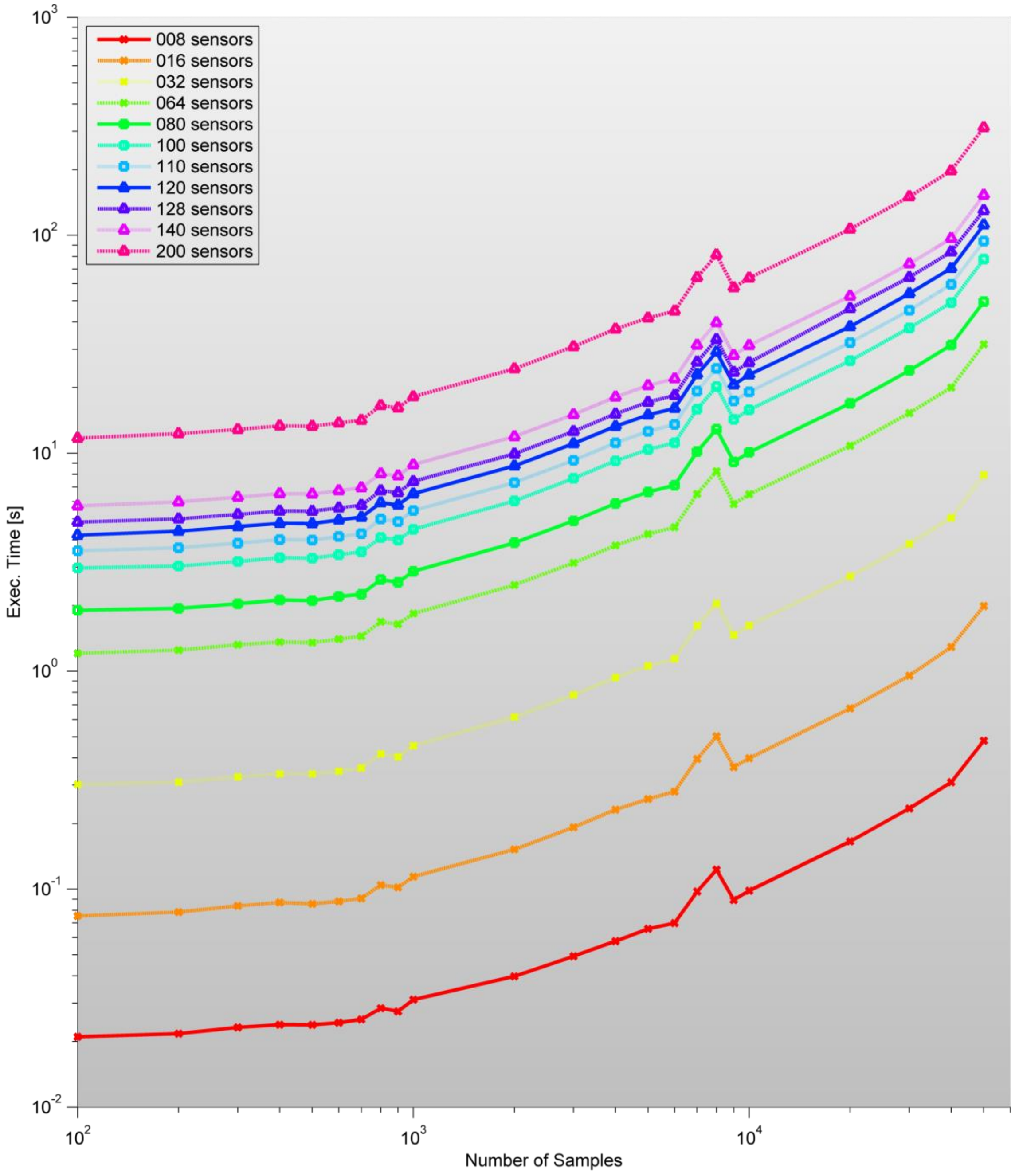

Figure 2 shows execution times for Matlab code implementation of Phase Synchronization indices for setup B. The implementation is based on bsxfun function.

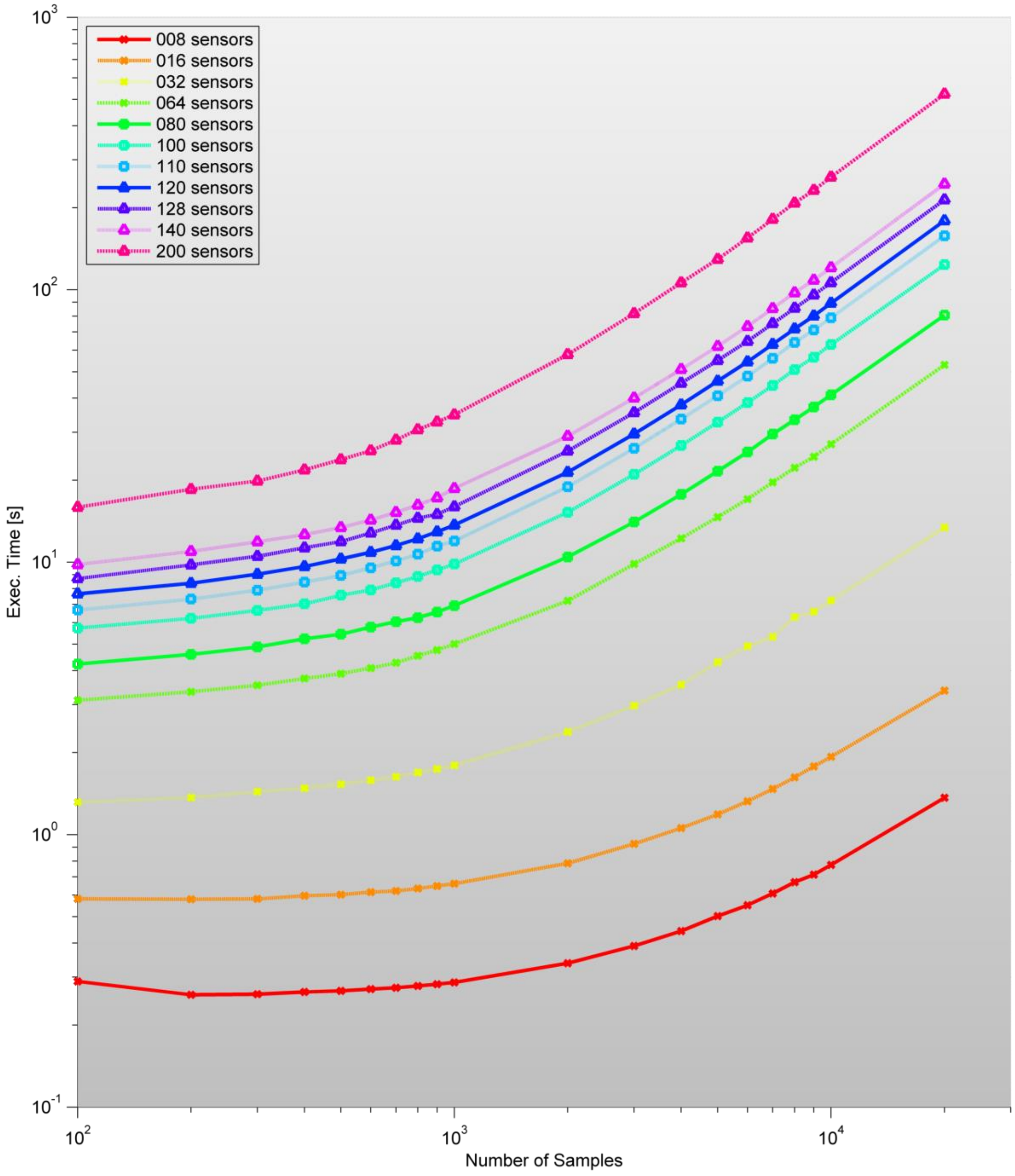

Figure 3 shows execution times for Matlab code implementation of Phase Synchronization indices for setup A, parallelized through Parallel Computational Toolbox, using *parfor* structure. The implementation is based on bsxfun function.

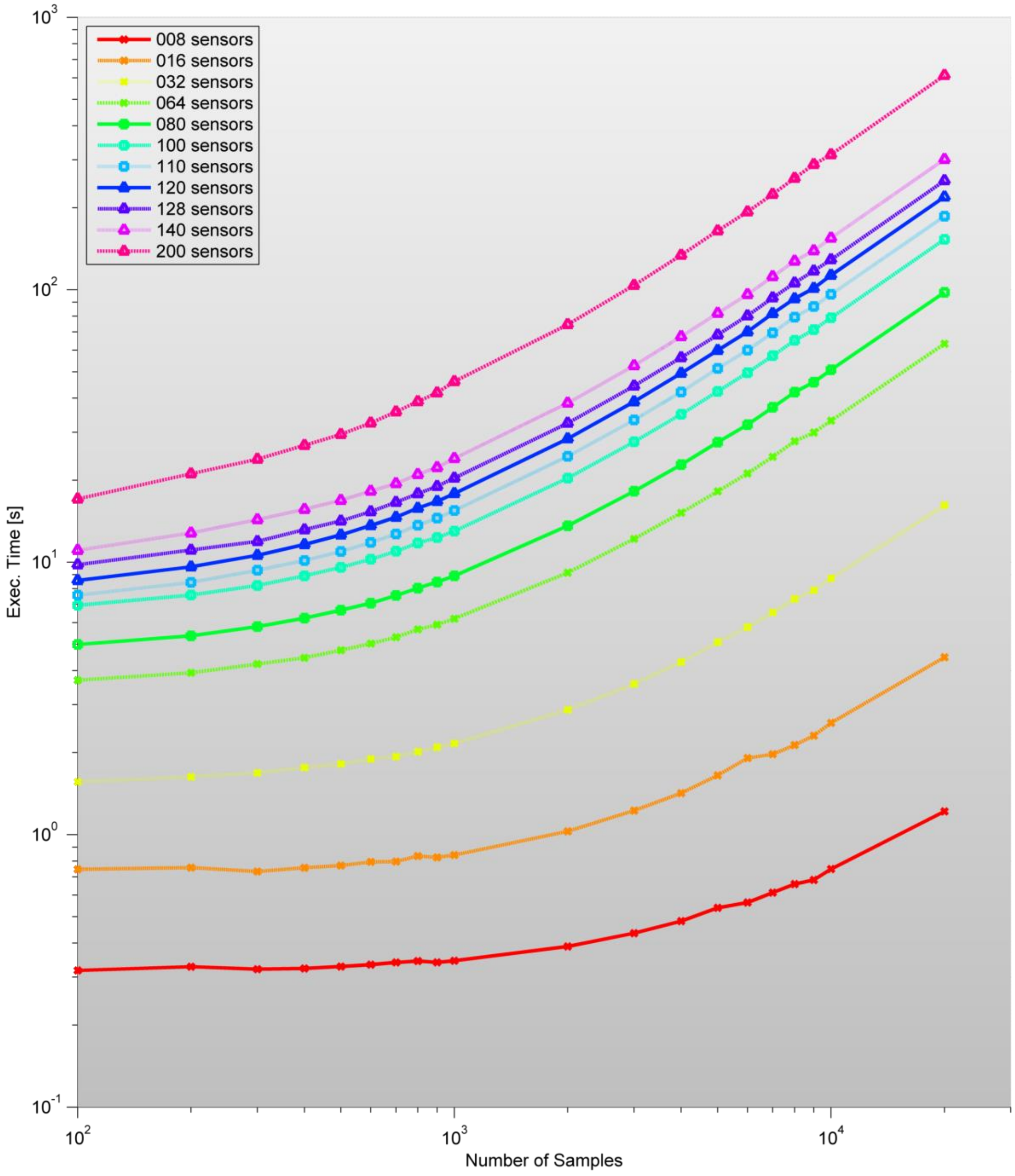

Figure 4 shows execution times for Matlab code implementation of Phase Synchronization indices for setup B, parallelized through Parallel Computational Toolbox, using *parfor* structure. The implementation is based on bsxfun function.

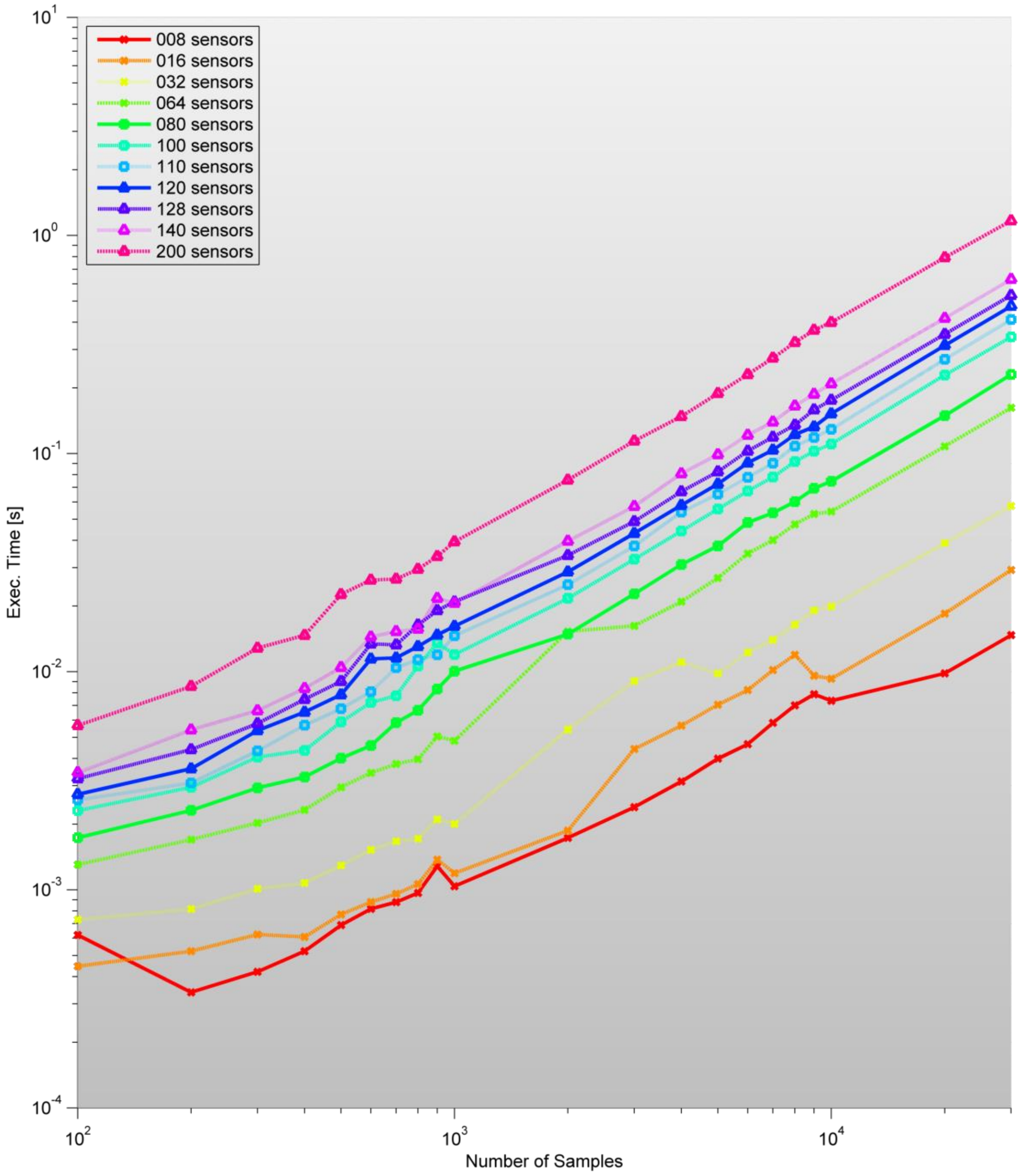

Figure 5 shows execution times for c-mex developed C code of Phase Synchronization indices for setup A, parallelized through OpenMP.

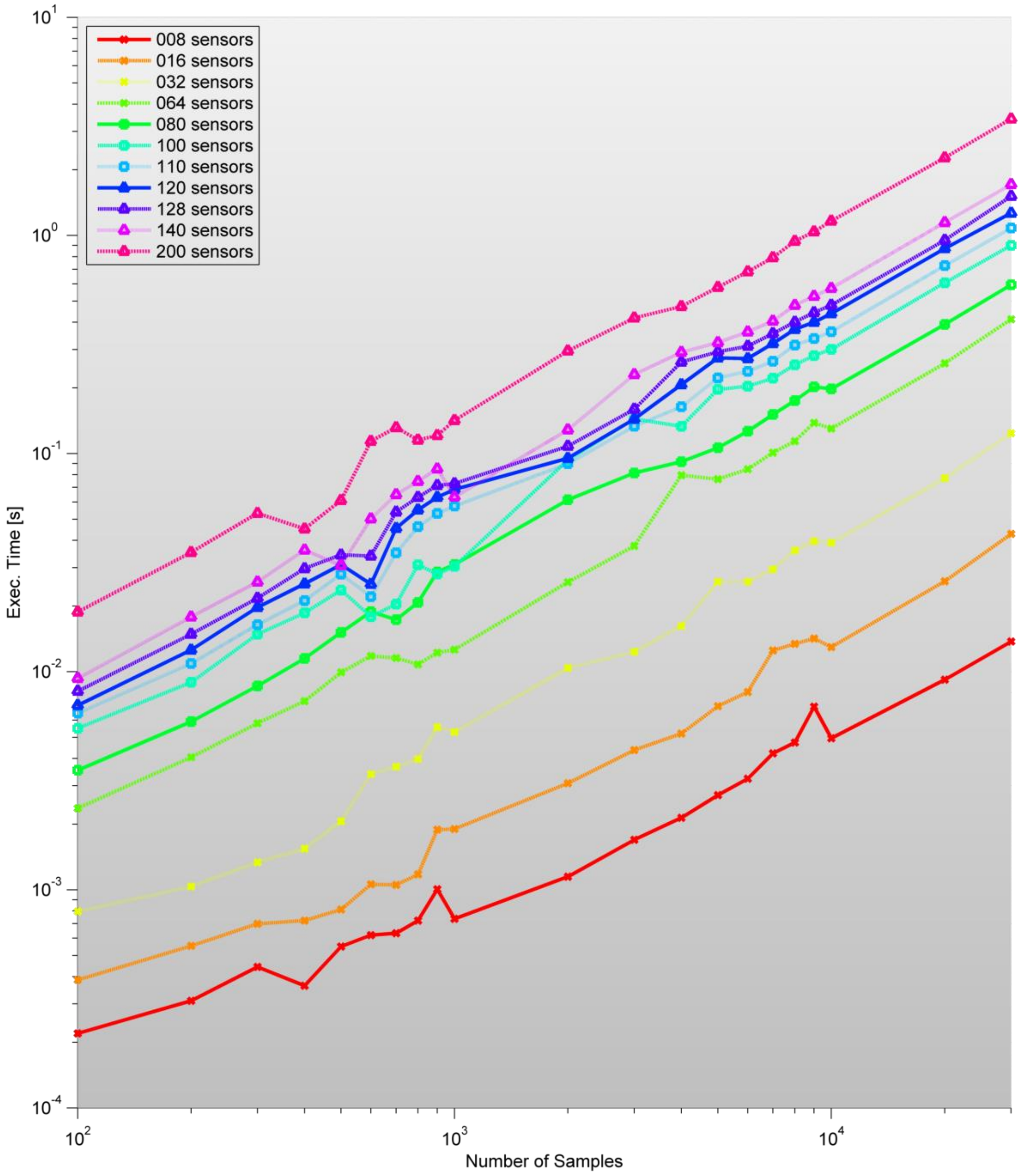

Figure 6 shows execution times for c-mex developed C code of Phase Synchronization indices for setup B, parallelized through OpenMP.

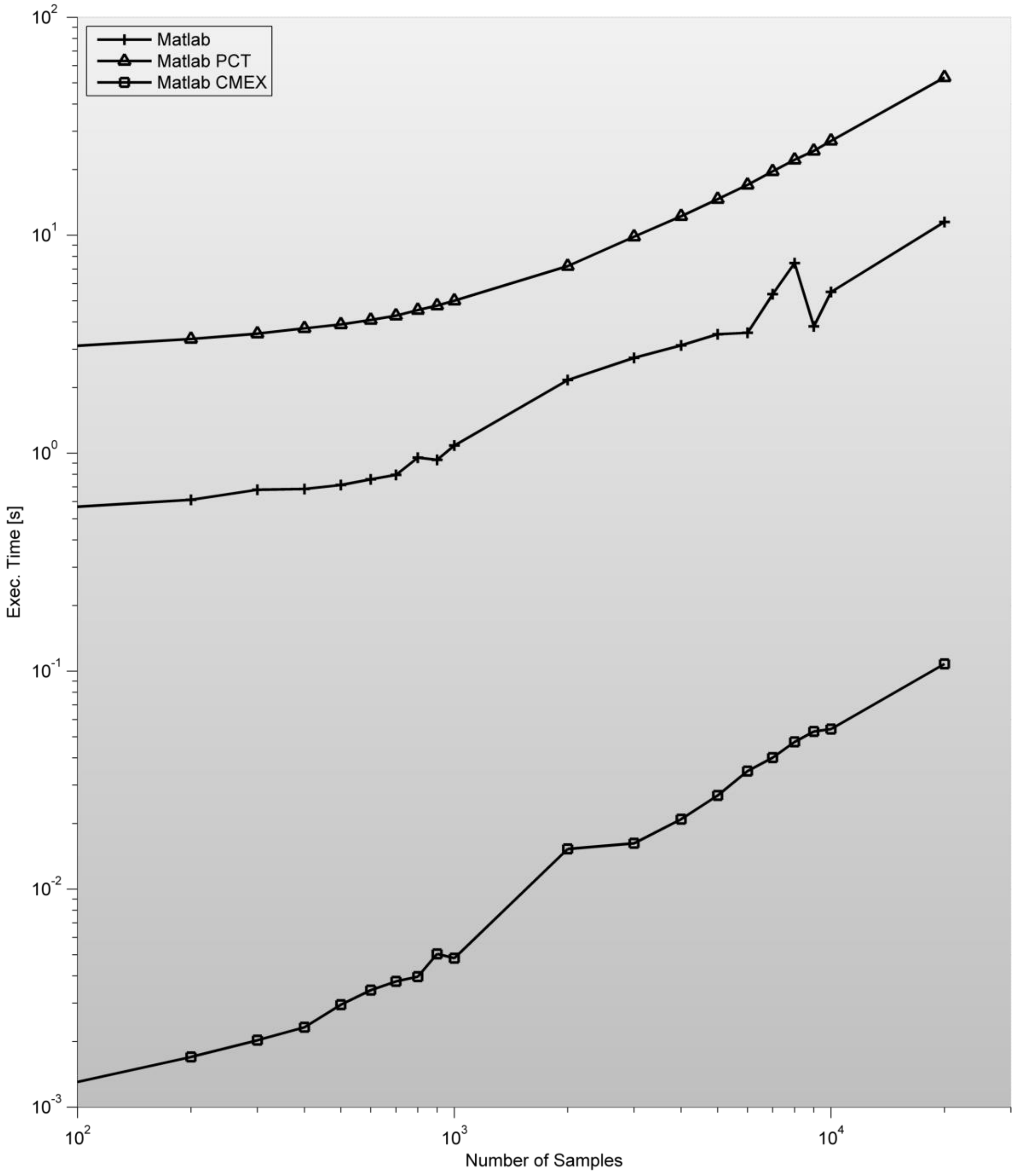

Figure 7 shows execution times in setup A for different implementations of Phase Synchronization indices for a 64 sensor setup and different sample lengths.

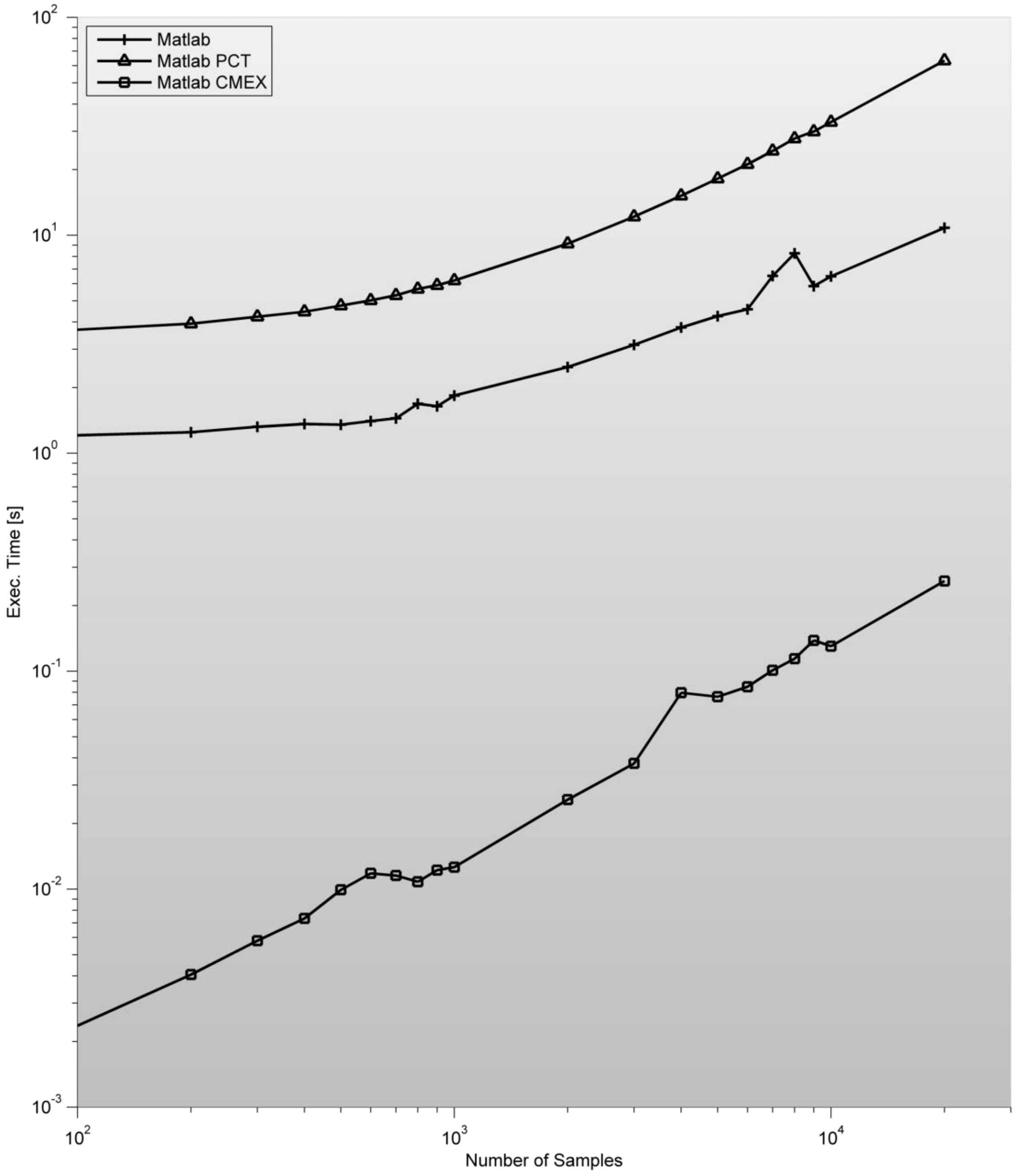

Figure 8 shows execution times in setup B for different implementations of Phase Synchronization indices for a 64 sensor setup and different sample lengths.

5. Mutual Information

Next figures show improvement in execution times of each implementation of a Mutual Information function. We have tested all implementations measuring execution times for several lengths starting at 100 samples up to 30.000 samples and considering both hardware setups and for different number of sensors.

Times where measured with *tic;toc* Matlab's built-in functions, as described in the following procedure:

ix. Randomnly define matrix with columns = number of sensors and rows = number of samples.
x. Compute MI for all pairs of sensors once.
xi. Call *tic*.
xii. Compute MI for all pairs of sensors once.
xiii. Call *toc*.
xiv. Repeat steps iii to iv 15 times.
xv. Save time mean average of all repetitions.

For all executions, MI parameters where set to an embedding dimension of M=3; a Tau=1 and 6 neighbors considered.

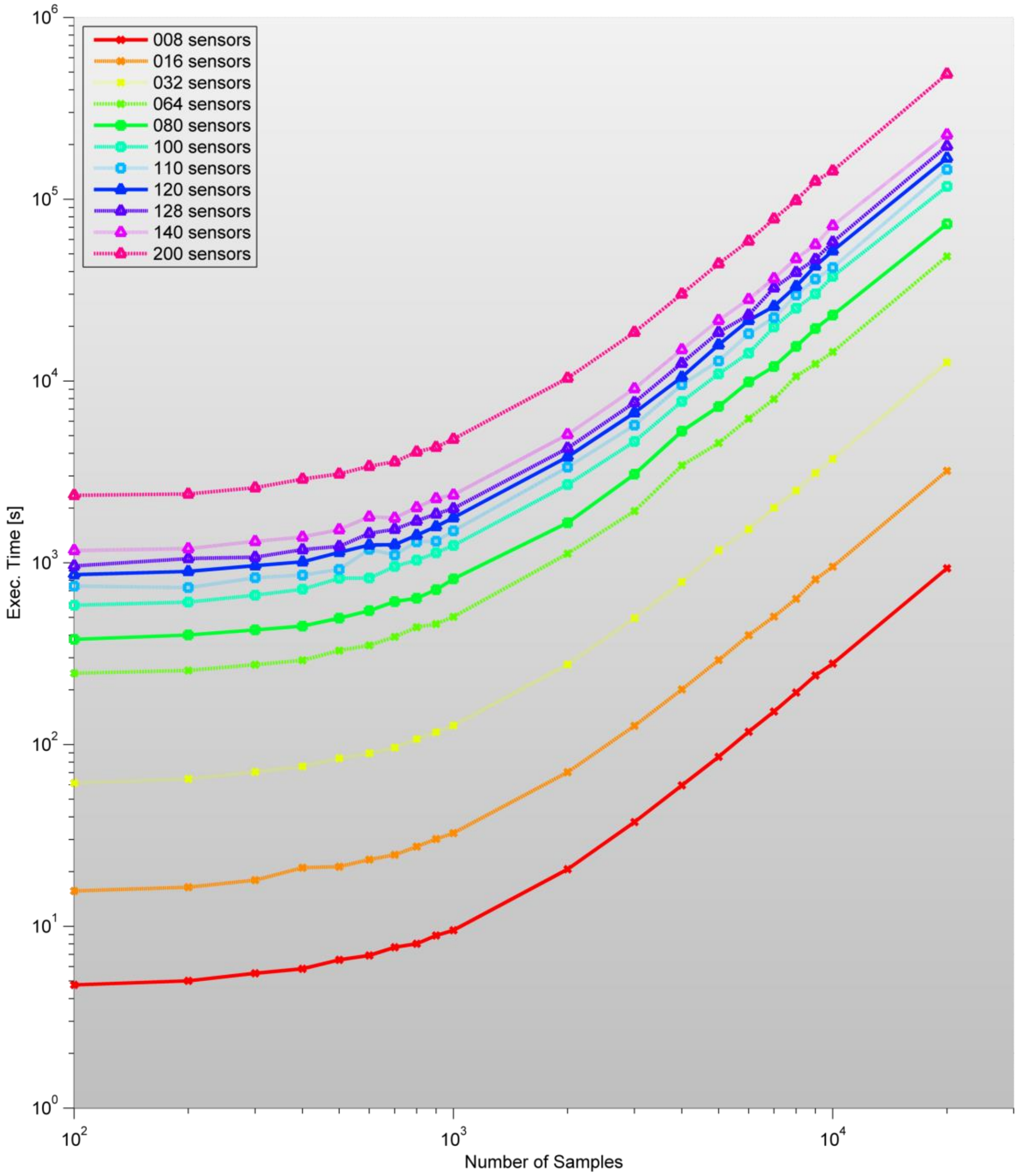

Figure 9 shows execution times in setup A, for Matlab code implementation of MI function. This function was downloaded from MILCA's toolbox website, as a function called *MIhigherdim*. It is in fact a wrapper of a C++ program.

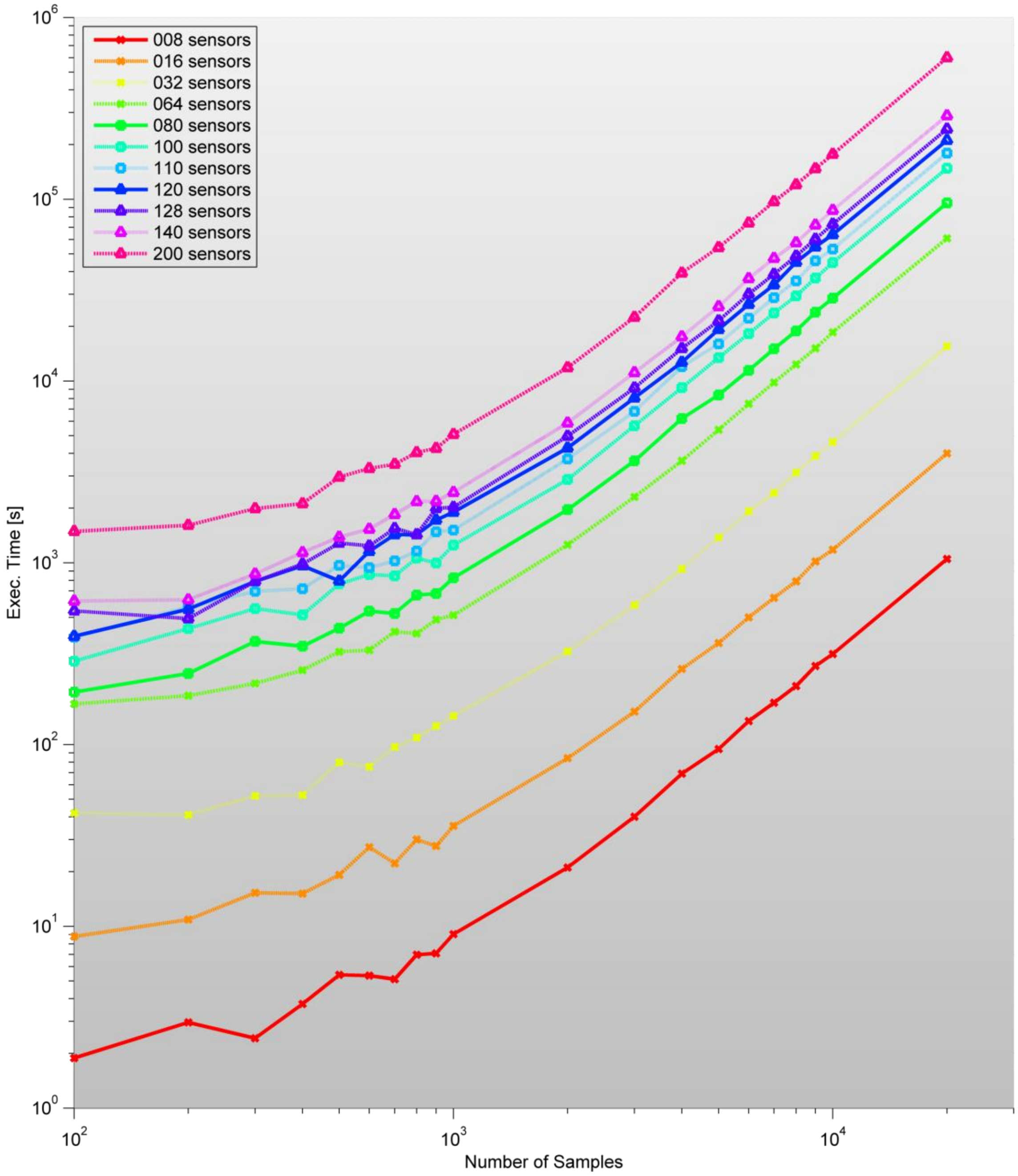

Figure 10 shows execution times in setup B, for Matlab code implementation of MI function. This function was downloaded from MILCA's toolbox website, as a function called *MIhigherdim*. It is in fact a wrapper of a C++ program.

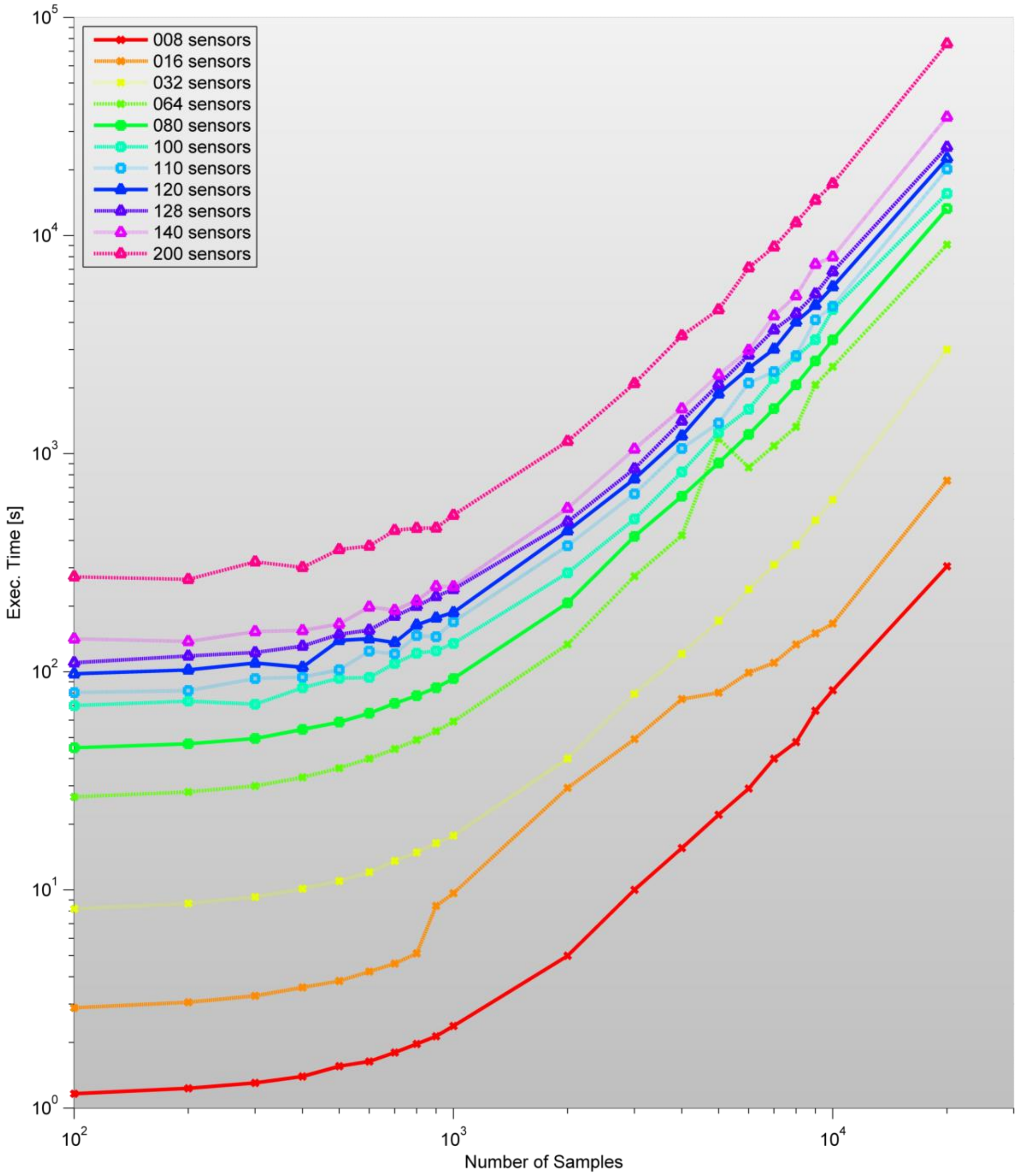

Figure 11 shows execution times for setup A, for Matlab code implementation of MI function. This function was downloaded from MILCA's toolbox website, as a function called *MIhigherdim*. It is in fact a wrapper of a C++ program. This function was later parallelized through Parallel Computational Toolbox, with a *parfor* structure.

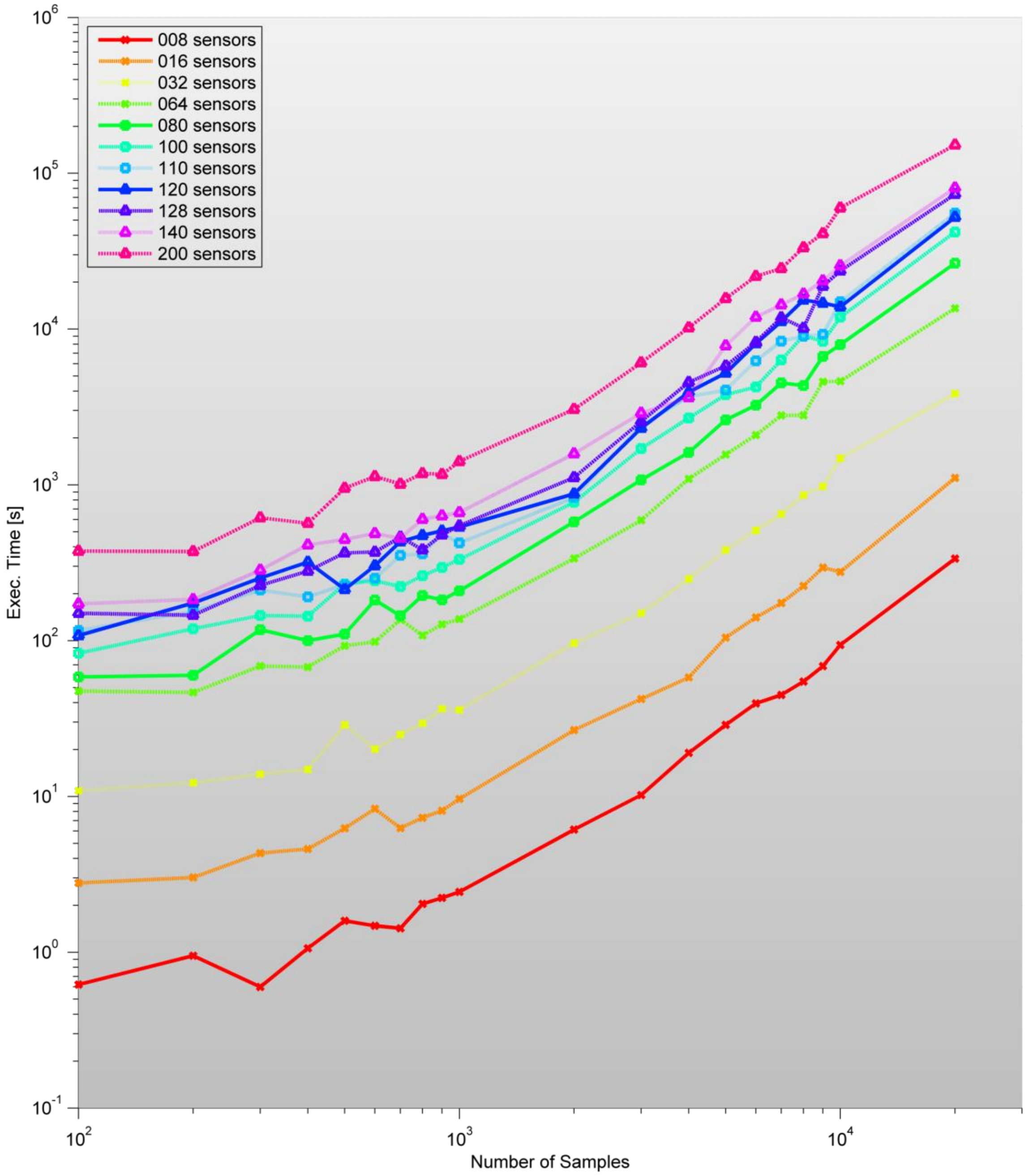

Figure 12 shows execution times for setup B, for Matlab code implementation of MI function. This function was downloaded from MILCA's toolbox website, as a function called *MIhigherdim*. It is in fact a wrapper of a C++ program. This function was later parallelized through Parallel Computational Toolbox, with a *parfor* structure.

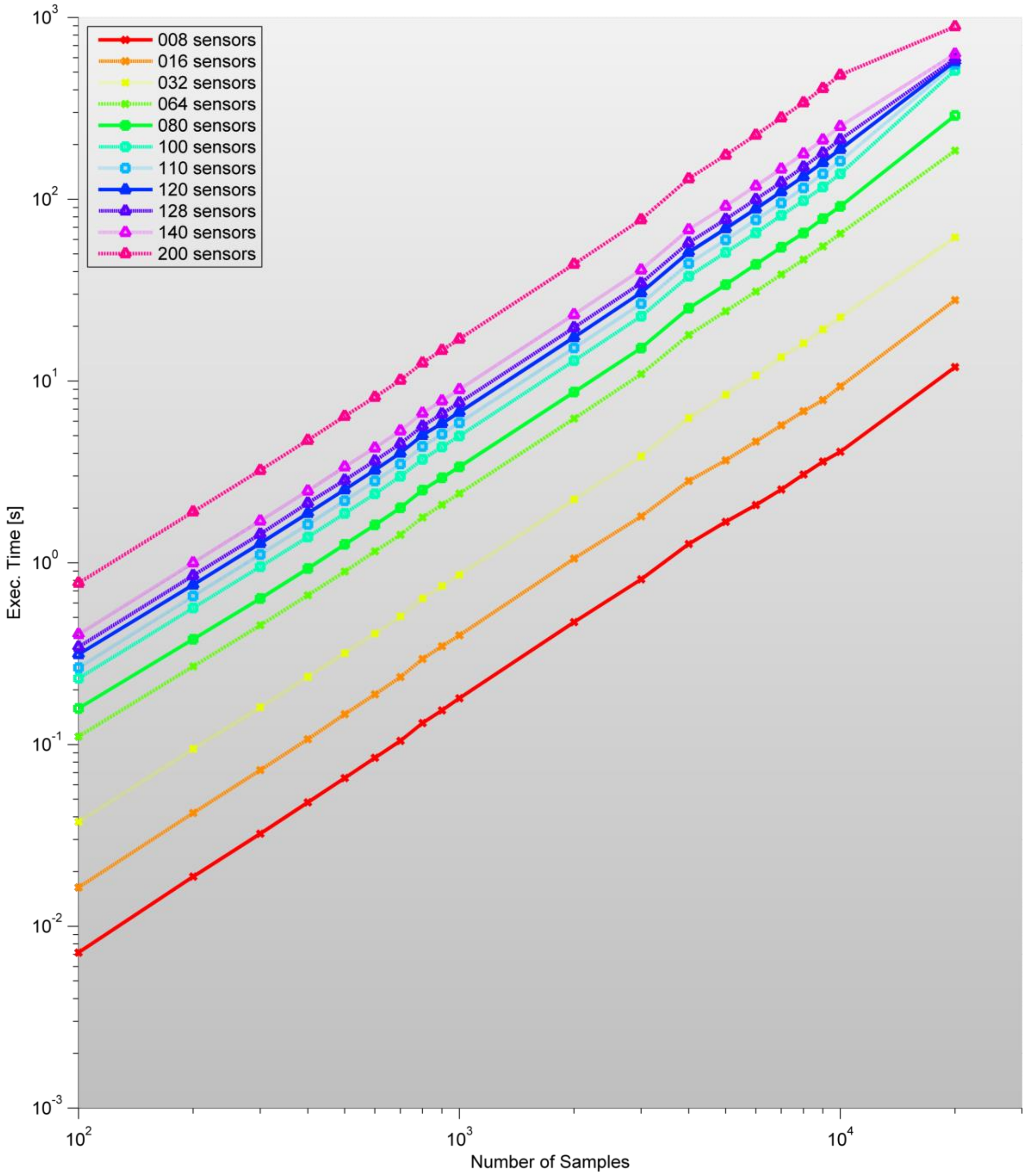

Figure 13 shows execution times for setup A, for C-mex implementation of MI function.

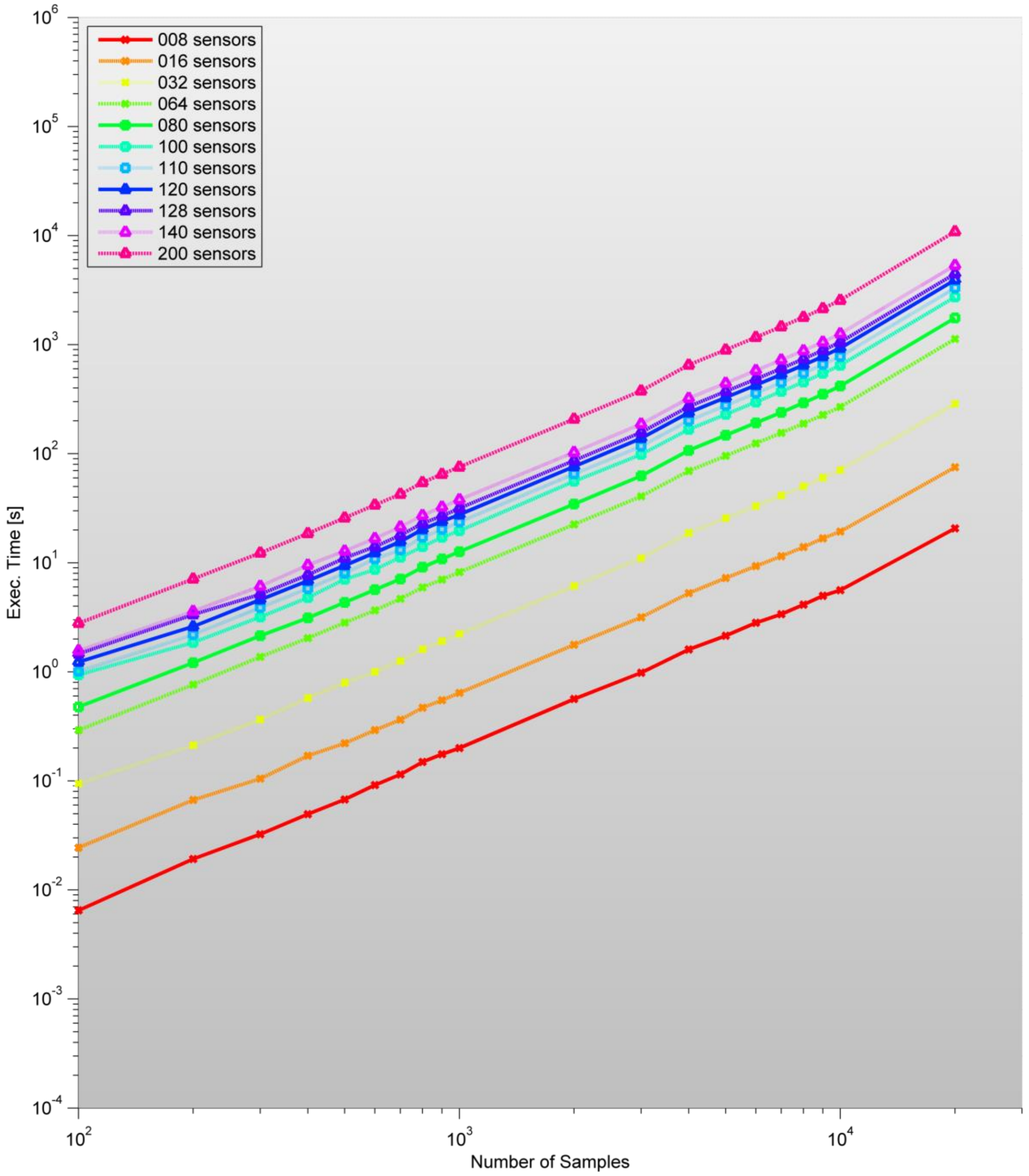

Figure 14 shows execution times for setup B, for C-mex implementation of MI function.

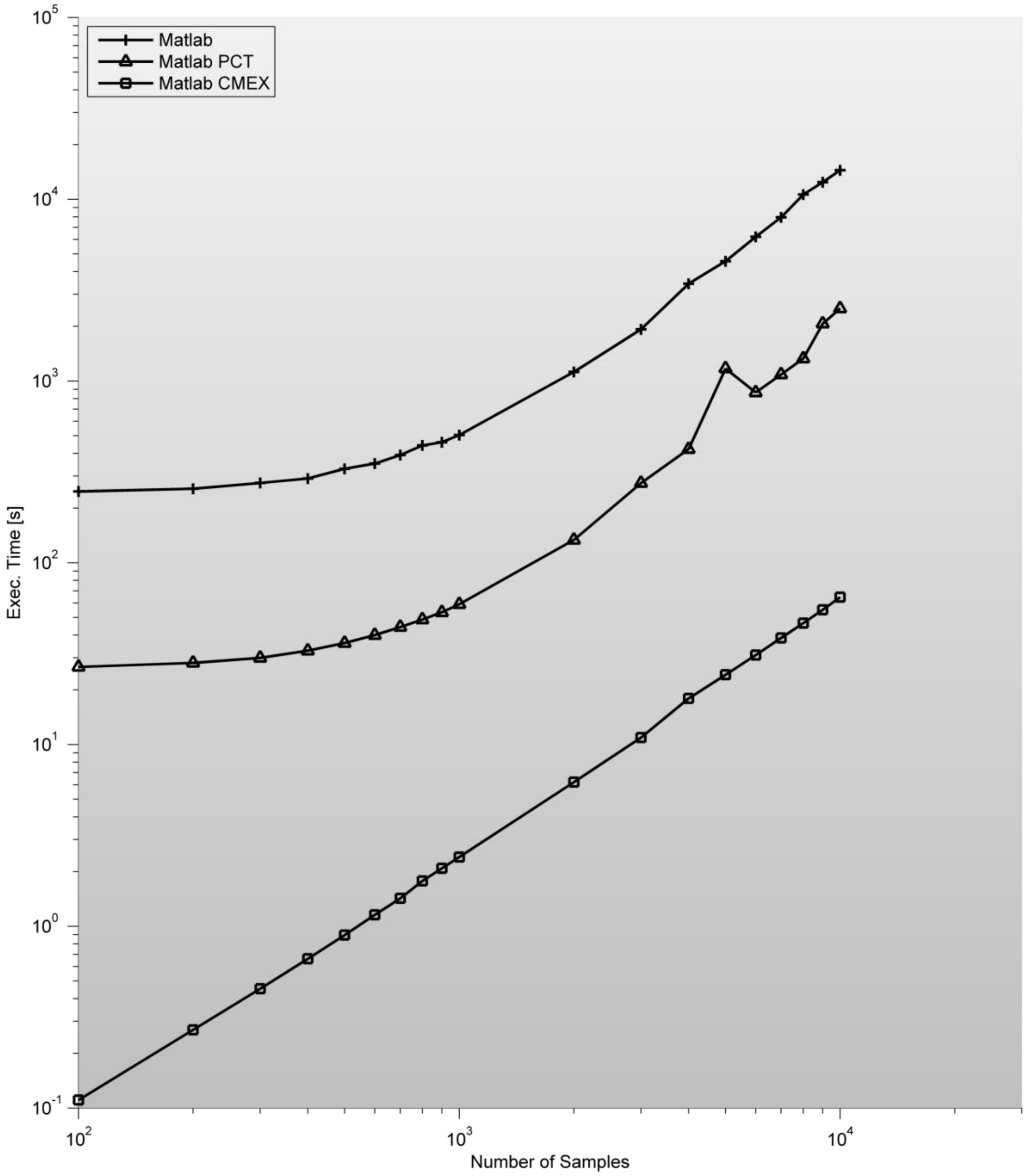

Figure 15 shows execution times for setup A, comparing all previous MI implementations for a setup of 64 channels and different sample lengths.

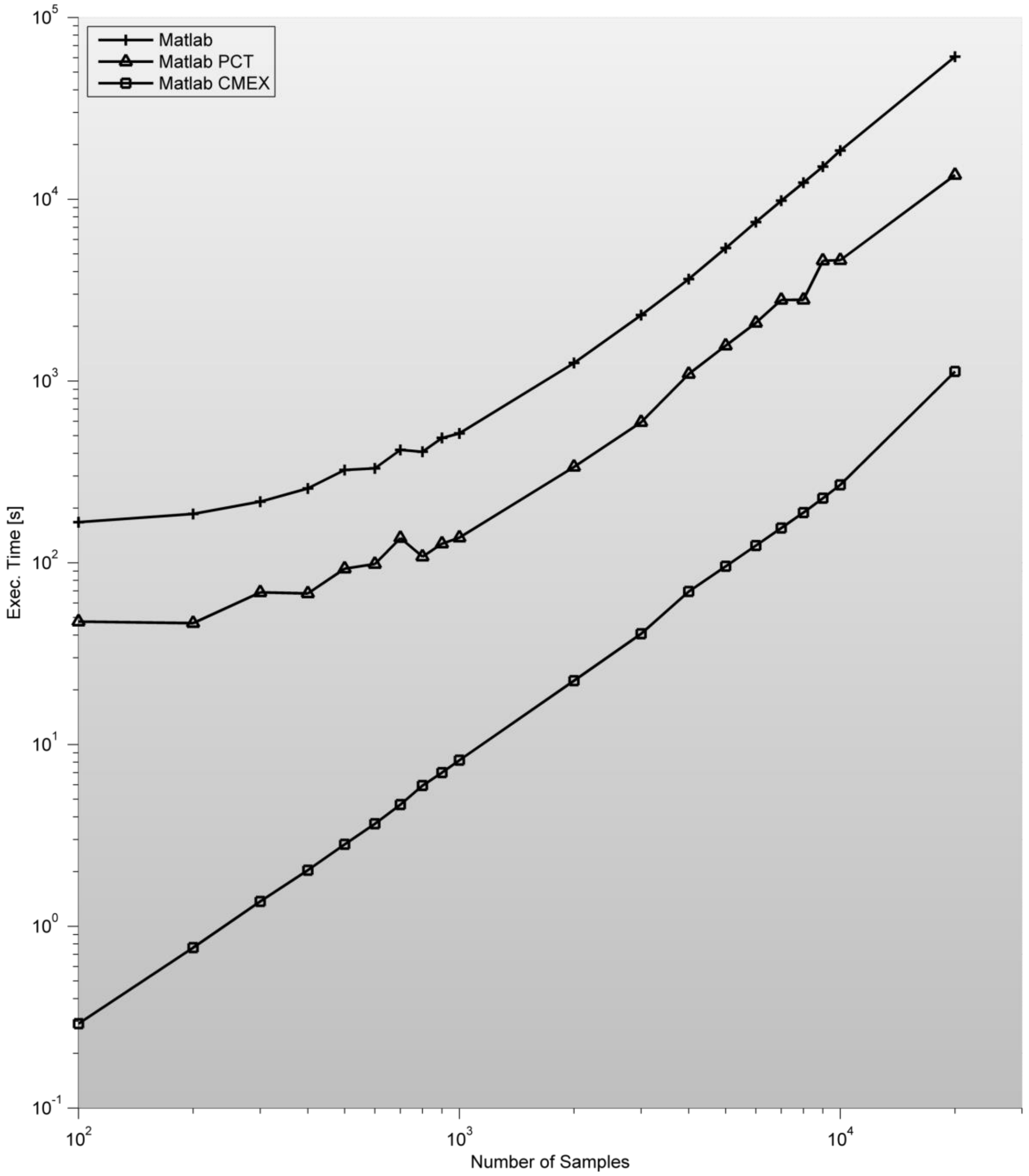

Figure 16 shows execution times for setup B, comparing all previous MI implementations for a setup of 64 channels and different sample lengths.

6. GS Indices

Next figures show improvement in execution times of each implementation of Generalised Syncrhonization indices S, H, M and L. We have tested all implementations measuring execution times for several lengths starting at 100 samples up to 30.000 samples and considering both hardware setups and for different number of sensors.

Times where measured with *tic;toc* Matlab’s built-in functions, as described in the following procedure:

i. Randomnly define matrix with columns = number of sensors and rows = number of samples.
ii. Compute GS indices for all pairs of sensors once.
iii. Call *tic*.
iv. Compute GS for all pairs of sensors once.
v. Call *toc*.
vi. Repeat steps iii to iv 15 times.
vii. Save time mean average of all repetitions.

For all executions, GS parameters where set to an embedding dimension of M=3, a Tau=1, 6 neighbors considered and a Theiler correction window of 2.

Figures 17 and 18 show execution times for setup A and B, respectively, for Matlab code implementation of GS function. This function is in fact a freely available .m function in Daniel Chicharro’s publication Supplementary Material[3]. Note that this implementation, even after vectorization and using the optimized function psdist2.m included in the Statistical Toolbox, is not as efficient as the C-mex implementation parallelized through OpenMP. Computation of GS is very expensive both in terms of memory and time, as it involves ordering distances between states $O(N^2)$.

[3] http://journals.aps.org/pre/abstract/10.1103/PhysRevE.80.026217

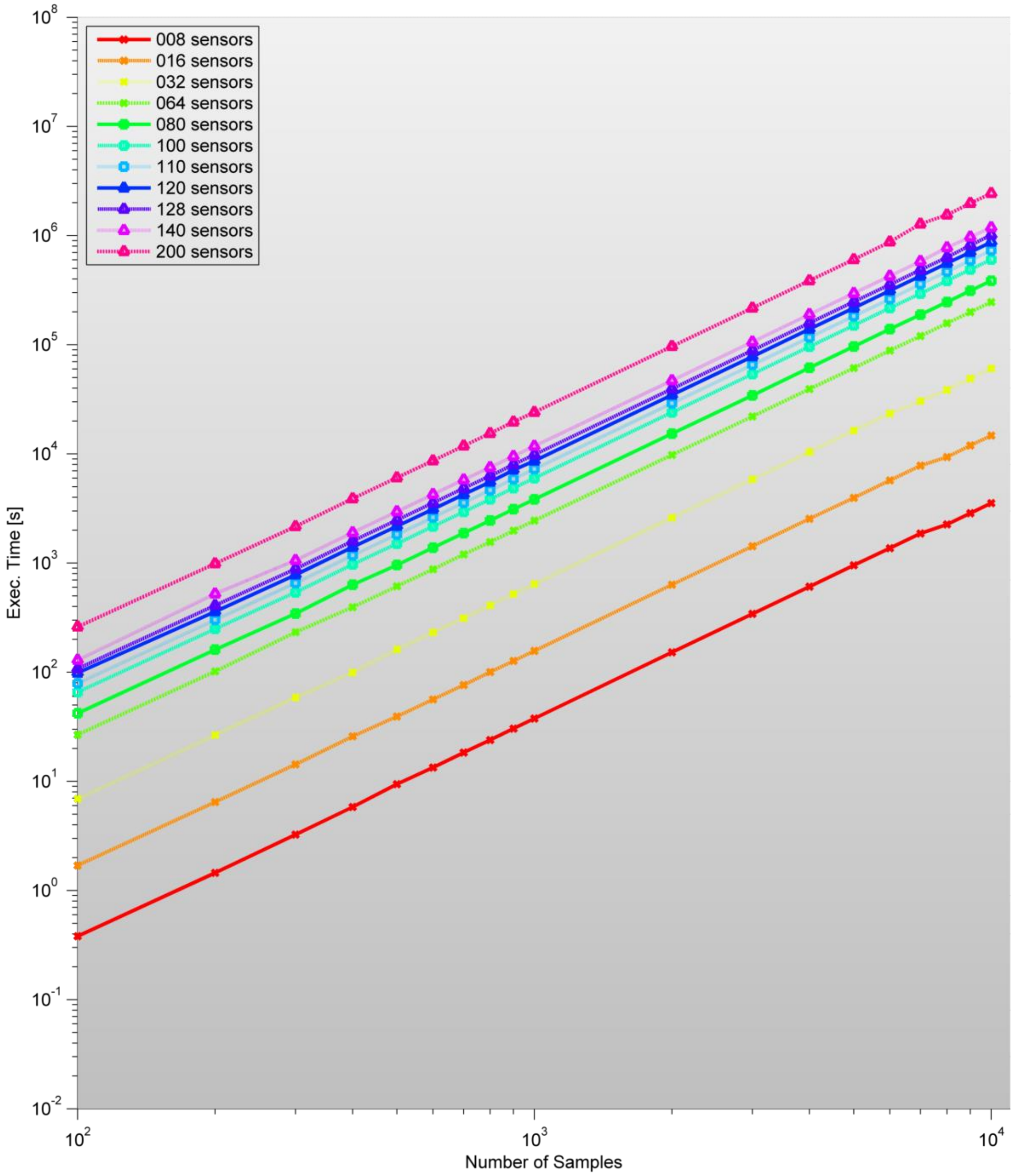

Figure 17 show execution times for setup A, for Matlab code implementation of GS function. This function is a freely available .m function in Daniel Chicharro's publication Supplementary Material.

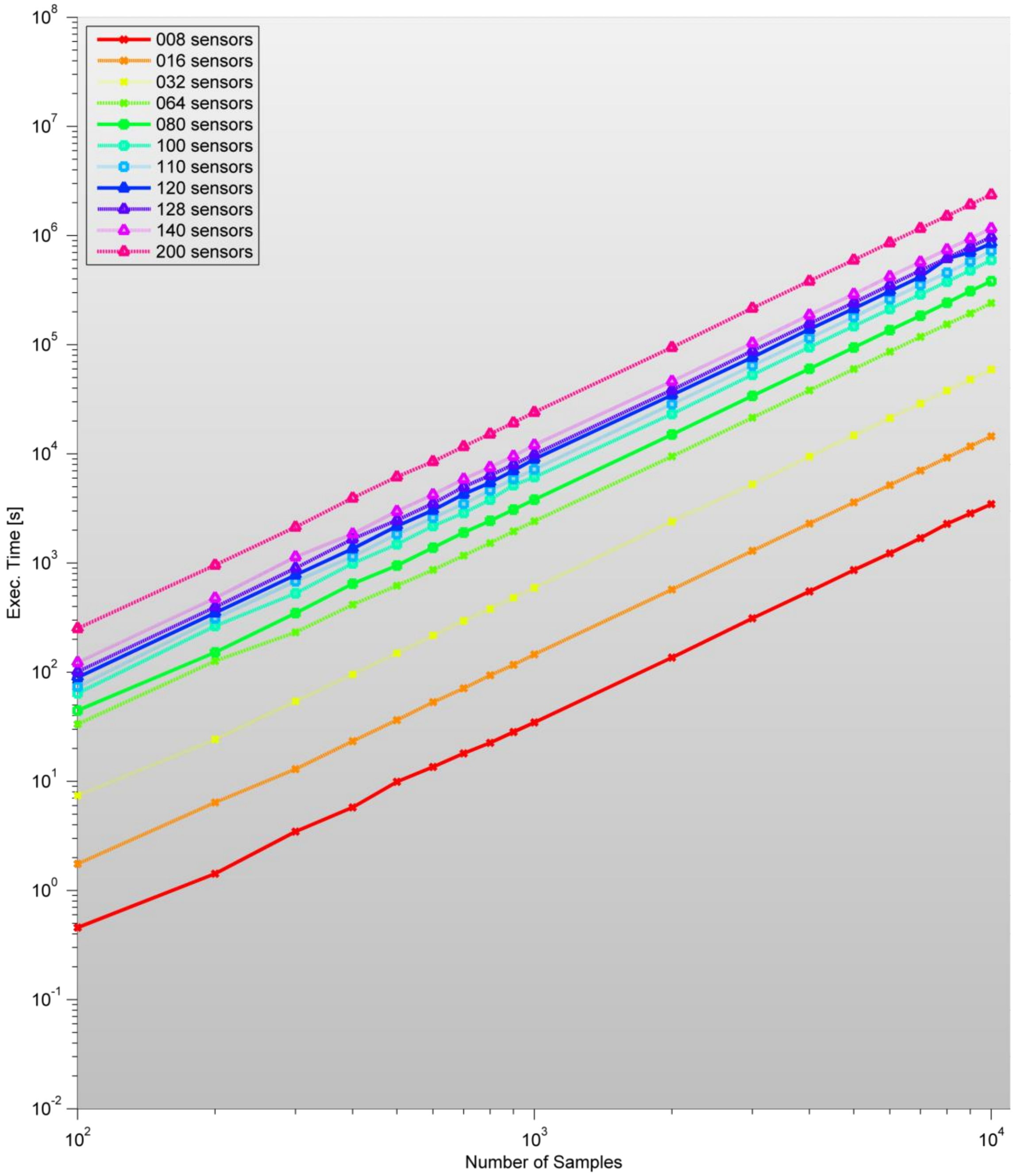

Figure 18 show execution times for setup B, for Matlab code implementation of GS function. This function is a freely available .m function in Daniel Chicharro's publication Supplementary Material.

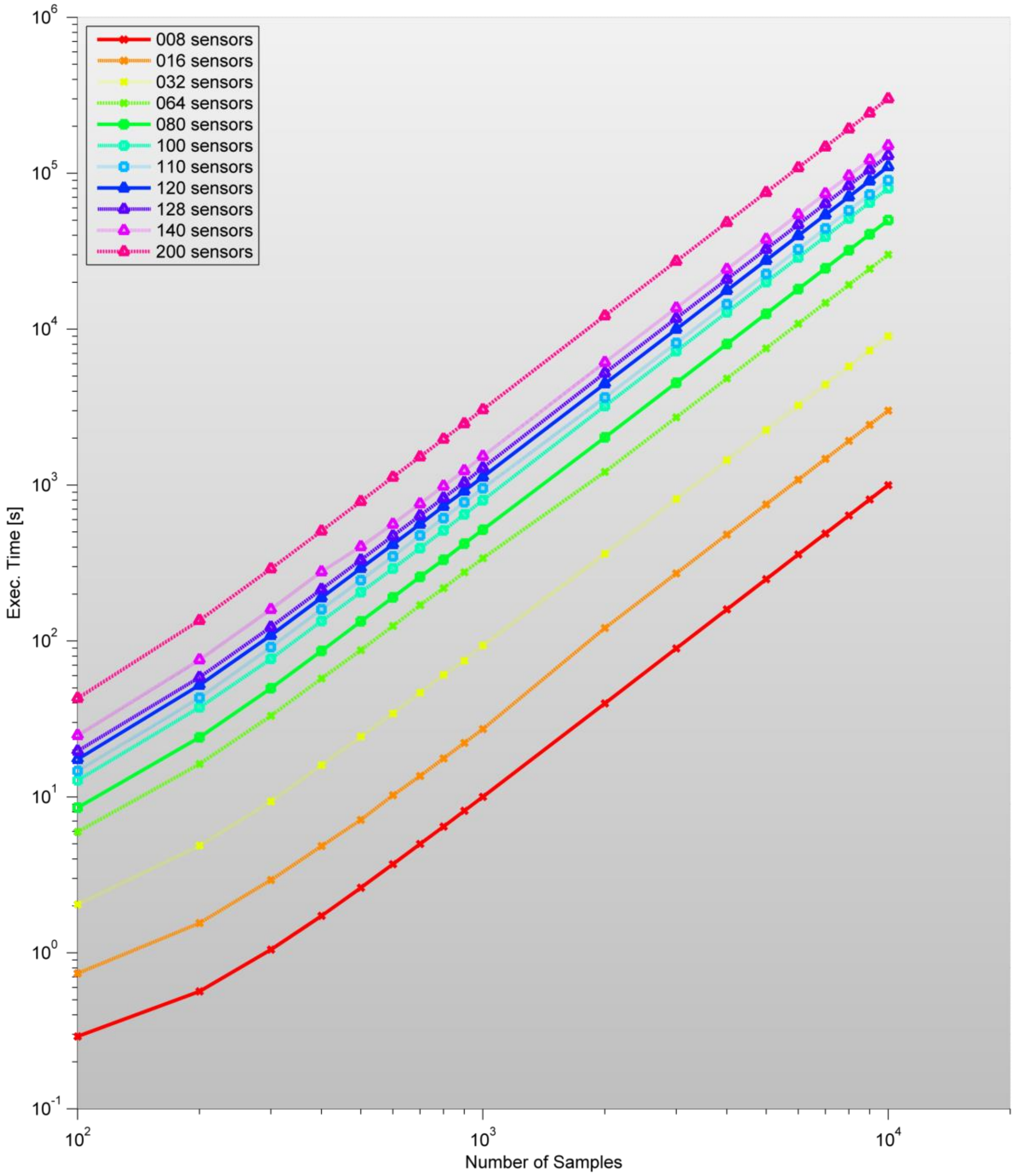

Figure 19 show execution times for setup A, for a Matlab code implementation of GS function. This function is a freely available .m function in Daniel Chicharro's publication Supplementary Material, and then parallelized through Parallel Computational Toolbox, through a *parfor* structure.

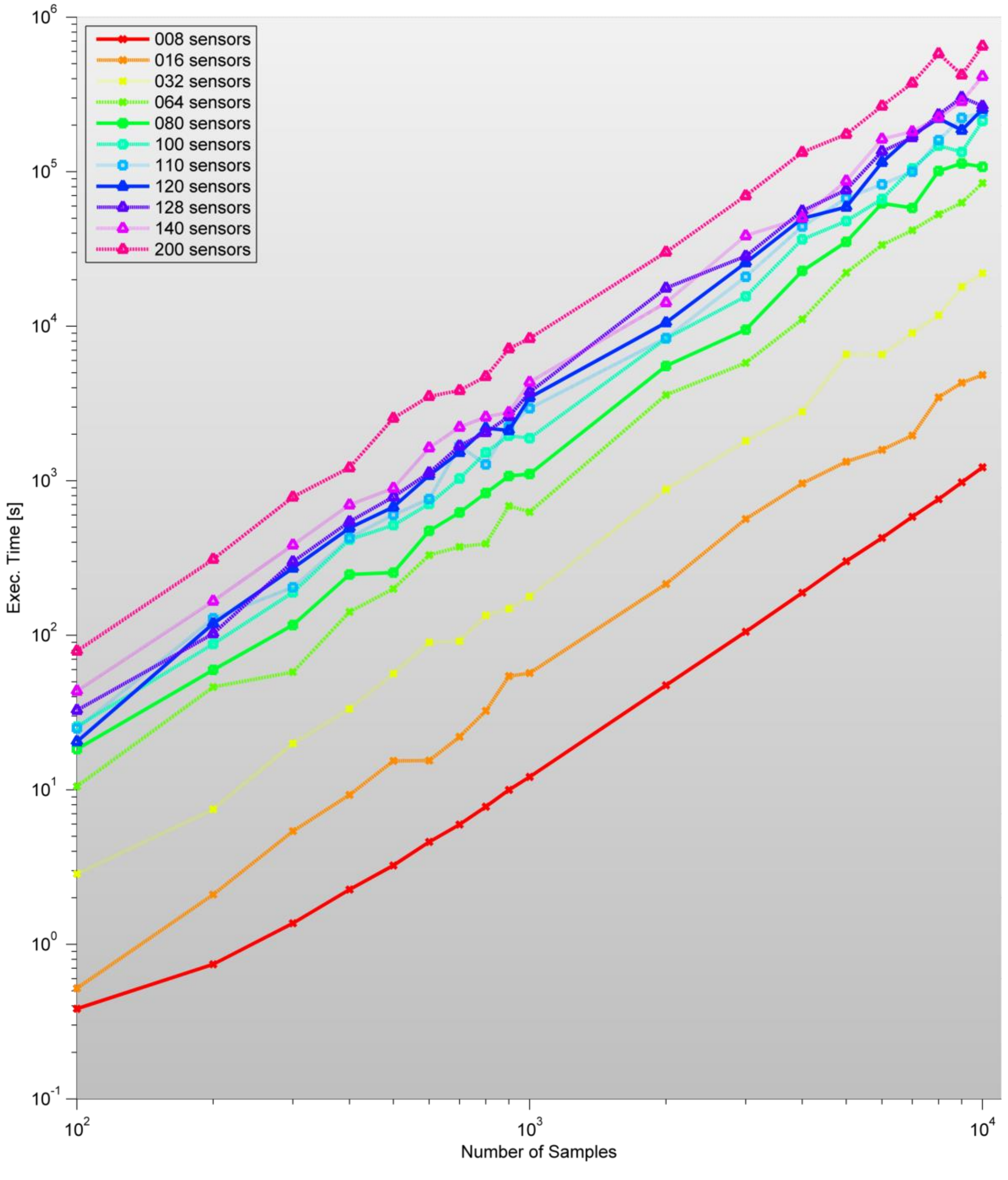

Figure 20 show execution times for setup B, for a Matlab code implementation of GS function. This function is a freely available .m function in Daniel Chicharro's publication Supplementary Material, and then parallelized through Parallel Computational Toolbox, through a *parfor* structure.

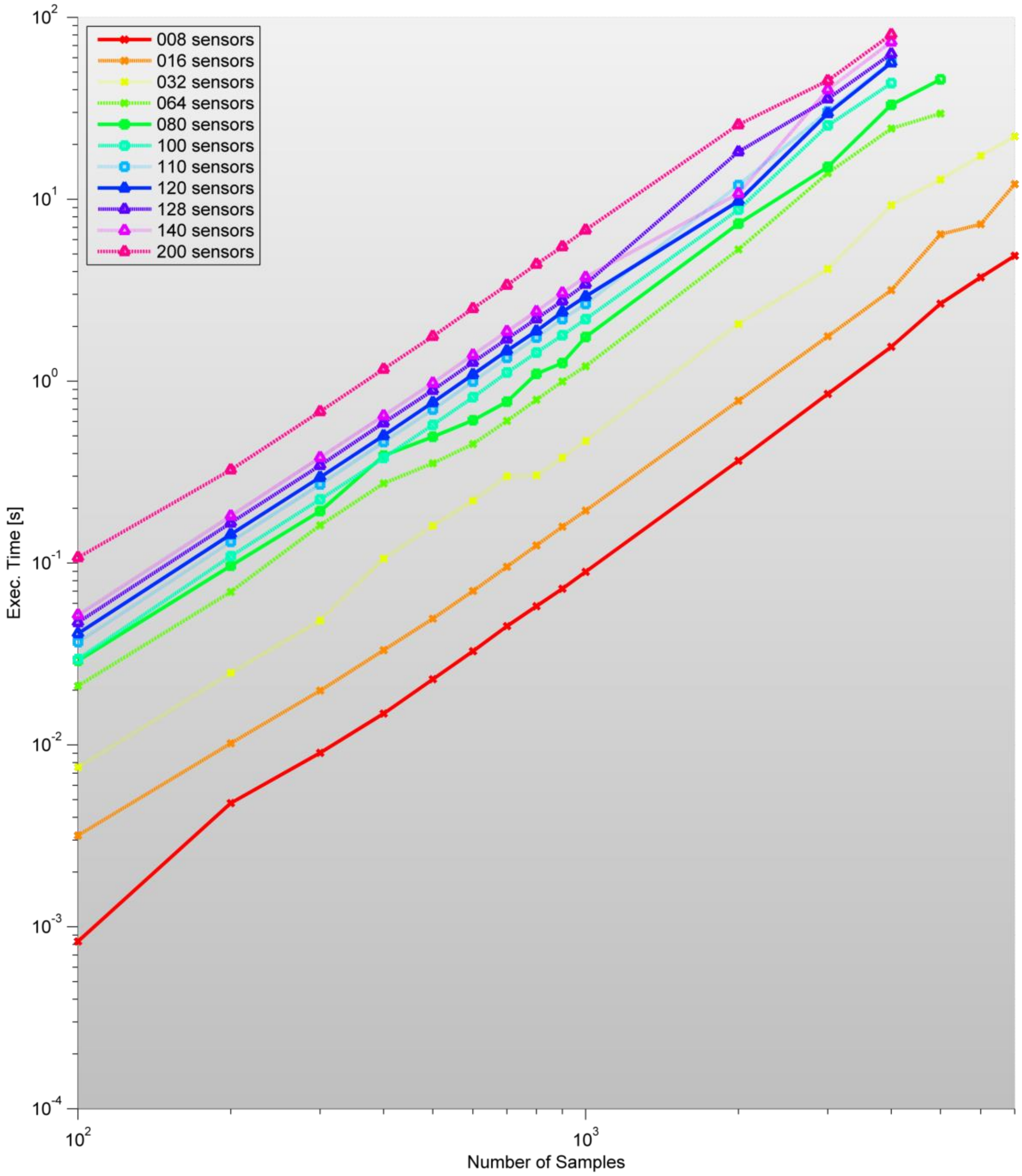

Figure 21 shows execution times for setup A, for C-mex implementation of GS indices.

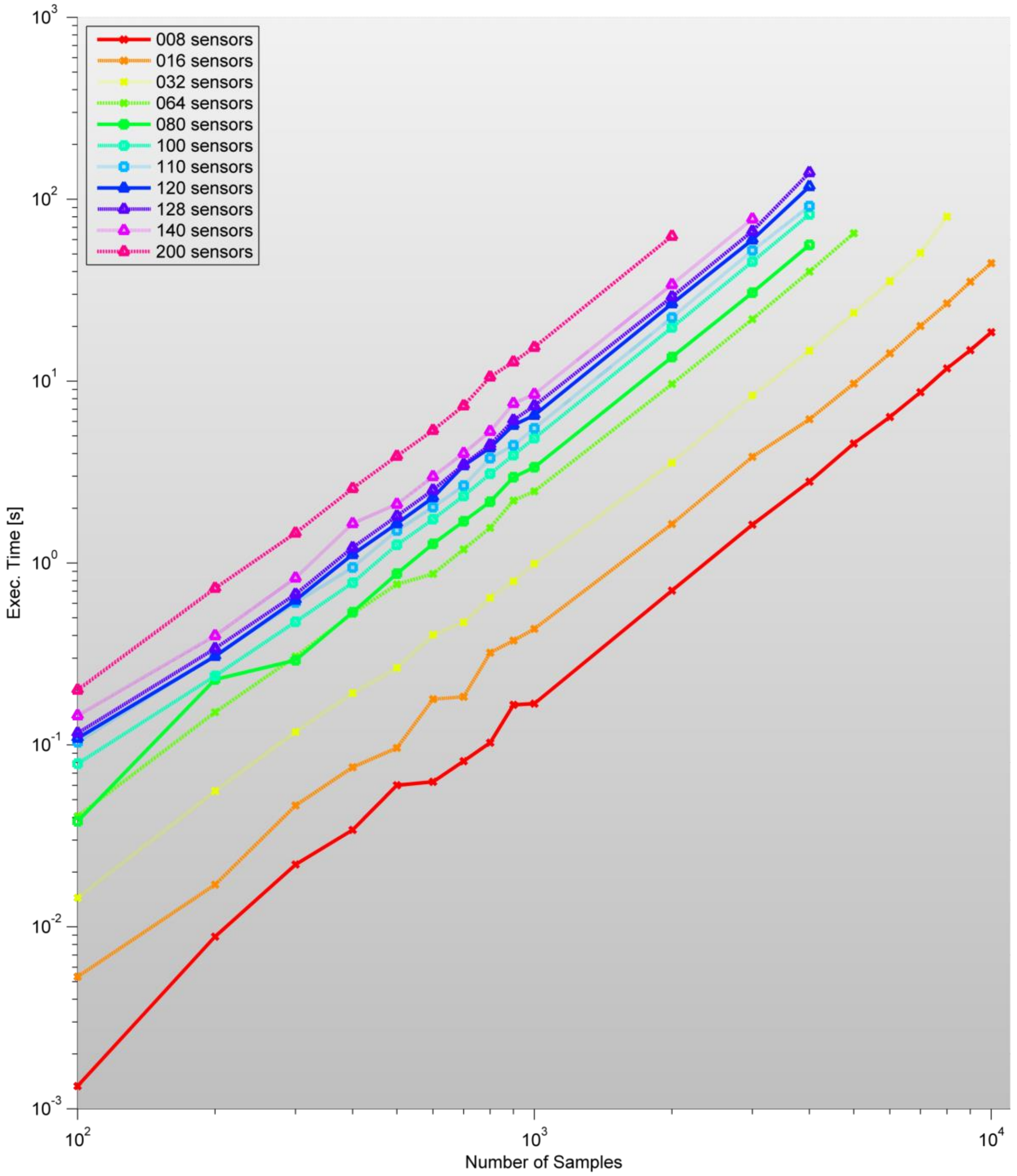

Figure 22 shows execution times for setup B, for C-mex implementation of GS indices.

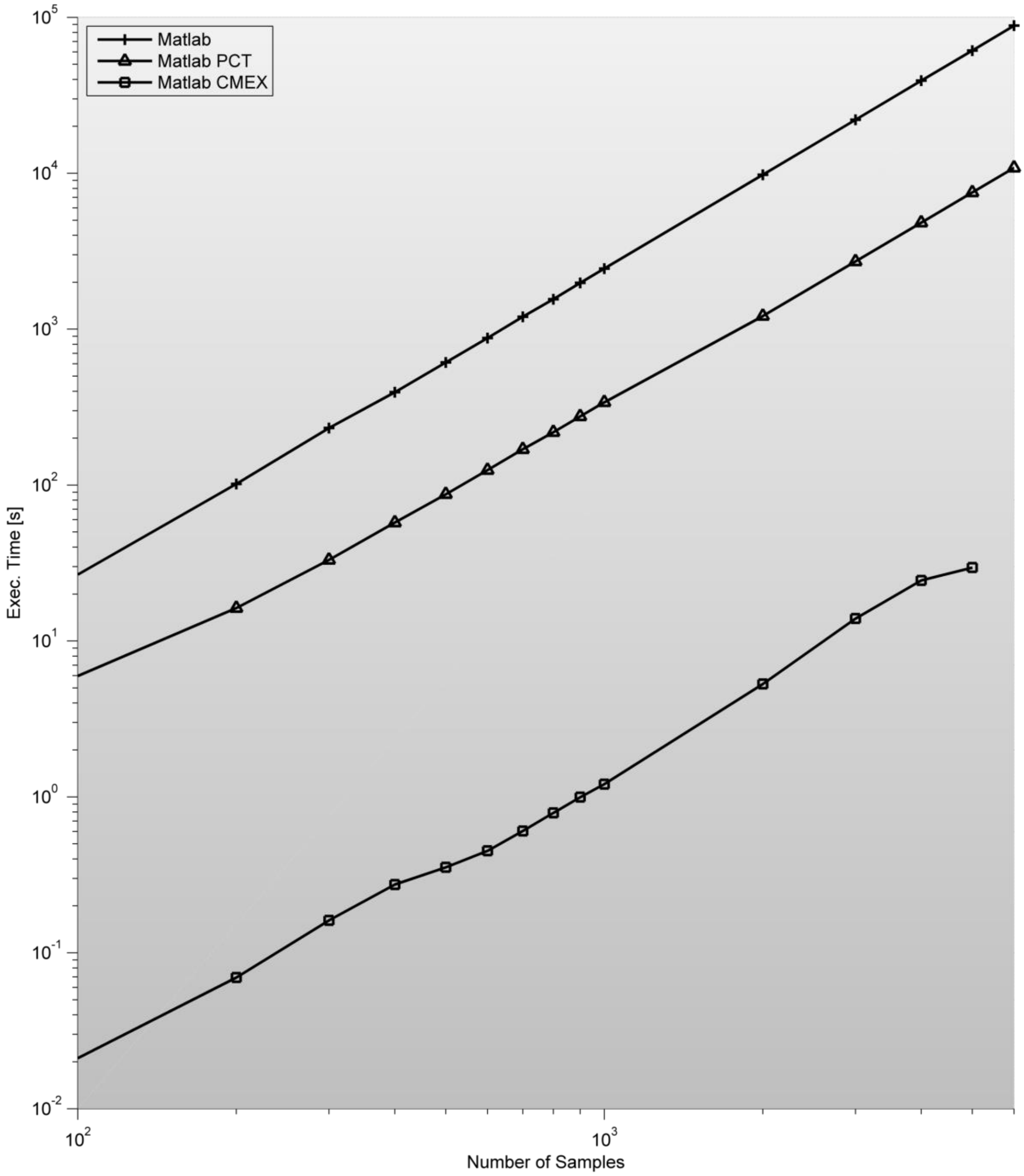

Figure 23 show execution times for setup A, comparing all the different implementations of Generalized Synchronization indices implementations for a setup of 64 channels and different number of sensors.

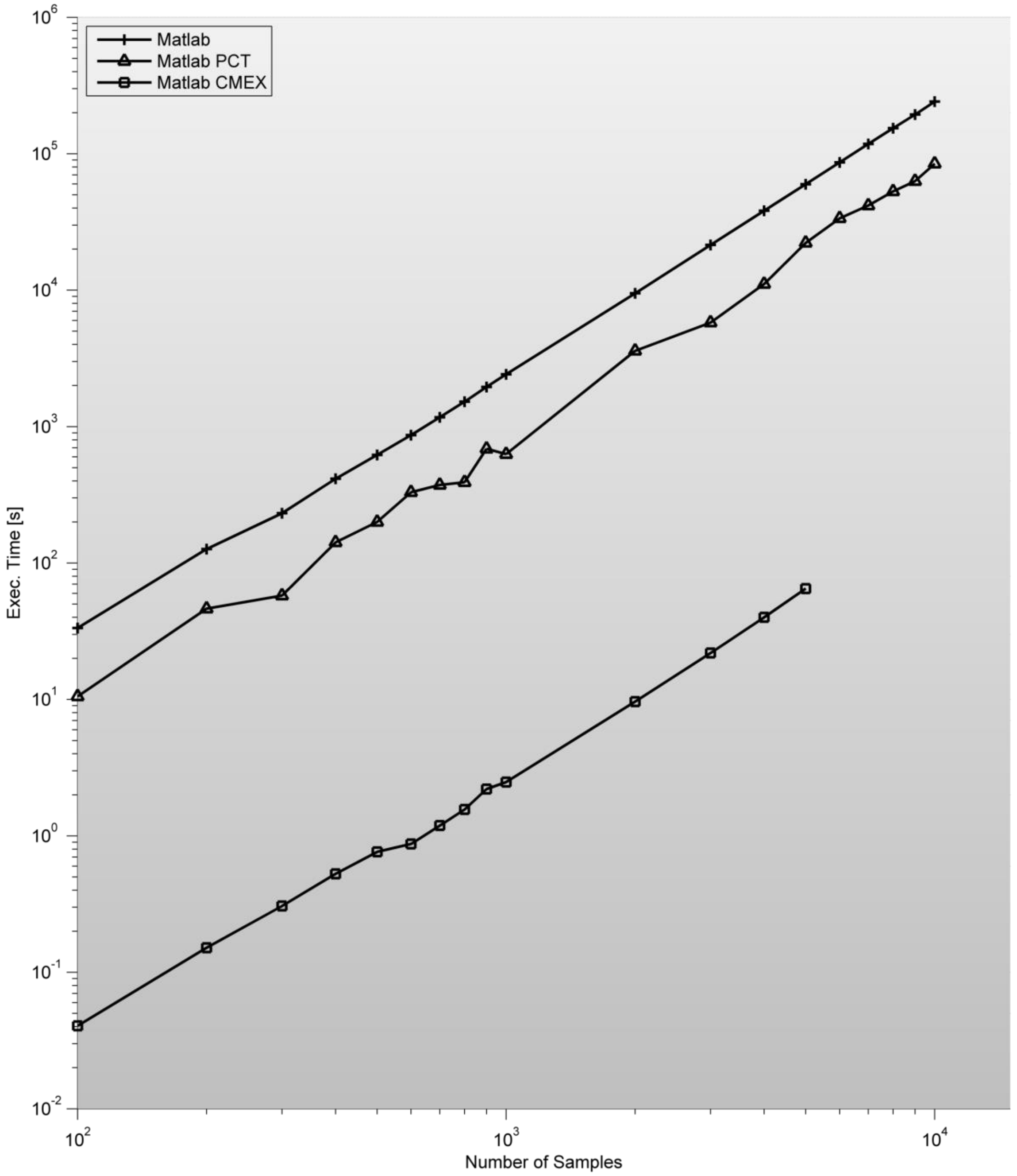

Figure 24 show execution times for setup B, comparing all the different implementations of Generalized Synchronization indices implementations for a setup of 64 channels and different number of sensors.

7. Network Measures

The following procedure was developed for timing measurements of the developed network indices Strength, Clustering Coefficient, Shortest Path Length, and Betweenness Centrality.

i. Generate random weighted adjacency network, as a normal distributions of real values between 0 and 1.
ii. Define a threshold of 0.1
iii. Make every weighted edge bellow threshold equal 0.
iv. Make every element in the principal diagonal equal 0.
v. Call network function.
vi. Call tic function.
vii. Call network function function.
viii. Call toc function.
ix. Repeat steps vi to viii 35 times.
x. Report mean average time spent in network index computation.

The function computing betweenness centrality also outputs the Shortest Path Length of the input network. This is so because while computing Betweenness it takes no extra time to compute at the same time Shortest Path Length. Thus, in case of needing both indices, it is always advisable to use Betweenness function only.

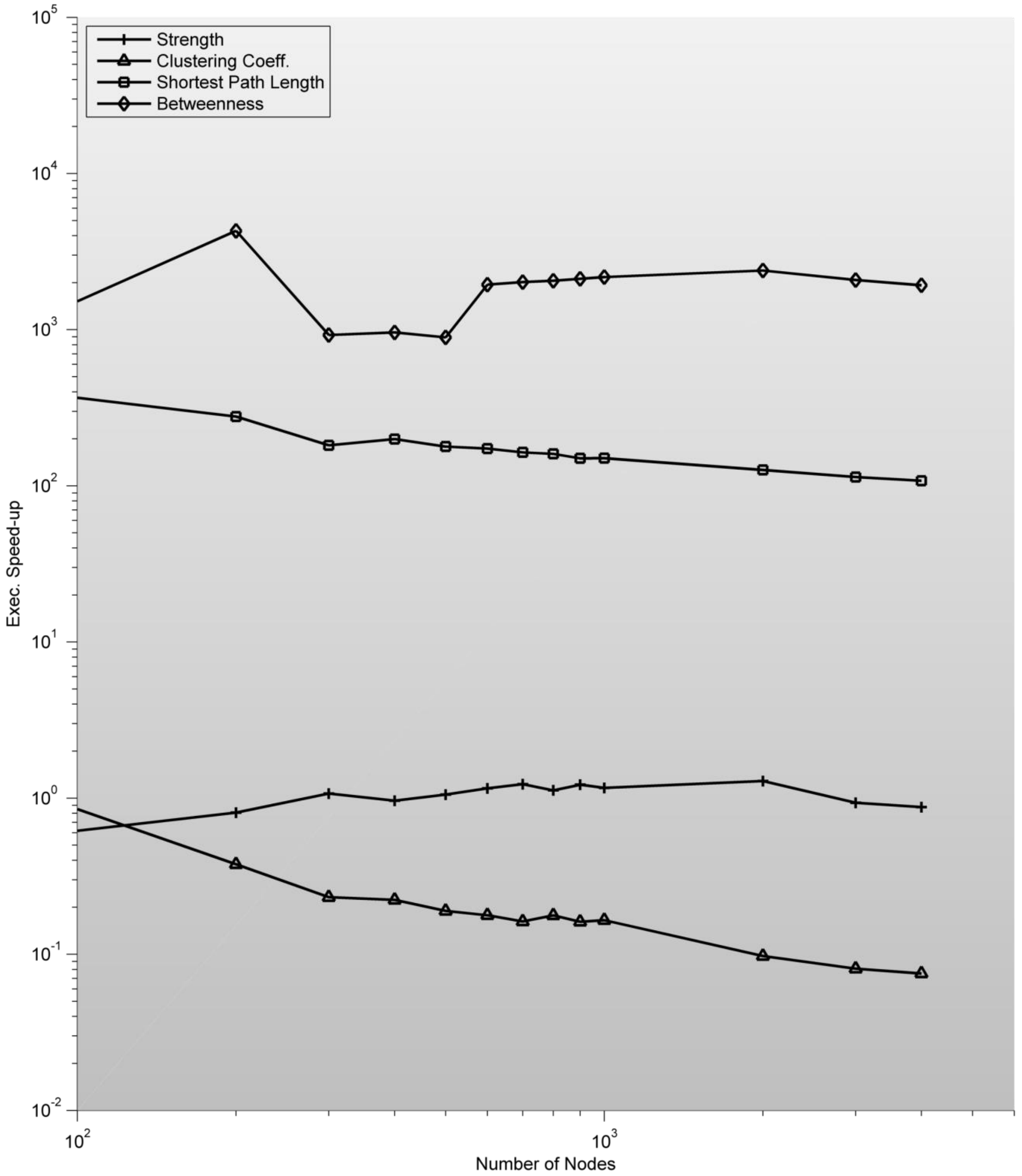

Figure 25 shows speedup ratio in setup A, between C-mex implementation of each index implemented in this work and previous implementations within Brain Connectivity Toolbox.

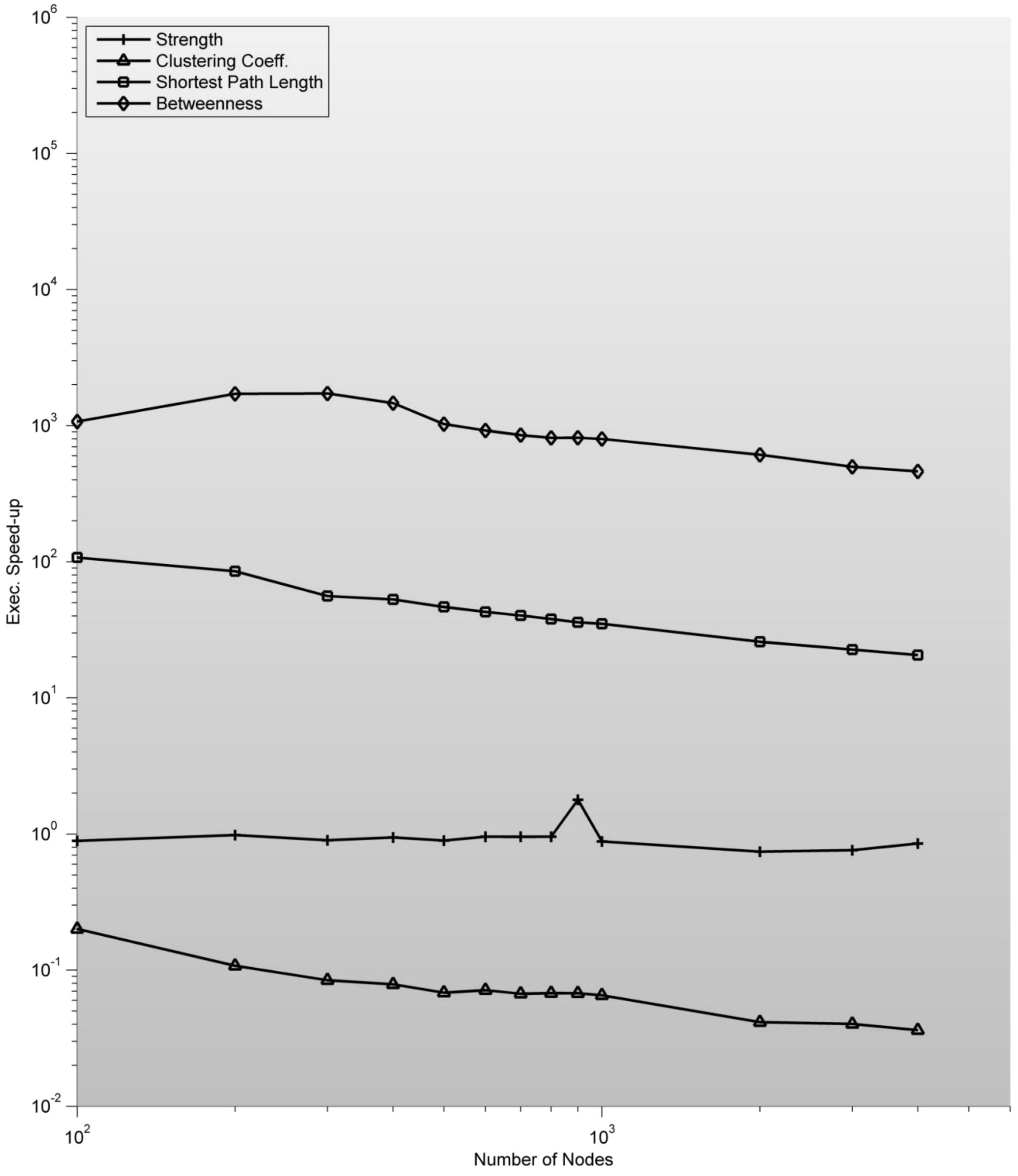

Figure 26 shows speedup ratio in setup A, between C-mex implementation of each index implemented in this work and previous implementations within Brain Connectivity Toolbox.

8. User Manual

During the following text the set of C implementation of Functional Connectivity Measures and Network indices will be called FastFC.

8.1. Who can use FastFC

FastFC is primarily intended to help FC EEG and MEG studies. Other methodologies dealing with neurophysiological signals might very easily benefit from these functions. However as long as the user is comfortable with the method, they can be applied to a very wide range of problems and related data.

It is intended to be used within Matlab environment and for that reason it has been implemented making use of Mathworks' external interface. However it has been developed mostly in straight compatible C code, and every function spans only one single file, so that adding these functions to another project should be easy. Besides, porting each implementation to other languages' interfaces through a C wrapper, should not be very difficult. Only C-mex restricted functions and names should be substituted with compatible C.

8.2. Installation

In general, users do not need any further installation provided you have the set of functions in your Matlab's path. It is therefore preferable to try out each function before installing any package. If a software package needs to be installed a "specified module could not be found" error message will appear in Matlab's command window.

The source-code and precompiled executables for Microsoft Windows 64bit Operating system are directly available in FastFC website: http://juangpc.github.io/FastFC/

A required library file of FFTW is also included in the release, although it is a good idea to check for updates.

Microsoft Windows library for multithread execution can be found here:

http://www.microsoft.com/en-us/download/confirmation.aspx?id=30679

FFTW library for Microsoft Windows 64bit systems can be found here:

http://fftw.org/install/windows.html

8.3. Developers

GitHub page for the FastFC project is https://github.com/juangpc/FastFC where the complete source-code is available. Each family of measures is separated in its own branch. Whenever an improvement is made to a function, the final compilation result is copied into the default branch called "release".

8.4. List of Functions

In general terms, Functional Connectivity matrices are computed compactly. Input data is conceived as the whole sensor matrix as a column ordered matrix with every sample and every sensor, in it, therefore each resulting connectivity matrix will be a squared matrix with the same number of rows and columns as the number of columns (number of sensors) of the input data. Avoiding, this way, the need for repeatedly calling Functional Connectivity functions for each pair of sensors.

This approach can derive in an *out-of-memory* problem when dealing with hardware setups with not enough memory, especially when dealing with lots of sensors or sample lengths in each channel (this is particularly the case of Generalized Synchronization algorithm which is very memory demanding). However, this problem can easily be circumvented by calling the function once for each pair of sensors, and collecting the Functional Connectivity index from the resulting squared matrix of size two.

Equally, all functions computing network indices compute the specified index for every node in the network. Resulting therefore in a single dimensional matrix with one value per node (row or column) in the Functional Connectivity matrix. This implementation tries to resemble as much as possible Brain Connectivity Toolbox behavior.

All functions in FastFC are directly accessible from the command line window of Matlab. During execution, the function polls the number of CPU cores present in the computer and progresses computations deploying an equal number of parallel threads.

Both functions using FFTW subroutine library are the zero phase distortion filter and Phase Synchronization. They both have an input parameter called *mode*, which allows deciding how exhaustive the search for the optimal FFT algorithm should be. This is a FFTW feature which has been maintained through FastFC. Sometimes it is a good idea to spend a few seconds or even minutes searching for a good algorithm for FFT that can significantly improve performance of subsequent executions. Typically an initial search at the beginning of each MATLAB session can take a few minutes, ensuring an efficient execution which last much less time in every subsequent call to the function. It is however easy to find other situations where a diligent and suboptimal execution of the function is more convenient.

8.4.1. Zero Phase Distortion Filter

y=fastfc_filt(filter,data,mode)

Input parameters:

filter = row vector with filter denominator coefficients.
data = column matrix with data to be filtered. Each sensor should be each column.
mode = mode for FFT scheduling
mode = 0 , execute fastest but suboptimal.
mode = 1 , execute fast but in a suboptimal algorithm.
mode = 2 , execute slower the first time, but consider possible faster algorithms.
mode = 3 , execute slowest the first time, but consider the fastest algorithm.

Output parameters:

y = array the same size of data, with each column corresponding to each filtered column of data.

8.4.2. Phase Synchronization

PLV. PLI. wPLI are all computed (along with the estimated significance of the PLV) within the MEX function *fastfc_ps* directly invoked from the MATLAB environment.

It is important to understand an additional input parameter of this function: *samples_to_discard* is the number of data samples to discard, to prevent edge effects during the estimation of the Hilbert Transform. The function uses an FFT-based algorithm to estimate the Hilbert Transform, after mirror padding the data, so that discarding of data should be used only for narrow signals where serious edge effects are suspected.

[plv,pval_plv,pli,wpli]=fastfc_ps(data,samples_to_discard,mode)

Input parameters:

data = data (sensors by columns).
samples_to_discard = samples to discard at the beginning and samples to discard at the end of the phase signals. i.e. To discard 200 samples at the beginning and 200 samples at the end of each sensor, samples_to_discard should equal 200.
mode = 0 -> execute fastest but suboptimal.
mode = 1 -> execute fast but in a suboptimal algorithm.
mode = 2 -> execute slower the first time, but consider possible faster algorithms.
mode = 3 -> execute slowest the first time, but consider the fastest algorithm.

Output parameters:

plv = Phase Locking Value Functional Connectivity matrix.

pval_plv = pvalue for each index of the PLV matrix.

Pli = Phase Locking Index Functional Connectivity matrix.

wPli = weighted Phase Locking Functional Connectivity matrix.

8.4.3. Mutual Information

The function implementing the calculation of MI index using k-nearest neighbours, and its corresponding input/output parameters, is defined below.

[mi]=fastfc_mi(data,emb_dim,tau,k);

Input parameters:

data = eeg data (sensors by columns).

emb_dim = embedding dimensions to consider

tau = time lag to consider for embedding

k = number of neighbours to consider

Output parameters:

mi = Mutual Information Functional Connectivity matrix

8.4.4. Generalized Synchronization

The function implementing the calculation of GS indices: S, H, M and L, and its corresponding input/output parameters, is defined below.

[S,H,M,L]=fastfc_gs(data,emb_dim,tau,k,w,states_eff_step)

Input parameters:

data = eeg data (sensors by columns).

emb_dim = embedding dimension

tau = time lag for embedding

k = number of neighbours to consider

w = window correction for neighbour finding

states_eff_step = state-space down sampling to consider when calculating distances

Output parameters:

S, H, M and L = Functional Connectivity matrices for each index.

8.4.5. Strength

[S]=fastfc_strength_wu(A)

Input parameters:

A = adjacency matrix of real values between 0 and 1, with zeroed principal diagonal elements.

Output parameters:

C = a row matrix where every value represents the Strength of each node.

8.4.6. Clustering Coefficient

[C]=fastfc_cluster_coef_wu(A)

Input parameters:

A = adjacency matrix of nodes by nodes. Values between 0 and 1. Principal diagonal is zero.

Output parameters:

C= column matrix where every value represents the Clustering Coefficient of each node.

8.4.7. Shortest Path Length

[D,L]=fastfc_shortest_path_length_w(W)

Input parameters:

W = directed or undirected weighted connection length matrix.Typically W is obtained by inverting each element in the Functional Connectivity matrix, transforming therefore between weights to length.

Output parameters:

D = distance matrix between nodes (shortest weighted path).

L = number of edges in each shortest weighted path.

8.4.8. Betweenness Centrality

Node Betweenness Centrality

[D,L,BC]=fastfc_betweenness_cent_w(W)

Input parameters:

W = directed or undirected connection-length matrix.

Output parameters:

D = distance matrix between nodes (shortest weighted path).

L = number of edges in each shortest weighted path.

BC = node Betweenness Centrality for each node in the network.
